# Improving the efficacy of exome sequencing at a quaternary care referral centre: novel mutations, clinical presentations and diagnostic challenges in rare neurogenetic diseases

**DOI:** 10.1136/jnnp-2020-325437

**Published:** 2021-06-08

**Authors:** Christopher Grunseich, Nathan Sarkar, Joyce Lu, Mallory Owen, Alice Schindler, Peter A Calabresi, Charlotte J Sumner, Ricardo H Roda, Vinay Chaudhry, Thomas E Lloyd, Thomas O Crawford, S H Subramony, Shin J Oh, Perry Richardson, Kurenai Tanji, Justin Y Kwan, Kenneth H Fischbeck, Ami Mankodi

**Affiliations:** 1 Neurogenetics Branch, National Institute of Neurological Disorders and Stroke, National Institutes of Health, Bethesda, Maryland, USA; 2 Departments of Neurology and Neuroscience, Johns Hopkins University School of Medicine, Baltimore, Maryland, USA; 3 Department of Neurology, Johns Hopkins University School of Medicine, Baltimore, Maryland, USA; 4 Department of Neurology, University of Florida, Gainesville, Florida, USA; 5 Department of Neurology, University of Alabama at Birmingham, Birmingham, Alabama, USA; 6 Department of Neurology, George Washington University, Washington, District of Columbia, USA; 7 Division of Neuropathology, Columbia University Medical Center, New York, New York, USA

## Abstract

**Background:**

We used a multimodal approach including detailed phenotyping, whole exome sequencing (WES) and candidate gene filters to diagnose rare neurological diseases in individuals referred by tertiary neurology centres.

**Methods:**

WES was performed on 66 individuals with neurogenetic diseases using candidate gene filters and stringent algorithms for assessing sequence variants. Pathogenic or likely pathogenic missense variants were interpreted using in silico prediction tools, family segregation analysis, previous publications of disease association and relevant biological assays.

**Results:**

Molecular diagnosis was achieved in 39% (n=26) including 59% of childhood-onset cases and 27% of late-onset cases. Overall, 37% (10/27) of myopathy, 41% (9/22) of neuropathy, 22% (2/9) of MND and 63% (5/8) of complex phenotypes were given genetic diagnosis. Twenty-seven disease-associated variants were identified including ten novel variants in *FBXO38, LAMA2, MFN2, MYH7, PNPLA6, SH3TC2* and *SPTLC1*. Single-nucleotide variants (n=10) affected conserved residues within functional domains and previously identified mutation hot-spots. Established pathogenic variants (n=16) presented with atypical features, such as optic neuropathy in adult polyglucosan body disease, facial dysmorphism and skeletal anomalies in cerebrotendinous xanthomatosis, steroid-responsive weakness in congenital myasthenia syndrome 10. Potentially treatable rare diseases were diagnosed, improving the quality of life in some patients.

**Conclusions:**

Integrating deep phenotyping, gene filter algorithms and biological assays increased diagnostic yield of exome sequencing, identified novel pathogenic variants and extended phenotypes of difficult to diagnose rare neurogenetic disorders in an outpatient clinic setting.

## Introduction

Patients with hereditary neuromuscular disorders often present with nonspecific, complex and atypical phenotypes, making a precise diagnosis based on clinical data alone difficult. The identification of disease genes enables molecular diagnosis of patients, defines disease risk in relatives and represents the first step to a better understanding of the physiological role of the underlying protein and disease pathways, which in turn leads to therapeutic developments for the diseases. Whereas targeted gene panels and candidate gene testing can identify known mutations, these tests may be costly and not sensitive to unknown mutations. Whole-exome sequencing (WES) allows rapid, low cost and unbiased identification of pathogenic variants in patients with suspected genetic diseases.^1–5^ But, the large amount of information provided by this powerful gene discovery tool can be time-consuming and often yields inconclusive results. The use of gene lists can improve diagnostic yield by filtering patient WES results to those variants identified in genes associated with disease-causing mutations.^6^ Yet, variants of interest identified through gene list filtering require further validation by additional methods.

The Neurogenetics Clinic at the National Institutes of Health (NIH) Clinical Centre is a quaternary care centre for patients referred from national and international tertiary medical centres. Referrals include complex and difficult to diagnose singleton or small familial cases in which standard care evaluations have not identified a diagnosis. In this study, we integrate gene filter lists with detailed phenotyping, biological assays and family segregation analysis to diagnose patients with rare neurogenetic disorders. This has allowed us to identify mutations in patients from diverse ethnic and racial backgrounds with a range of neurogenetic disorders including those that are rare, complex and atypical.

## Materials and methods

### Study population

A cohort of 66 patients (age range: 13–75 years, median: 48 years) with suspected hereditary neurological diseases had exome analysis as part of their diagnostic evaluation in the NIH Neurogenetics Clinic (Bethesda, MD). These patients were referred from tertiary medical centres and had completed an evaluation by an outside neurologist. This included 50 singleton cases (76%), 9 cases from 5 sibling pairs and 7 cases from multigeneration families. Seventeen patients (26%) reported ancestry from outside of Europe including Asia and South America. A majority of patients (85%; n=56) had previous genetic testing including single and panel gene testing. All had a thorough physical examination, and relevant clinical testing such as MRI, electrophysiology studies and muscle biopsy. Genetic counselling was provided. Family members were also evaluated when feasible. Informed written consent or assent was obtained from each subject before participation in the study.

### Exome analysis

WES was performed in a research laboratory (NIH Intramural Sequencing Center) on DNA extracted from whole blood. The NimbleGen SeqCap EZ V.3.0+ UTR capture kit (Roche, Basel, Switzerland) was used to cover 96 Mb of genomic DNA (100 ug/mL). The captured DNA library was sequenced using Illumina HiSeq to provide coverage to call most probable genotypes (MPGs) with a score of 10 in at least 85% of targeted bases. Reads were mapped to NCBI build 37 (hg19) using the ELAND (Efficient Large-scale Alignment of Nucleotide Databases;Illumina, San Diego, California, USA), and Novoalign V.3.02.07 (Novocraft, Selangor, Malaysia). The aligned bam files were fed as input to bam2mpg (http://research.nhgri.nih.gov/software/bam2mpg/index.shtml), to call MPGs at all covered positions using a probabilistic Bayesian algorithm. Genotypes with an MPG score ≥10 showed greater than 99.9% concordance with Illumina Human 1M-Quad single nucleotide polymorphism (SNP) Chip data. Sequence bases with Phred quality score >30 were included for the analysis.

### Variant identification and interpretation

The strategy of variant annotation, interpretation, and validation is highlighted in online supplemental figure 1. Exome variants were filtered using VarSifter (National Human Genome Research Institute, Bethesda, USA). Variants were computationally annotated using ANNOVAR, presence in dbSNP (version 137), the International Genome Sample resource (n=2504 genomes), NHLBI EVS dataset of 6500 individuals, the Human Gene Mutation Database as a ‘Disease Mutation’, and ClinVar, frequency within the Exome Aggregation Consortium Database (ExAC database; n=60 706 exomes), and predicted effect on the protein using CDPred, SIFT and Polyphen-2. Chromosomal SNP microarray analysis helped to focus ES screening in relevant cases. Trio exome sequencing was used when parental DNA was available.

10.1136/jnnp-2020-325437.supp1 Supplementary data

Candidate genes were first evaluated following gene variant annotation as indicated in online supplemental figure 1. We then applied diagnostic gene filters to the patient’s ES variants of interest to facilitate the genetic diagnosis (online supplemental table 1). Gene filters were compiled from previously reported disease-associated variants listed in the Leiden Muscular Dystrophy pages (Leiden University Medical Center, NL), Neuromuscular Disease Center (Washington University, St Louis, Missouri, USA), Online Mendelian Inheritance in Man (OMIM) databases and searches of medical literature. A total of 634 gene identifiers were categorised into the following categories based on their asserted association with human disease: myopathy (n=293), neuropathy (n=193) and motor neuron disease (n=148). The variants of interest were first filtered by gene list appropriate to their phenotype, then the other two gene lists were also applied to ensure the diagnostic yield of the neuromuscular disease gene panels.

Variants identified from gene list filtering were categorised based on the guidelines recommended by the American College of Medical Genetics and Genomics and Association for Molecular Pathology. Variants with evidence of benign impact, likely benign impact or uncertain significance were excluded from further analysis. Pathogenic or likely pathogenic missense variants with allelic frequency <0.0001 in the genome aggregation database (gnomAD) were interpreted using CADD, CDPred and ClinPred. The total of 422 candidate gene variants were identified (online supplemental data 1). Heterozygous variants with gnomAD allele frequencies between 0.0001 and 0.00003 in genes associated with an autosomal dominant pattern of inheritance were categorised as uncertain significance. Manual review of variant inspection and read sequence alignments was done using the Integrative Genomics Viewer. We compiled further evidence to support classification of pathogenicity from additional genetic testing, family segregation analysis, previous publications of asserted disease association and relevant biological assays in patient-derived cell lines and tissues. Clinically relevant variants were validated by a Clinical Laboratory Improvement Amendment CLIA-certified laboratory. Previously reported mutations were identified through VarSome, ClinVar, OMIM and PubMed.

10.1136/jnnp-2020-325437.supp2 Supplementary data

Where indicated whole-genome oligonucleotide array comparative genome hybridisation (CGH)+SNP analysis was performed by GeneDx using 180 000 oligonucleotide probes on blood DNA samples. RNAseq analysis of S.2 patient vastus lateralis muscle tissue was performed from total RNA using the Total RNA Library Prep Kit (Illumina). Sequencing of S.2 patient muscle tissue was performed on an Illumina HiSeq 2500 platform, with reads aligned to human reference (HG18). To generate ADSSL1- cDNA clones, cDNA was synthesised by reverse transcription of total cellular RNA isolated from the biopsied vastus lateralis muscle, followed by digestion with RNase H. cDNAs extending from exons 2 to 13 shared by most ADSSL1 splice isoforms were amplified through 31 cycles of PCR. Products from five PCR reactions were combined, gel-purified and cloned into pBSK for Sanger sequencing (n=30 clones). The alternate 5’UTR and coding sequence of exons 1 and 2 in the two prominent *ADSSL1* isoforms were amplified from genomic DNA and analysed by Sanger sequencing.

## Results

### Diagnostic yield of exome sequencing

**Figure 1:**
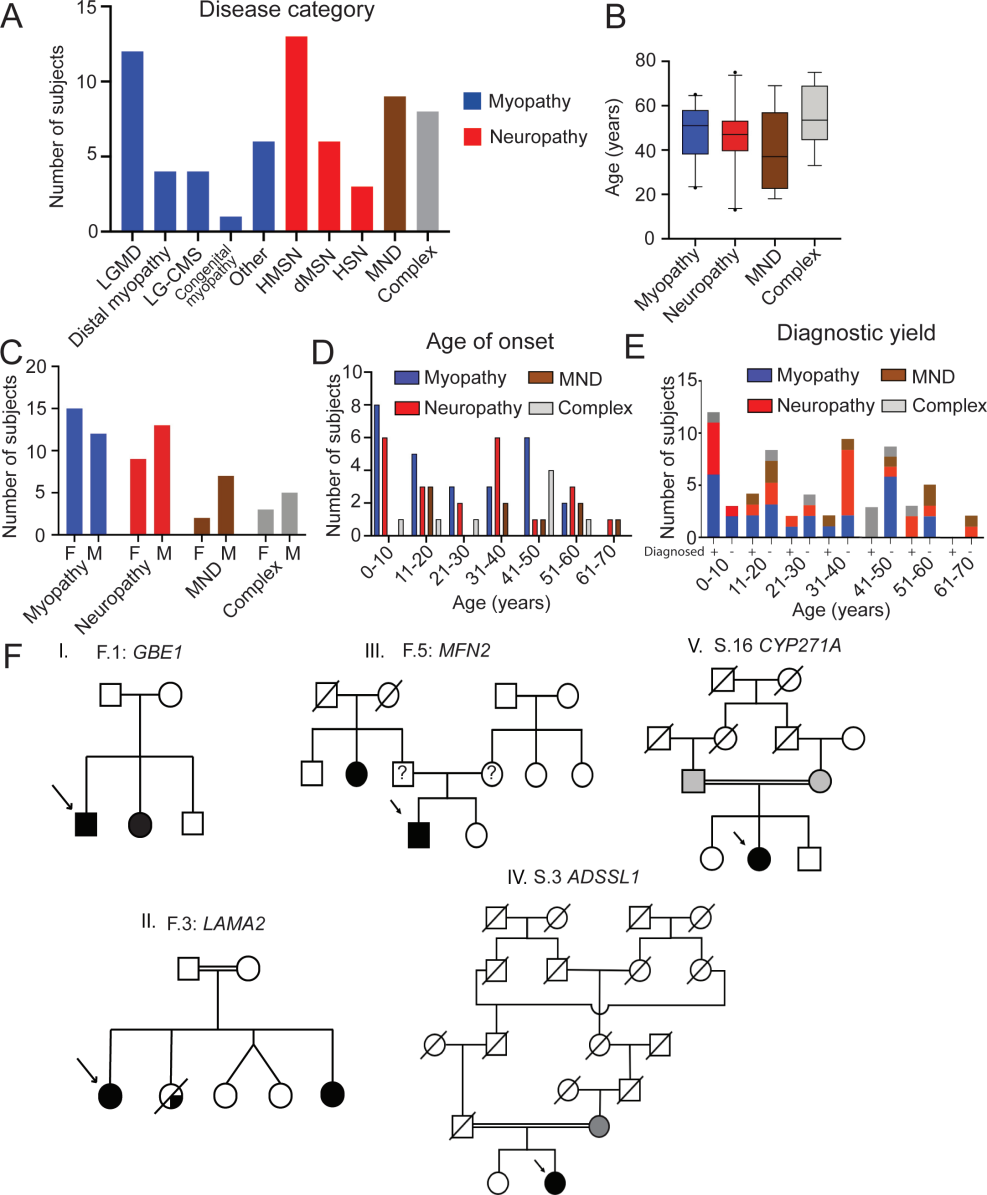
Demographics and inheritance patterns in those receiving WES. (A) The myopathy group (n=27) comprised of limb girdle muscular dystrophy (n=12), distal myopathy (n=4), limb girdle congenital myasthenic syndrome (LG-CMS; n=4), congenital myopathy (n=1), and other (n=6). The neuropathy group (n=22) consisted of hereditary motor and sensory neuropathy (HMSN, n=13), distal hereditary motor neuropathy (dHMN, n=6) and hereditary sensory neuropathy (HSN, n=3). These patients had a predominantly axonal (n=17), demyelinating (n=3) or mixed (n=2) patterns of nerve injury. There were nine patients in the motor neuron disease (MND) group and eight patients in the complex group. (B) Graph showing the distribution of ages in years at the time of initial presentation with mean and 5/95% CIs. Mean ages at the time of presentation are 48, 46, 41 and 55 years for the myopathy, neuropathy, MND and complex phenotype groups, respectively. (C) The number of females (F) and males (M) in each disease category. (D) Distribution of age of onset in each disease category. The myopathy group had 11 patients with juvenile/adult onset (10–40 years), 8 patients with childhood onset (<10 y) and 8 patients with late onset (>40 years) disease. The neuropathy group had 11 patients with juvenile/adult onset, 6 patients with childhood onset and 5 patients with late onset disease. The MND group consisted of five patients with juvenile/adult onset and four patients with late-onset disease. (E) Graph showing the number of diagnosed (+) and undiagnosed (−) cases in the myopathy (37%;10/27), neuropathy (41%;9/22), MND (22%;2/9) and complex (63%;5/8) phenotype groups by age group. (F) Representative pedigrees in families with multiple affected patients showing compound heterozygous mutations in *GBE1* (I, F.1), compound heterozygous mutations in *LAMA2* (II, F.3), heterozygous inheritance mutations in *MFN2* (III, F.5), homozygous mutations in *ADSSL1* (IV, S.3) and homozygous mutations in *CYP271A* (V, S.16). parents of proband in family F.5 not available for testing. WES, whole exome sequencing.

Demographics of 66 individuals including disease categories, age at evaluation, gender distribution and age of symptom onset are shown in figure 1. Overall, a genetic diagnosis was made in 39% (26/66) of patients tested, with the highest yield in patients with affected family members (88%, 14/16) and those with a complex phenotype (63%, 5/8; table 1, figure 1 (online supplemental table 2). Sixteen individuals (59%) with disease onset before 20 years of age received a genetic diagnosis, whereas the yield was relatively lower (27%) in older individuals with disease onset after 40 years of age. In those families with more than one individual affected, pedigrees included autosomal dominant and recessive inheritance patterns (figure 1).

**Table 1:** Novel pathogenic variants and phenotypes of individuals diagnosed through exome sequencing

Patient ID	Gene(Chr:position)c.DNA change p.protein change	Coding effect	CADD score	CDPred score	GnomAD freq.	GnomAD Allele count / # of het/ # of hom	ClinPred Score	Age at diagnosis (y):Sex	Phenotype (MIM#)
S.18	*FBXO38*(5:147 785 932) NM_030793.4:c.843T>G p.His281Gln	Het.	23.3	0	0	0/0/0	1.00	18:M	Juvenile-onset upper limb distal weakness; a de novo variant. HMN2D (615575)
S.4	*LAMA2* (6:129 636 905)NM_000426.3: c.3736-2A>T p.(?)	Hom.	34	−30	0.00001194	3/3/0	NA	69:F	Childhood-onset with milder phenotype, retained independent ambulation. LGMDR23 (618138)
F.3.1; F.3.2	*LAMA2*(6:129 204 451_129204 452)NM_000426.3:c.61_62delCA p.Gln21Glyfs*28	Het.	20.2	−30	0	0/0/0	NA	34:F; 24:F	Childhood-onset with milder phenotype, retained independent ambulation in sisters. LGMDR23 (618138)
S.13	*MFN2* (1:12 052 717)NM_014874.3:c.281G>T p.Arg94Leu	Het.	31	−2	0	0/0/0	0.99	38:F	Childhood-onset with severe disease, loss of ambulation by age 30y. CMT2A (609260)
F.5.1; F.5.2	*MFN2* (1:12 062 085) NM_014874.3:c.1085C>G p.Thr362Arg	Het.	24.3	−1	0	0/0/0	0.99	33:M;60:F	Adult-onset with milder, later onset disease in proband and his maternal aunt. CMT2A (609260)
S.7	*MYH7*(14:23 886 123) NM_000257.2:c.4598T>C p.Leu1533Pro	Het.	30	−9	0	0/0/0	0.99	65:M	Childhood-onset with foot drop at age 6 years. Loss of ambulation during his late 50s. A de novo variant. Laing distal myopathy (160500)
S.15	*PNPLA6*(19:7 615 957)NM_006702.4:c.2031G>T p.Glu677Asp; *PNPLA6* (19:7 619 463)NM_006702.4:c.2374G>C p.Gly792Arg	Comp. het.	17.8;23.6	−3; −2	0.00000679;0.00006377	1/1/0;2/2/0	0.18;0.92	19:F	Juvenile-onset motor neuron disease with upper >lower limb distal weakness; absent spasticity.
S.14	*SPTLC1*(19:7 619 463) NM_006415.2:c.1019C>T p.Ser340Leu	Het.	23.6	−2	0.00001592	4/4/0	0.92	68:M	Late-onset hereditary motor sensory neuropathy with mixed sensory and motor symptoms.

### Variant interpretation

**Figure 2:**
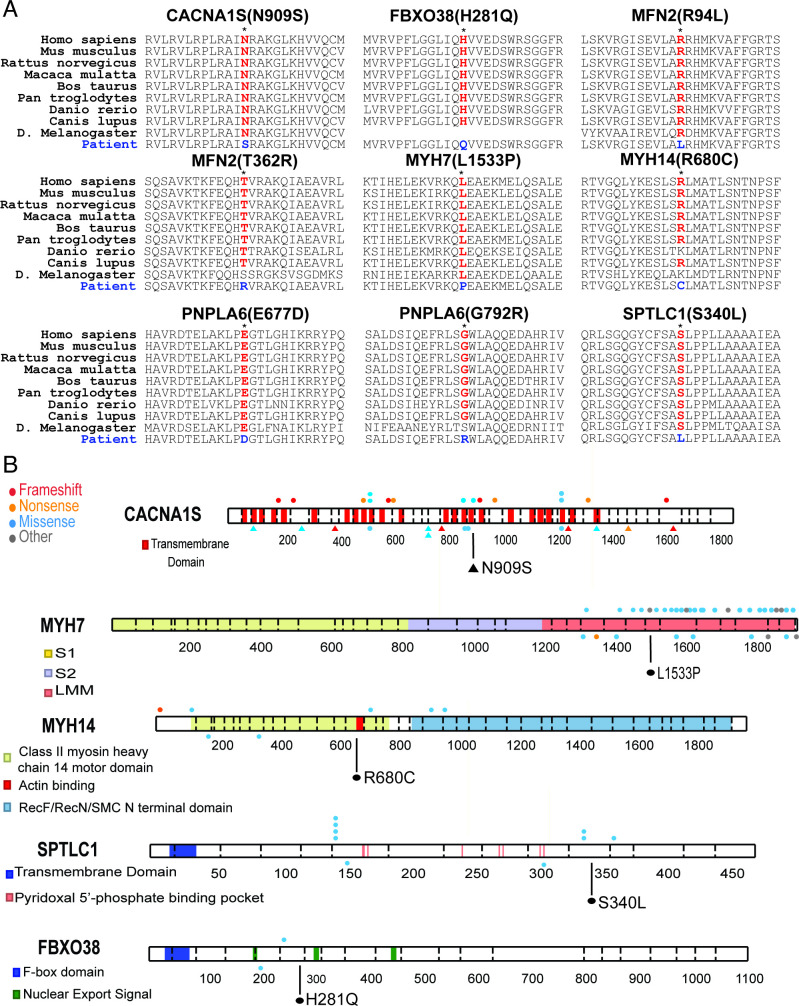
Gene variant characterisation. (A) Variants resulted in amino acid substitutions that were conserved across most vertebrates. Conservation was detected in *Drosophila melanogaster* in five of nine variants evaluated. (B) Variants occurred in hotspot regions containing previously reported disease causing variants. Mutations of CACNA1S associated with congenital myopathy are shown as triangles. For MYH7, only those variants in the light meromyosin (LMM) region are shown. Newly reported variants shown in black, ClinVar published variants indicated on top, and non-ClinVar published variants on bottom for each protein. Variant in MYH14 occurs within the actin binding domain.

Ten novel pathogenic variants were identified in patients with myopathy (n=3), neuropathy (n=5) and MND (n=2; table 1). Nine amino acid substitutions were in residues otherwise conserved across species (figure 2). These variants occurred in important functional domains of the proteins considered as hotspots for disease-associated variants. The *MYH7* p.Leu1533Pro variant in a distal myopathy patient (S.7) was positioned in the distal rod region of the protein where other mutations have been established to cause Laing distal myopathy and cardiomyopathies.^7^

Pathogenicity was also indicated by location near other previously reported mutations. The *SPTLC1* and *FBXO38* variants (patients S.14 and S.18) were in conserved regions containing clusters of previously published rare disease-associated mutations (figure 2).^8 9^ In two sisters (F3.1; F3.2) compound heterozygous loss-of-function variants were identified in the *LAMA2* gene including a novel frameshift expected to truncate the protein, and a previously reported pathogenic splice site mutation.^10^ Patient S.4 had a homozygous variant that destroyed the canonical splice acceptor site in *LAMA2* intron 25, and is presumed to cause loss of functional protein. A targeted exon-level oligo array CGH assay showed no complete or partial deletion and duplication of the *LAMA2* gene, supporting true homozygous splice site variant. Although *MFN2* mutations at p.Arg94 and p.Thr362 residues have been reported in CMT2A patients,^11^ the changes in our patients (S.13 and F.5.1;F5.2) with leucine and arginine substitutions, respectively, have not been previously reported. Segregation analysis for the variants was done by targeted variant testing in parents when available. To this end, we identified de novo dominant pathogenic variants in two cases (S.7, *MYH7*; S.18, *FBXO38*), and compound heterozygous mutations in one case (S.15, *PNPLA6*).

### Clinical presentations in patients with novel pathogenic variants

**Figure 3:**
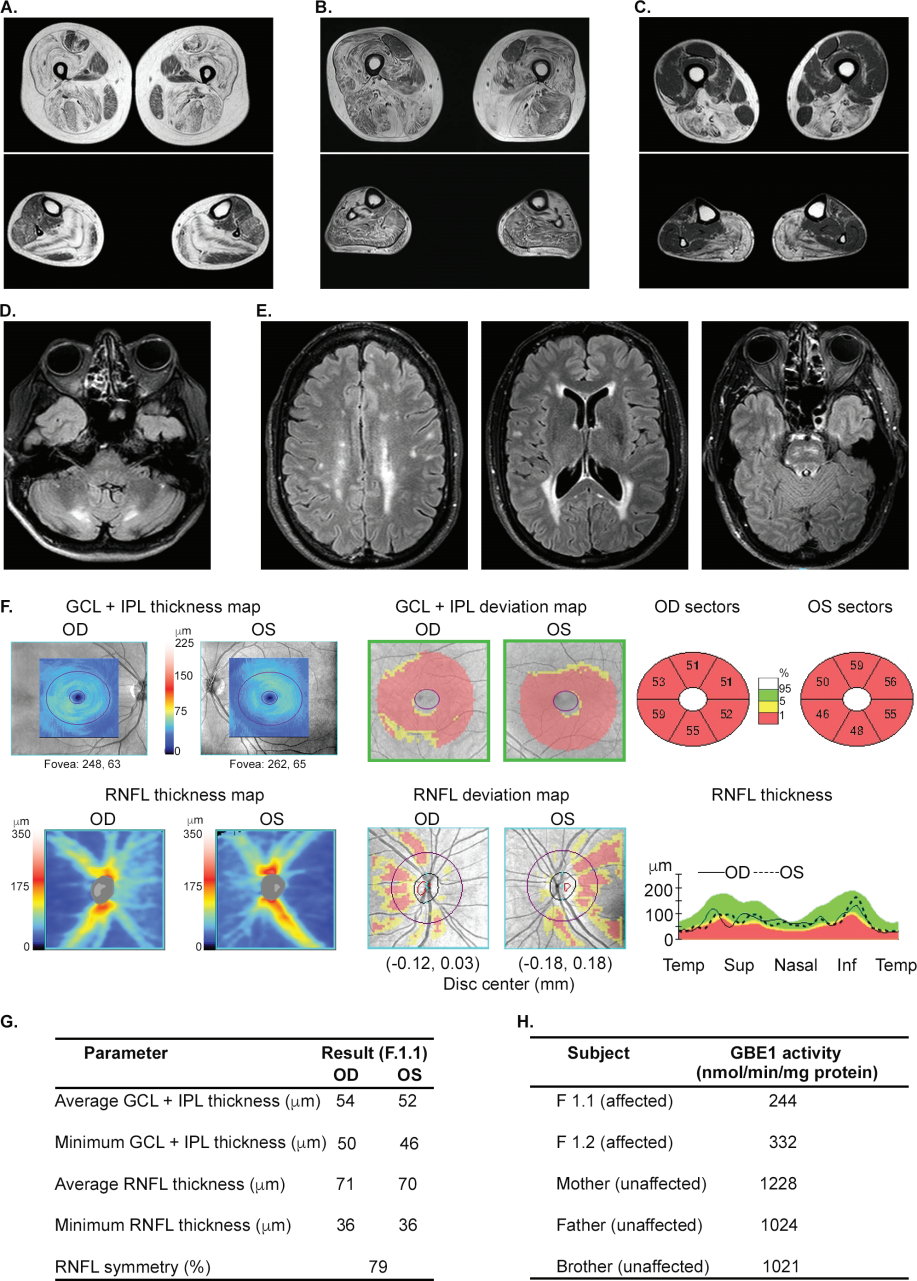
Examples of clinical test results aiding the genetic diagnosis by exome sequencing analysis. (A) T1-weighted axial images in F.3.1 patient with laminin α2 deficient LGMDR23 show peripheral fat replacement with central sparing in the vastus lateralis, soleus and gastrocnemius muscles, but the tibialis anterior is spared. The rectus femoris muscle shows increased signal around central fascia and periphery leaving a U-shaped less affected muscle. (B) The tibialis anterior is mostly replaced by fat, the quadriceps and adductor muscles are prominently affected, but the rectus femoris is spared in S.7 patient with Laing distal myopathy. In contrast with (A), multiple muscles show internal bands of fat infiltration. (C) Fat infiltration is seen in the adductor magnus, biceps femoris, semimembranosus, soleus and gastrocnemius muscles, followed by the semitendinosus, vastus and tibialis anterior muscles in S.2 patient with ADSSL1-related distal myopathy. (D) Typical symmetrical cerebellar white matter hyperintensities around dentate nucleus bilaterally in T2-FLAIR MRI in S.16 patient with cerebrotendinous xanthomatosis. optic neuropathy, leukoencephalopathy, and impaired cellular GBE1 activity in a family with adult polyglucosan body disease (APBD; E–G). (E) Axial T2-FLAIR images of brain show multiple discrete and confluent foci of high-signal in the subcortical white-matter, along the atria and occipital horns of the lateral ventricles, and along the white-matter tracts in the brainstem lesions. (F–G) Optical coherence tomography data showing bilateral atrophy of the macular ganglion cell-inner plexiform layer (GCL-IPL) and the retinal nerve fibre layer (RNFL), indicating neuronal death and axonal loss in optic nerves, respectively. Sup, superior; Inf, inferior. (H) The GBE1 enzyme activity measured in cultured skin fibroblasts of affected siblings and unaffected relatives.

Phenotypes of cases with novel variants are summarised in table 2 and in online supplemental data 2 section. A late-aged female (S.4), and adult sisters (F3.1, F3.2) presented with slowly progressive childhood-onset milder limb girdle dystrophy phenotype due to laminin-α2 deficiency (LGMDR23). There was no known parental consanguinity. Muscle MRI showed that the muscle periphery was primarily affected (figure 3), similar to a pattern seen in milder cases of LAMA-2 myopathies.^12^ Muscle biopsies were done more than 25 years previously and immunostaining was not analysed, thereby hampering the diagnosis until WES analysis. A late-aged male (S.7) with the *MYH7* variant presented with childhood-onset Laing distal myopathy.^7^ His exam showed distally predominant weakness with severe involvement of toe, finger and wrist extension, and ankle dorsiflexion and a pattern consistent with Laing distal myopathy on muscle MRI (figure 3). Cardiac echo and ECG were normal.

10.1136/jnnp-2020-325437.supp3 Supplementary data

**Table 2:** Individuals with established mutations and phenotypic extension diagnosed through exome sequencing

Patient ID	Gene(Chr:position)c.DNA change p.protein change	Coding effect	CADD score	CDPred score	GnomAD	ClinPred Score	Age at diagnosis (y):Sex	Phenotype (MIM#)
S.16	*CYP27A1*(2:219 678 909)NM_000784.3:c1183C>T p.Arg395Cys	Hom.	20.2	−14	0.0002831	0.60	33:F	Childhood-onset facial dysmorphism and skeletal anomalies; juvenile-onset cataracts, cognitive decline, neuropathy. CTX (213700)
F.2.1; F.2.2	*DOK7*(4:3 494 837_3 494 840)NM_173660.5:c.1124_1127dupTGCC p.Ala378Serfs*30	Hom.	N/A	N/A	0	N/A	56:M;52:M	Juvenile-onset steroid-responsive biceps weakness; milder limb-girdle weakness in brothers CMS10 (254300)
F.1.1; F.1.2	*GBE1(3:*81 691 938)NM_000158.3:c.986A>C p.Tyr329Ser; *GBE1*(3:81 542 964_81 542 972)NM_000158.3:c.2053–3358_2053- 3350delinsTGTTTTTTACATTACAGGT p.Tyr686Serfs*3	Comp. Het.	22;N/A	−11;N/A	0.000318;0	0.94;N/A	46:M;44:F	Adult-onset optic neuropathy, deafness, neuropathy, and white matter T2w hyperintensities in brother and sister. APBD (263570)
F.4.1; F.4.2	*GSN* (9:124 073 097)NM_000177.4:c.640G>T p.Asp214Tyr	Het.	22.2	−10	0	0.99	70:F; 47:M	Late-onset disease; bifacial weakness; lattice corneal dystrophy absent. Amyloidosis (105120)

APBD, adult polyglucosan body disease; N/A, not applicable.

An adult female (S.13) and F.5 family members with a *MFN2* variant presented with childhood onset and late onset CMT2A, respectively. A late-aged male (S.14) with a *SPTLC1* variant presented with distal weakness and loss of vibratory sensation in the lower extremities. He had symptoms of fatigue, muscle stiffness, and paresthesia. An adult male (S.18) with *FBXO38* mutation had juvenile-onset HMN2D^9^ with distal weakness prominently in finger flexion. The phenotype of patient S.11 with homozygous *SH3TC2* variants and CMT4C has been reported.^13^ An adult female (S.15) with a *PNPLA6* variant had juvenile-onset MND with distal weakness most marked in finger flexion, ankle dorsiflexion and toe extension.

### Phenotypic extension in patients with established pathogenic variants

Previously established pathogenic variants were identified in 17 patients (online supplemental table 2). Clinical examples with atypical presentations of rare neurological diseases are listed in table 2 and are described in online supplemental data 2 section. Two brothers (F2.1 and F2.2) with homozygous DOK7 mutation (CMS10)^14^ had a juvenile onset steroid-responsive weakness and EMG findings of small amplitude short duration motor unit potentials. The 2–5 Hz repetitive nerve stimulation test after the diagnosis of CMS10 showed 9%–17% decrement in the orbicularis oculi muscle. Their parents were second cousins. The initial presentation consisted of biceps muscle weakness during the teenage years, which remained predominant over the years causing disability. The effect of steroid treatment was noticeable on biceps muscle strength, and higher steroid doses were required as the disease progressed. Treatment with salbutamol/ephedrine and adding 3,4-diaminopyridine and gradual steroid taper was recommended, although no benefit was appreciated with a short trial of albuterol.^15^

Two members of a family (F.4.1; F.4.2) with hereditary gelsolin amyloidosis had facial paralysis, fasciculations, and weakness of other bulbar muscles. Their first symptom was bilateral facial weakness. Lattice corneal dystrophy was absent. Mild sensory neuropathy was present, and screening for systemic complications of amyloidosis was unrevealing. An adult female (S.16) with cerebrotendinous xanthomatosis (CTX), a rare potentially treatable disease, presented with asymmetric hypoplasia of facial structures, short neck, a missing rib, low average to borderline cognitive ability, and leukoencephalopathy (figure 3; online supplemental figure 2). Her major findings were abnormal facial appearance, poor scholastic performance and fatigue. Other features included sensorimotor demyelinating neuropathy and juvenile-onset cataracts. Cerebellar signs, spasticity and subcutaneous xanthomas were absent. A whole genome oligonucleotide array CGH and SNP genotyping detected several regions of extended homozygosity (>7 Mb) encompassing at least 90 Mb (3.1% or 1/32 of the genome), suggesting that the parents were first cousins, once removed. Exome sequencing identified a candidate recessive mutation within the region of homozygosity in the *CYP27A1* gene associated with CTX. The potential genetic contribution of loci in addition to *CYP27A1* was considered given the detection of multiple regions of homozygosity and several atypical features such as facial asymmetry, short neck and missing rib. Although the exome was analysed for genes outside of our neuromuscular gene filters, it is conceivable that a second unidentified genetic mutation has contributed to the phenotype. Both parents were asymptomatic heterozygous carriers. Plasma cholestanol levels were seven times higher than the reference value upper limit of normal. Treatment with chenodeoxycholic acid was recommended.^16^

An adult male (F1.1) with a heterozygous *GBE1* p.Tyr329Ser mutation associated with adult polyglucosan body disease (APBD), presented with distal paresthesias for 6 years, followed by erectile dysfunction, fatigue and progressive deterioration in vision in both eyes for 3 years. His exam showed bilateral optic atrophy and a left superior homonymous visual field defect suggestive of a postchiasmal lesion. Features included pyramidal tract signs in the lower limbs, sensory greater than motor axonal neuropathy with pes cavus, and sensorineural deafness. MRI showed leukoencephalopathy with multiple FLAIR signal hyperintensities at the supratentorial white matter and additional juxtacortical lesions. There were also lesions involving the bilateral superior cerebellar peduncle, as well as the left medullary pyramid (figure 3). Optical coherence tomography indicated optic nerve axon loss (figure 3). Prior to his evaluation at the NIH, he was diagnosed with multiple sclerosis and treated with interferon beta-1a for 2 years but continued to worsen. His sister (F1.2) had a similar but milder phenotype. The WES analysis showed a heterozygous c.986A>C, p.Tyr329Ser mutation in the *GBE1* gene and subsequently, Sanger sequencing of the entire gene identified a deep intronic deletion mutation in intron 15 (online supplemental table S2), which has been reported together with the p.Tyr329Ser mutation in APBD patients and resulted in markedly reduced GBE function.^17^ Family segregation studies showed that both mutations were on separate alleles. GBE activity in skin fibroblasts was 22% and 32% of the mean value in asymptomatic carriers in their family in the proband and his sister, respectively, confirming the diagnosis of APBD.

### Variants of uncertain significance

In several cases the candidate variants identified may be pathogenic, however, the allele frequency in gnomAD was higher than anticipated for autosomal dominant inheritance. An adult female (S.9) with *CACNA1S* variant (chr1:201035376, NM_000069.2:c.2725A>G, p.Asn909Ser) had congenital myopathy with severe generalised muscle weakness prominently affecting axial muscles. Nine heterozygotes with the *CACNA1S* variant identified in patient S.9 were present in gnomAD. The variant is in the S4 helix of the voltage-sensing transmembrane domain III in the Ca_v_1.1 channel (figure 1), where arginine mutations are associated with hypokalemic periodic paralysis.^18^ Both parents were asymptomatic and tested negative for the Asn909Ser variant. A gene panel testing for neuromuscular disorders did not reveal other mutation(s). A muscle biopsy was not available to examine the abnormalities in the T-tubules and sarcoplasmic reticulum, as reported previously in Ca_v_1.1 myopathy. Similarly, a late-aged female (S.10) with a *MYH14* variant (chr19:50760672, NM_024729.3:c.2038C>T, p.Arg680Cys) had a late onset complex disease with features of myopathy, neuropathy and deafness. Twenty-two heterozygotes with the *MYH14* variant identified in patient S.10 were present in gnomAD. The variant was located within its actin-binding domain that is highly conserved in all myosin proteins (figure 2).^19^

### Searching for a second mutation

In the case of patient S.2 with a single heterozygous *ADSSL1* mutation (chr14:105207568, NM_199165.2:c.910G>A, p.Asp304Asn), a second mutation in the *ADSSL1* gene has not been identified. The patient had symptom onset in his 50s of mild distal myopathy predominantly involving the posterior thigh and calf muscles (figure 3). This is in contrast to a severe childhood-onset presentation in patient (S.3) with consanguineous parents (second cousins, once removed) found to have the same mutation in homozygous state and in previously reported patients with juvenile-onset disease with homozygosity^20^ and compound heterozygosity.^21^ WES analysis of both parents for patient S.2 identified the mutation in his mother, suggesting germline transmission of the carrier status. A vastus lateralis muscle biopsy showed chronic myopathy with rimmed vacuoles (online supplemental figure 3). Gene panel testing was uninformative. It is possible that a second mutation exists and was not identified, although a search for another mutation in *ADSSL1* with a targeted array CGH assay and evaluation of total transcript levels and Sanger sequencing of cDNA clones extending from the second to the final exons shared by most ADSSL1 splice isoforms (n=30) and of the alternate 5’UTR and exons 1 and 2 in the two most prominent ADSSL1 isoforms from genomic DNA in the biopsied vastus lateralis muscle was nonrevealing. RNAseq analysis of the biopsied muscle tissue showed an even distribution of sequencing reads across the *ADSSL1* coding region with no truncation or pseudoexon inclusion and heterozygosity at codon 304 (online supplemental figure 4). For patient S.3, an oligonucleotide array CGH+SNP showed no partial or complete deletion of the *ADSSL1* gene locus, supporting true homozygous missense mutations. Her mother was a carrier for the same mutation. Her father was deceased, and his DNA was not available for testing. Other family members of S.2 and S.3 patients lived outside of the USA and were not available for segregation testing.

## Discussion

Whereas WES is well established in research and diagnostic laboratories, additional methods and approaches are needed for the interpretation and confirmation of this data, and the interface between the neurologist and the patient needs to be better described so that more physicians can decide which next steps are appropriate in the subsequent evaluation and validation of genetic testing data. Our approach used an array of tools feasible in clinical practice ranging from predictive algorithms, bioinformatics, family segregation studies, clinical diagnostic tests, and biological assays, which enhanced the yield of WES testing. We were able to obtain a molecular diagnostic yield of 39% (n=26) in 66 sequential, unselected individuals with diverse presentations of rare neurogenetic disorders, which is higher than the positive rates of other studies (13%–30%).^6 22^ These patients were without a diagnosis for many years despite extensive evaluations including single and panel gene testing for their conditions. Previous studies have demonstrated diagnostic success rates of 46% and 39% in cohorts receiving WES during childhood.^23 24^ Our diagnostic yield in those who presented below 20 years old was 59%, highlighting the novelty and utility of our approach. Late age of onset was a diagnostic challenge in our cohort, with a third of all patients having an age of onset over 40 years old. Within this patient group we were able to achieve a diagnostic success rate of 27%.

With the availability of WES technology, the curation of gene filter sets appropriate for the disease phenotype is an important step in variant interpretation. Filtering of variants using targeted gene lists containing a total of 634 genes applied broadly across the phenotype spectrum helped in reaching the molecular diagnosis in our patients. For example, we were able to detect pathogenic variants in *CYP27A1*, *GBE1*, *GSN*, *HEXB*, and *PNPLA6* genes, which have been previously associated with rare, diverse clinical presentations,^25–28^ and many of these genes are not typically included in diagnostic gene panel testing for specific diseases. We believe that our approach is more successful and cost effective than panel testing. Accordingly, we were able to diagnose 11 of 23 patients with previous negative gene panel sequencing.

Testing family members enhanced the diagnostic yield of WES even in singleton cases. Parental DNA testing determined de novo heterozygous point mutations in *FBXO38*, *GARS*, *MFN2*, and *MYH7* genes, and germline transmission on separate alleles for recessive mutations, such as in *ADSSL1*, *CYP27A1, GBE1,* and *PNPLA6* genes. The location of variants in the context of conserved functional domains and mutation ‘hot-spots’ predicted pathogenicity in previously undescribed variants such as *MYH7*. The *MYH7* gene encodes slow/β-cardiac myosin heavy chain, the motor protein of the sarcomere thick filaments which is expressed in type 1 skeletal muscle fibres and the heart ventricles. The mutated *MYH7* Leu1533 residue occupied a strategic position within the light meromyosin region of the myosin rod at which the substitution of proline likely disrupted coiled-coil structure and thus affected thick filament assembly.^7^

For recessive diseases, homozygosity was validated for variants in *ADSSL1*, *CYP27A1*, *DOK7*, *LAMA2* and *SH3TC2* genes through parental testing, SNP homozygosity mapping, and the absence of large gene deletions by CGH array analysis. In cases with compound heterozygous mutations, the search for a second mutation involved genetic testing beyond WES analysis, including deep gene sequencing, CGH array and tissue-derived transcript-level analysis. This is exemplified by the individuals with variants in *ADSSL1*, *GBE1* and *HEXB* genes. The WES analysis also led to valuable insights into the potential disease-modifying influence of additional genetic variations.^29^ In F.4 family with *HEXB +SH3TC2* variants, the reduction of SH3TC2 levels may have modified the presentation of Sandhoff disease and exacerbated the patient’s neuropathy.^30^ When applicable and feasible, we used blood and tissue samples from patients to validate the biological consequence of the candidate pathogenic variant. For example, impaired GBE1 activity in fibroblasts of F1.1 and F1.2 patients, and elevated plasma cholestanol levels in S.16 patient confirmed the loss of enzymatic function resulting from underlying mutations causing APBD and CTX, respectively.

WES analysis extended phenotypes in patients with novel as well as established disease-associated variants. The presenting feature of late-onset facial weakness without corneal lattice dystrophy is atypical in patients with gelsolin amyloidosis.^27 31^ Similarly, skeletal anomalies and facial dysmorphism are not typically associated with CTX, whereas more common features such as cerebellar ataxia, spasticity and subcutaneous xanthomas were absent in our patient. A history of parental consanguinity, juvenile cataracts, demyelinating neuropathy and cerebellar white-matter changes on MRI supported the diagnosis. Bilateral optic neuropathy as a prominent and presenting feature together with multifocal brain white matter hyperintensities led to an initial misdiagnosis of multiple sclerosis in our APBD patient, which is not unusual for this rare pleomorphic disorder.^32^ Optic neuropathy has not previously been described in patients with APBD. A naturally occurring orthologue of human GBE1 deficiency in Norwegian forest cats caused mild to moderate storage of abnormal glycogen in retinal ganglion cells with degeneration of the optic nerve,^33^ suggesting that the optic nerve is vulnerable to mutation effects in APBD. The phenotype of S.14 patient with a variant in *SPTLC1* is atypical and not found in in other HSAN1A cases. However, recent evidence has suggested that mutations in *SPTLC1* are linked to motor phenotypes including juvenile ALS.^34^ Interestingly, atypical phenotype of growth retardation, hypotonia, and vocal cord paralysis was reported in a patient with the p.Ser331Phe mutation in *SPTLC1*, which is close to the p. Ser340Leu variant identified in this study.^35^

Most importantly, molecular diagnosis allows clinicians to treat and more accurately predict prognosis. Using our approach, we were able to diagnose rare and potentially treatable diseases such as CMS10 and CTX.^14 16^ Furthermore, systemic complications of disease can also be anticipated and mitigated, which have prognostic implications and impact quality of life and survival. For example, screening for systemic amyloidosis in patients with gelsolin mutation, as well as cardiac and respiratory disease in patients with mutations in *MYH7*, *DMD* and *LAMA2* genes. Finally, a molecular diagnosis paves the way for disease modelling and precision medicine approaches in rare and disabling disorders with no currently available treatment.

The major limitations of WES include the inability to interrogate many variants that occur in regions of genome not currently recognised as a functional or regulatory region or resulting in large genomic reorganisations such as repeat expansions, deletions or duplications. Incomplete capture and coverage of genes may result in a failure to make a molecular diagnosis. Gene filters may not allow for detection of mutations in genes not previously known to cause a specific disorder. The algorithms may be limited by low concordance due to inherent biases and dependency on training datasets.

## Conclusion

WES analysis involves convergence of human and computer-assisted analysis. In this study, we demonstrate that detailed phenotyping, family segregation studies and biological assays together with stringent application of algorithms and use of candidate gene filters improved the diagnostic efficiency of WES in patients with complex atypical presentations of rare neurogenetic diseases. This approach allowed us to redefine our practice, identify new disease-associated variants, expand the phenotypic spectrum of rare diseases and offer better care to our patients and at-risk family members.

## Data Availability

Data are available on reasonable request.
