# Supplementary material for: Improving the efficacy of exome sequencing at a quaternary care referral centre: novel mutations, clinical presentations and diagnostic challenges in rare neurogenetic diseases
Source: J Neurol Neurosurg Psychiatry. 2021 Jun 8;92(11):1186–96. doi: 10.1136/jnnp-2020-325437 (PMC8522445; doi:10.1136/jnnp-2020-325437)

Supplemental table 1. List of candidate gene filters used in the analysis of exome sequencing.

MYOPATHY	NEUROPATHY	MND
ABHD5	AAAS	AAAS
ACADL	AARS1	AARS1
ACADM	ABCA1	AGT
ACADS	ABCD1	ALAD
ACADVL	ABHD12	ALS2
ACTA1	ADCY6	ANG
ADSSL1	AFG3L2	APEX1
AGL	AIFM1	APOE
AGPAT2	AMACR	AR
AGRN	ANG	ASAH1
AIRE	AP1S1	ATM
ALDOA	APOA1	ATP7A
ALG14	APTX	ATXN2
ALG2	ARHGEF10	ATXN3
ALG3	ARL6IP1	B4GALT6
ANKRD2	ARSA	BCL11B
ANO5	ASAH1	BCL6
ASCC1	ATL1	BICD2
ATGL	ATL3	BSCL2
ATP2A1	ATM	C19orf12
ATRN	ATXN1	C9orf72
B3GALNT2	ATXN10	CCS
B3GNT2	ATXN2	CDH13
BAG3	ATXN3	CDH22
BIN1	ATXN7	CHCHD10
BSCL2	B2M	CHMP2B
BVES	B4GALNT1	CNTF
CACNA1S	BAG3	CNTN4
CAPN3	BCKDHB	CNTN6
CASQ1	BSCL2	CRIM1
CAV1	C12orf65	CRYM
CAV3	C9orf72	CSNK1G3
CAVIN1	CLP1	CST3
CCDC78	CNTNAP1	CUL4B
CDKN1C	COX10	CYP2D6
CFL2	COX6A1	DAO

CHAT	CPOX	DCAF15
CHCHD10	CRYAB	DCTN1
CHD7	CTDP1	DIAPH3
CHKB	CTSA	DISC1
CHN1	CYP27A1	DNAJB2
CHRM3	DARS2	DOC2B
CHRNA1	DDHD1	DPP6
CHRNB1	DGUOK	DYNC1H1
CHRND	DHH	EFEMP1
CHRNE	DHTKD1	ELP3
CIDEC	DMD	EPHA4
CLCN1	DNAJB2	EWSR1
CLN3	DNAJC3	EXOSC3
CNBP	DNM2	FBLN5
CNTN1	DYNC1H1	FBXO38
COA3	EGR2	FEZF2
COL12A1	EMD	FGGY
COL13A1	ERCC6	FIG4
COL6A	ERCC8	FUS
COL6A1	FAH	GARS1
COL6A2	FAM126A	GBE1
COL6A3	FBLN5	GMPPA
COL9A3	FGD4	GRB14
COLQ	FGF14	GRN
COX10	FIG4	HEXA
COX15	FLNC	HFE
CPT2	FLRT1	HINT1
CRAT	FLVCR1	HSPB1
CRPPA	FMR1	HSPB3
CRYAB	FUS	HSPB8
CTNS	FXN	IGHMBP2
DAG1	GALC	ITPR2
DECR1	GAN	KDR
DES	GARS1	KIFAP3
DGUOK	GBA2	KLHL9
DIH1	GBE1	LAMA2
DMD	GDAP1	LAS1L
DMPK	GJB1	LIF
DNAJB6	GJB3	LIPC
DNAJC19	GLA	LOX

DNM2	GNB4	LUM
DNMT3B	GSN	MAOB
DOK7	HADHA	MAPT
DPAGT1	HADHB	MATR3
DPM2	HARS1	MT-ND2
DPM3	HINT1	MYH14
DTNA	HK1	NAIP
DURS1	HMBS	NEFH
DUX4	HOXD10	NETO1
DYSF	HSD17B4	NT5C1A
ECEL1	HSPB1	ODR4
EMD	HSPB8	OGG1
ENO3	IFRD1	OMA1
EPM2A	IGHMBP2	OPTN
ETFA	INF2	PCP4
ETFDH	KARS1	PFN1
FHL1	KIF1A	PGRN
FKBP14	KIF1B	PLEKHG5
FKRP	KIF1C	PNPLA6
FKTN	KIF5A	PON1
FLAD1	LAMA2	PON2
FLNC	LDB3	PON3
FOXL2	LITAF	PRPH
GAA	LMNA	PSEN1
GBE1	LRSAM1	PVR
GFPT1	LYST	RAMP3
GK	MAF	RBM28
GMPPB	MARS1	RBMS1
GNE	MED25	REEP1
GYG1	MFN2	RNF19A
GYS1	MMACHC	SCN7A
HACD1	MPV17	SCN9A
HADH	MPZ	SCO2
HADHA	MT-ATP6	SCP2
HADHB	MT-ATP8	SELL
HK1	MT-RNR1	SEMA6A
HNRNPA1	MT-TK	SETX
HNRNPA2B1	MT-TL1	SIGMAR1
HNRNPDL	MTMR2	SLC1A2
HOXA1	MTTP	SLC52A2

HSPB8
HSPG2
IGHMBP2
INPP5K
INSR
ISCA1
ISCA2
ITGA7
KBTBD13
KCNJ6
KIF21A
KLHL40
KLHL41
KLHL9
KY
LAMA2
LAMA5
LAMB2
LAMP2
LARGE1
LDB3
LDHA
LIMS2
LPIN1
LMNA
LMNB2
LMOD3
LONP1
LRP4
MADD
MAFB
MAP3K20
MATR3
MDM1
MECR
MEGF10
MGCA1
MSC
MSTN
MSTO1
MYH14
MYOT
NAGA
NALCN
NDRG1
NEFL
NF2
NIPA1
NOP56
OAT
OPA1
OPTN
PANK2
PDHA1
PDK3
PDYN
PEX1
PEX10
PEX7
PHYH
PLA2G6
PLEKHG5
PLOD1
PLP1
PMM2
PMP2
PMP22
PNKP
PNPLA6
POLG
PPOX
PPP2R2B
PRNP
PRPS1
PRX
RAB7A
RNASEH1
RRM2B
SACS
SBF1
SLC52A3
SLC5A7
SMN1
SMN2
SNCG
SOD1
SOD2
SOX5
SPAST
SPG11
SPG7
SPTLC1
SQSTM1
SUSD1
SYT9
TAF15
TARDBP
TBK1
TRPV4
TUBA4A
UBA1
UBQLN2
UNC13A
VAPB
VCP
VDR
VEGFA
VPS54
VRK1
ZFP64
ZFYVE26
ZNF746

MTAP	SBF2
MTM1	SCO2
MTMR14	SCP2
MUSK	SEPTIN9
MYBPC3	SETX
MYF6	SH3TC2
MYH14	SLC12A6
MYH2	SLC25A19
MYH7	SLC52A2
MYO18B	SNAP29
MYO9A	SOD1
MYOT	SOX10
MYOZ1	SPART
MYPN	SPAST
NADK2	SPG11
NALCN	SPG7
NEB	SPTLC1
NHLRC1	SPTLC2
P4HA1	ST3
PABPN1	SURF1
PFKM	TARDBP
PGAM2	TDP1
PGK1	TFG
PGM1	TRAF3
PHKA1	TRIM2
PHKB	TRPV4
PHOX2A	TSFM
PIEZO2	TTPA
PIK3R1	TTR
PLEC	TUBB3
PLIN1	TWINK
PNPLA2	TYMP
POGLUT1	UBQLN2
POLD1	VCP
POLG	XPA
POMGNT1	YARS1
POMGNT2	ZFYVE26
POMT1	
POMT2	
PPARG	

PREPL
PRKAG2
PSMB8
PYGM
PYROXD1
RAPSN
RBCK1
ROBO3
RPH3A
RS1
RXYLT1
RYR1
SALL4
SBDS
SCN4A
SCO1
SCO2
SECISBP2
SELENON
SETX
SGCA
SGCB
SGCD
SGCE
SGCG
SGCZ
SLC18A3
SLC22A5
SLC23A20
SLC25A1
SLC25A20
SLC25A26
SLC25A3
SLC25A4
SLC25A46
SLC5A7
SMAD4
SMARD1
SMCHD1
SMN1

SNAP25
SPEG
SPTBN4
SPTLC1
SQSTM1
SRPK3
SSPN
STIM1
SUCLA2
SURF1
SYN2
SYNE1
SYNE2
SYT1
SYT2
TANGO2
TAZ
TBX5
TCAP
TCF21
TGFB1
TIA1
TIMM50
TK2
TMEM126B
TMEM43
TMEM70
TMPO
TNNT1
TNNT3
TNPO3
TNXB
TOR1AIP1
TPI1
TPM2
TPM3
TRAPPC11
TRDN
TRDMT1
TRIM32

TRIP4
TTN
TUBB2B
TUBB3
TWIST1
TWIST2
TWNK
TYMP
UNC13a
UNC50
VAMP1
VCP
VMA21
XK
VBTB42
ZC4H2
ZMPSTE24

Supplemental table 2. Known pathogenic variants identified by exome sequencing.

ID<br>Diagnosis	Gene	Chr: position	Coding effect	c.DNA change<br>protein change	Ref
S.2<br>MPD5	*ADSSL1*	14:105207568	Het	NM_199165.2:c.910G>A<br>p.Asp304Asn	26506222
S.3<br>MPD5	*ADSSL1*	14:105207568	Hom	NM_199165.2:c.910G>A<br>p.Asp304Asn	32331917
S.16<br>CTX	*CYP27A1*	2:219678909	Hom	NM_000784.3:c1183C>T<br>p.Arg395Cys	2019602
S.5<br>BMD	*DMD*	X:31893255_<br>31986835	Hemi	NM_004006.2:c.6683_7342del<br>p.Glu2147_Lys2366del	2063877
F.8.1; F.9.1<br>LGMD1D	*DNAJB6*	7:157160096	Het	NM_058246.3:c.265T>A<br>p.Phe89Ile	22366786
F.2.1; F.2.2<br>CMS10	*DOK7*	4:3494837_<br>3494840	Hom	NM_173660.5:c.1124_1127dupTGCC<br>p.Ala378Serfs*30	16917026
S.23<br>CMT2D	*GARS*	7:30671914	Het	NM_002047.2:c.1955G>C<br>p.Gly652Ala	25168514
F.1.1; F.1.2<br>APBD	*GBE1*	3:81691938	Het	NM_000158.3:c.986A>C<br>p.Tyr329Ser	25665141
		3:81542964_<br>81542972	Het	NM_000158.3:c.2053-3358_2053-3350delinsTGTTTTTTACATTACAGGT<br>p.Tyr686Serfs*3	25665141
F.4.1; F.4.2<br>Amyloidosis	*GSN*	9:124073097	Het	NM_000177.4:c.640G>T<br>p.Asp214Tyr	1338910
F.6.1<br>Sandhoff disease	*HEXB*	5:74014629	Het	NM_000521.3:c.1250C>T<br>p.Pro417Leu	25736553
		5:73980812_<br>73993196	Het	NM_000521.3:c.1_786del<br>p.Met1_Leu223del	25736553
	SH3TC2	5:148406435	Het	NM_024577.3:c.2860C>T<br>p.Arg954Ter	25736553
F.3.1; F.3.2<br>LGMDR23	*LAMA2*	6:129371234	Het	NM_000426.3:c.283+1G>A<br>p.(?)	20207543
S.12<br>CMT2A	*MFN2*	1:12052717	Het	NM_014874.3:c.281G>A<br>p.Arg94Gln	15064763
F10.1<br>ALS8	*VAPB*	20:56993374	Het	NM_004738.4:c.166C>T<br>p.Pro56Ser	29560381

Supplemental figure 1

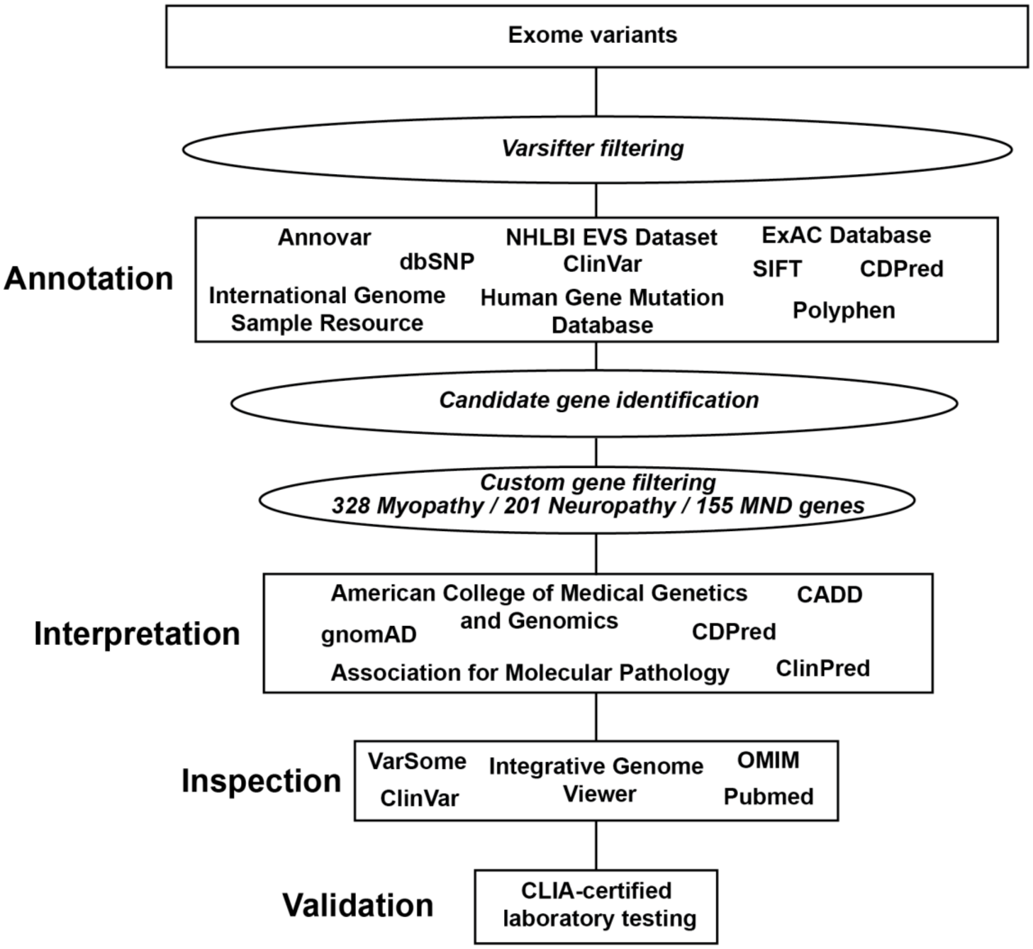

Supplemental figure 2

A.

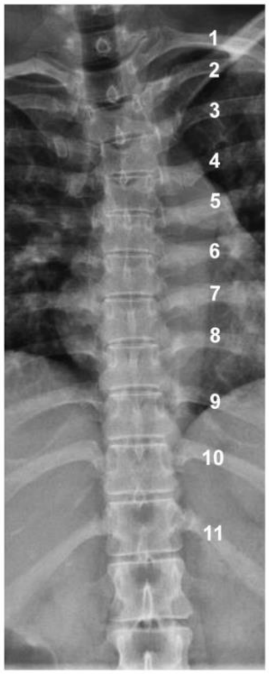

B.

Test	S.16	Control (mean ± SD)
1. Neuropsychological Assessment:		
Attention (Part A to D)	19 to 28	50 ± 10
Auditory comprehension	19	50 ± 10
Bill payment	19	50 ± 10
Naming	25	50 ± 10
2. Delis Kaplan Executive function:		
Letter fluency	5	10 ± 3
Category switching	6	10 ± 3
Number sequencing	5	10 ± 3
3. Motor speed and accuracy:		
Grooved pegboard (dominant)	16	50 ± 10
Grooved pegboard (non-dominant)	6	50 ± 10
4. Beery visual motor integration	50	100 ± 15
5. Adaptive behavior assessment	68	100 ± 15
6. Wechsler Adult Intelligence scale IV:		
Perceptual reasoning index	69	100 ± 15
Processing speed index	76	100 ± 15
Full scale IQ	73	100 ± 15
7. Beck depression inventory	29	

Supplemental figure 3

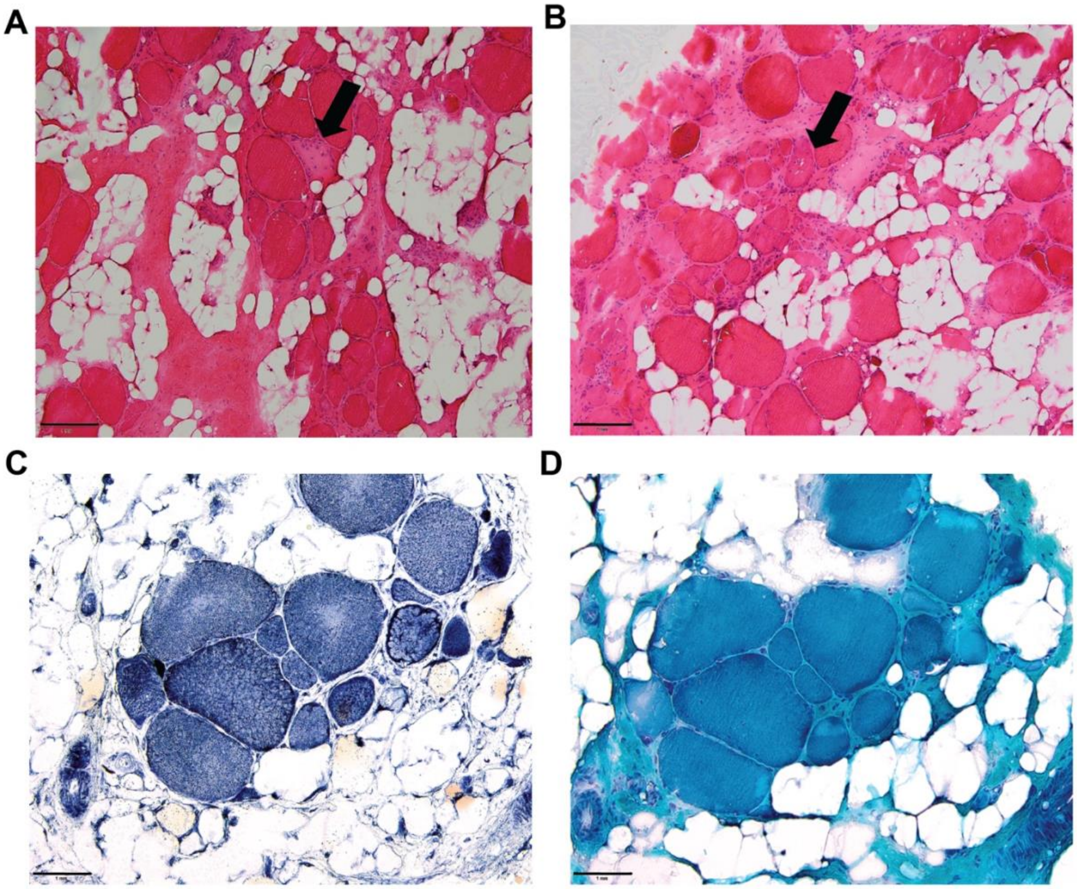

Supplemental figure 4

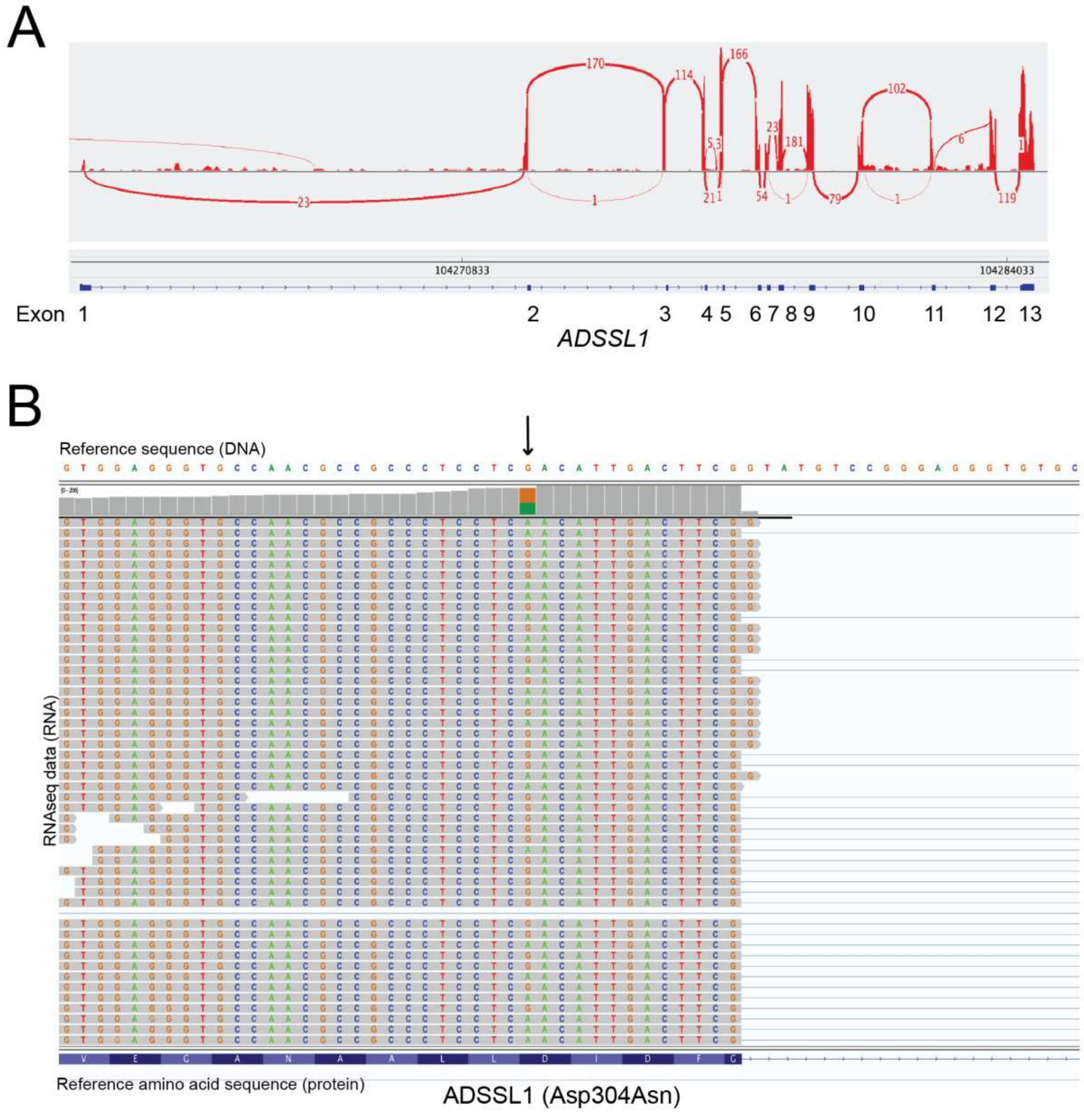

Supplement: Supplementary data [file jnnp-2020-325437supp001.pdf]
