# Supplementary material for: Improving the efficacy of exome sequencing at a quaternary care referral centre: novel mutations, clinical presentations and diagnostic challenges in rare neurogenetic diseases
Source: J Neurol Neurosurg Psychiatry. 2021 Jun 8;92(11):1186–96. doi: 10.1136/jnnp-2020-325437 (PMC8522445; doi:10.1136/jnnp-2020-325437)
Supplement: Supplementary data [file jnnp-2020-325437supp002.pdf]

Chr	LeftFlank	RightFlank	ref_allele	muttype	var_allele	type	Gene_name	Qual	loc_type	consequence	transcript	ref_aa	var_aa	aa_pos	CDPred_scor	dbID	Polyphen_ts	Polyphen_aa	Polyphen_an	CADD_Score	EXAC_POPM	ClinPred
chr9	107555092	107555094	T	SNP	G	nonsynonym	ABCA1	98	exonic	ABCA1:uc004bcl.3:exon42:c.5731A>C:p.I1911L,	uc004bcl.2	I	L	1911	-5	-	uc004bcl.2	I 1911 L	benign	19.77	0	0.78997761
chr1	76205703	76205705	G	SNP	T	nonsynonym	ACADM	100	exonic	ACADM:uc001dgw.4:exon7:c.508G>T:p.A170S,ACADM:u	uc001dgx.3	A	S	84	-6	-	uc009wbp.2	A 174 S	benign	21.1	0	0.1175106
chr1	76228446	76228454	AAAAAAA	INDEL	AAAAAAAA	frameshift_s	ACADM	25	exonic	ACADM:uc001dgw.4:exon12:c.1265_1271AAAAAAAA,AC	-	-	-	-	-30	-	NaN	NaN	NaN	NA	0	None
chr12	121176353	121176355	C	SNP	T	nonsynonym	ACADS	88	exonic	ACADS:uc010szl.1:exon7:c.802C>T:p.R268C,ACADS:uc00	uc001tza.3	R	C	272	-14	-	uc010szl.1	R 268 C	probably dan	20.8	1.00E-04	0.99944752
chr17	7123240	7123256	GGGCGTGCA	INDEL	GGGCGTGCA	nonframeshi	ACADVL	615	exonic	ACADVL:uc010vtq.2:exon1:c.76_90GGGCGTGCAGGACG	-	-	-	-	301	-	NaN	NaN	NaN	NA	0	None
chr17	7127323	7127325	T	SNP	C	nonsynonym	ACADVL	235	exonic	ACADVL:uc002gev.4:exon14:c.1370T>C:p.I457T,ACADVL:	uc010vtp.1	I	T	467	-8	-	uc010vtp.1	I 480 T	probably dan	8.703	0	0.98384964
chr14	105196229	105196231	A	SNP	C	nonsynonym	ADSSL1	453	exonic	ADSSL1:uc001ype.3:exon1:c.1A>C:p.M1L,	uc001ype.2	M	L	1	2	rs80097179(	uc001ype.2	M 1 L	benign	2.92	0	0.00880963
chr1	100336030	100336032	C	SNP	T	nonsynonym	AGL	189	exonic	AGL:uc001dsj.1:exon6:c.740C>T:p.A247V,AGL:uc001dsm	uc001dsn.1	A	V	230	0	-	uc001dsn.1	A 230 V	probably dan	33	0	0.9968124
chr1	100347124	100347126	T	SNP	C	nonsynonym	AGL	101	exonic	AGL:uc001dsj.1:exon17:c.2186T>C:p.I729T,AGL:uc001ds	uc001dsj.1	I	T	729	-1	-	uc001dsm.1	I 713 T	possibly dam	20.7	0	0.91919285
chr1	100353663	100353665	G	SNP	T	nonsynonym	AGL	86	exonic;splicir	AGL:uc001dsj.1:exon21:c.2812G>T:p.G938C,AGL:uc001d	uc001dsn.1	G	C	921	-3	-	uc001dsn.1	G 921 C	probably dan	24.4	0	0.99944466
chr9	139581758	139581773	CAGCAGCAG	INDEL	CAGCAGCAG	nonframeshi	AGPAT2	312	exonic	AGPAT2:uc004cij.1:exon1:c.38_51TGCTGCTGCTGCTGCT	-	-	-	-	301	-	NaN	NaN	NaN	NA	0	None
chr1	976124	976132	AAGAAGA	INDEL	AAGA	nonframeshi	AGRN	17	exonic	AGRN:uc001ack.2:exon4:c.592_598AAGA,		-	-	-	301	-	NaN	NaN	NaN	NA	0	None
chr21	45710527	45710529	G	SNP	C	nonsynonym	AIRE	174	exonic	AIRE:uc031rvv.1:exon1:c.52G>C:p.D18H,	uc031rvv.1	D	H	18	-1	rs2073610(G	NaN	NaN	NaN	3.087	0	None
chr21	45710555	45710560	CCCC	INDEL	CCCCC	frameshift_s	AIRE	219	exonic	AIRE:uc031rvv.1:exon1:c.80_83CCCCC,	-	-	-	-	-30	-	NaN	NaN	NaN	NA	0	None
chr21	45710616	45710618	G	SNP	C	nonsynonym	AIRE	374	exonic	AIRE:uc031rvv.1:exon1:c.141G>C:p.W47C,	uc031rvv.1	W	C	47	-2	-	NaN	NaN	NaN	4.515	0	None
chr21	45710794	45710807	GATGGATGC	INDEL	GA	frameshift_s	AIRE	58	exonic	AIRE:uc031rvv.1:exon1:c.319_330GA,AIRE:uc002zej.3:ex	-	-	-	-	-30	-	NaN	NaN	NaN	NA	0	None
chr21	45717583	45717585	C	SNP	T	nonsynonym	AIRE	129	exonic	AIRE:uc002zei.3:exon14:c.1612C>T:p.R538C,AIRE:uc031r	uc010gpr.2	R	C	341	-3	-	uc002zej.2	R 341 C	probably dan	19.65	0	0.99072027
chr9	101980916	101980918	A	SNP	G	nonsynonym	ALG2	150	exonic	ALG2:uc004azg.3:exon3:c.271T>C:p.F91L,ALG2:uc004azf	uc004azf.2	F	L	184	-1	-	uc004azg.2	F 91 L	probably dan	26.7	0	0.98062277
chr9	101984145	101984147	A	SNP	G	nonsynonym	ALG2	141	exonic	ALG2:uc004azf.3:exon1:c.31T>C:p.S11P,	uc004azf.2	S	P	11	-1	rs11545137(	uc004azf.2	S 11 P	benign	8.427	0	0.00882664
chr2	202609042	202609044	C	SNP	G	nonsynonym	ALS2	206	exonic	ALS2:uc002uyp.4:exon10:c.2108G>C:p.R703T,ALS2:uc00	uc002uyo.2	R	T	703	-6	-	uc002uyp.3	R 703 T	probably dan	28.9	0	0.90593332
chr2	202622162	202622164	C	SNP	G	nonsynonym	ALS2	83	exonic	ALS2:uc002uyp.4:exon5:c.1433G>C:p.G478A,ALS2:uc002	uc002uyq.2	G	A	478	0	-	uc002uyo.2	G 478 A	benign	8.487	0	0.12436493
chr5	34004692	34004694	T	SNP	C	nonsynonym	AMACR	80	exonic	AMACR:uc003jil.2:exon3:c.538A>G:p.I180V,AMACR:uc0	uc003jil.1	I	V	180	3	-	uc003jij.2	I 180 V	benign	11.23	0	0.18205971
chr11	22249067	22249069	G	SNP	A	nonsynonym	ANO5	117	exonic	ANO5:uc001mqi.2:exon7:c.584G>A:p.R195Q,ANO5:uc0	uc001mqi.2	R	Q	195	1	-	uc001mqi.2	R 195 Q	possibly dam	26.8	1.00E-04	0.93578249
chr11	22249075	22249077	C	SNP	G	nonsynonym	ANO5	115	exonic	ANO5:uc001mqi.2:exon7:c.592C>G:p.L198V,ANO5:uc00	uc001mqj.2	L	V	197	1	-	uc001mqi.2	L 198 V	possibly dam	18.74	0	0.83588487
chrX	66766356	66766409	GGCGGCGG	INDEL	GGCGGCGG	nonframeshi	AR	599	exonic	AR:uc022byk.1:exon1:c.1369_1420GGCGGCGGCGGCGG	-	-	-	-	301	-	NaN	NaN	NaN	NA	0	None
chr8	1851668	1851670	T	INDEL	TT	frameshift_s	ARHGEF10	172	exonic	ARHGEF10:uc003wpt.3:exon13:c.1501_1501delinsTT,	-	-	-	-	-30	-	NaN	NaN	NaN	NA	0	None
chr8	1871140	1871142	A	SNP	T	nonsynonym	ARHGEF10	63	exonic	ARHGEF10:uc003wps.3:exon18:c.2041A>T:p.M681L,ARH	uc003wpv.2	M	L	452	2	-	uc003wpr.2	M 719 L	benign	2.175	0	0.04620302
chr8	1872028	1872030	A	SNP	G	nonsynonym	ARHGEF10	189	exonic	ARHGEF10:uc003wps.3:exon20:c.2363A>G:p.K788R,ARH	uc003wpr.2	K	R	826	2	-	uc003wpv.2	K 851 R	probably dan	17.48	0	0.89922547
chr8	17942263	17942265	C	SNP	G	nonsynonym	ASAH1	117	exonic	ASAH1:uc003wyn.2:exon1:c.47G>C:p.R16P,ASAH1:uc003	uc003wyn.2	R	P	16	-2	-	uc003wyo.2	R 16 P	benign	6.784	0	0.12030354
chr11	63438835	63438837	C	SNP	T	nonsynonym	ATL3	138	exonic	ATL3:uc001nxl.1:exon2:c.128G>A:p.C43Y,	uc001nxl.1	C	Y	43	-2	rs2959880(C,	uc001nxl.1	C 43 Y	benign	0.007	0	0.01206867
chr11	108119734	108119736	A	SNP	G	nonsynonym	ATM	81	exonic	ATM:uc009yxr.1:exon10:c.1141A>G:p.S381G,ATM:uc001	uc001pkb.1	S	G	381	0	-	uc001pkb.1	S 381 G	benign	0.085	0	0.04148014
chr11	108175487	108175489	G	SNP	A	nonsynonym	ATM	56	exonic	ATM:uc009yxt.1:exon1:c.13G>A:p.A5T,	uc001pke.1	L	L	513	0	-	NaN	NaN	NaN	6.136	1.00E-04	None
chr6	16306671	16306673	C	SNP	A	nonsynonym	ATXN1	105	exonic	ATXN1:uc003nbt.3:exon9:c.2336G>T:p.S779I,ATXN1:uc0	uc003nbt.2	S	I	779	-2	-	uc003nbt.2	S 779 I	benign	14.35	0	0.51067108
chr6	16327201	16327203	C	SNP	T	nonsynonym	ATXN1	140	exonic	ATXN1:uc003nbt.3:exon8:c.1340G>A:p.G447E,ATXN1:uc	uc010jpi.2	G	E	447	-2	-	uc003nbt.2	G 447 E	probably dan	22.5	0	0.8253293
chr6	16327864	16327909	TGCTGCTGC	INDEL	TGCTGCTGC	nonframeshi	ATXN1	173	exonic	ATXN1:uc003nbt.3:exon8:c.634_677CAGCAGCAGCA	-	-	-	-	301	-	NaN	NaN	NaN	NA	0	None
chr6	16327896	16327898	C	SNP	A	nonsynonym	ATXN1	140	exonic	ATXN1:uc003nbt.3:exon8:c.645G>T:p.Q215H,ATXN1:uc0	uc010jpi.2	Q	H	215	0	-	uc003nbt.2	Q 215 H	unknown	1.752	0	0.00847288
chr6	16327902	16327904	C	SNP	A	nonsynonym	ATXN1	140	exonic	ATXN1:uc003nbt.3:exon8:c.639G>T:p.Q213H,ATXN1:uc0	uc010jpi.2	Q	H	213	0	-	uc003nbt.2	Q 213 H	unknown	4.787	0	0.00713906
chr6	16327903	16327921	TGCTGATGC	INDEL	TGCTGATGC	nonframeshi	ATXN1	470	exonic	ATXN1:uc003nbt.3:exon8:c.622_638CAGCAGCATCAGCA	-	-	-	-	301	-	NaN	NaN	NaN	NA	0	None
chr6	16327903	16327909	TGCTG	INDEL	TGCTGCTGA	nonframeshi	ATXN1	49	exonic	ATXN1:uc003nbt.3:exon8:c.634_638CAGCATCAGCAGCA,	-	-	-	-	301	-	NaN	NaN	NaN	NA	0	None
chr6	16327908	16327910	A	SNP	C	nonsynonym	ATXN1	353	exonic	ATXN1:uc003nbt.3:exon8:c.633T>G:p.H211Q,ATXN1:uc0	uc003nbt.2	H	Q	211	0	-	uc003nbt.2	H 211 Q	unknown	5.976	0	0.10019836
chr6	16327909	16327924	TGCTGATGC	INDEL	TGCTGATGC	nonframeshi	ATXN1	388	exonic	ATXN1:uc003nbt.3:exon8:c.619_632CAGCATCAGCA,ATX	-	-	-	-	301	-	NaN	NaN	NaN	NA	0	None
chr6	16327912	16327918	TGATG	INDEL	TGATGCTG	nonframeshi	ATXN1	491	exonic	ATXN1:uc003nbt.3:exon8:c.625_629CAGCATCA,ATXN1:u	-	-	-	-	301	-	NaN	NaN	NaN	NA	0	None
chr6	16327914	16327916	A	SNP	C	nonsynonym	ATXN1	190	exonic	ATXN1:uc003nbt.3:exon8:c.627T>G:p.H209Q,ATXN1:uc0	uc003nbt.2	H	Q	209	0	rs11969612(	uc003nbt.2	H 209 Q	benign	0.432	0	0.18909662
chr6	16327915	16327956	TGCTGCTGC	INDEL	TGCTGCTGC	nonframeshi	ATXN1	358	exonic	ATXN1:uc003nbt.3:exon8:c.587_626AGCAGCAGCAGCAG	-	-	-	-	301	-	NaN	NaN	NaN	NA	0	None
chr6	16327915	16327921	TGCTG	INDEL	TG	nonframeshi	ATXN1	474	exonic	ATXN1:uc003nbt.3:exon8:c.622_626CA,ATXN1:uc010jpi.	-	-	-	-	301	-	NaN	NaN	NaN	NA	0	None
chr22	46067965	46067967	C	SNP	T	nonsynonym	ATXN10	28	exonic	ATXN10:uc003bgm.2:exon1:c.23C>T:p.P8L,ATXN10:uc01	uc003bgm.1	P	L	8	-3	-	uc003bgm.1	P 8 L	possibly dam	12.67	1.00E-04	None
chr22	46125399	46125401	T	SNP	C	nonsynonym	ATXN10	135	exonic	ATXN10:uc003bgm.2:exon7:c.824T>C:p.I275T,ATXN10:u	uc003bgm.1	I	T	275	-1	-	uc003bgm.1	I 275 T	benign	8.304	1.00E-04	None
chr12	112036753	112036782	GCTGCTGCT	INDEL	GCTGCTGCT	nonframeshi	ATXN2	191	exonic	ATXN2:uc001tsj.3:exon1:c.538_565CAGCAGCAGCAGCA	-	-	-	-	301	-	NaN	NaN	NaN	NA	0	None
chr12	112036767	112036797	TGCTGCTGC	INDEL	TGCTGCTGC	nonframeshi	ATXN2	771	exonic	ATXN2:uc001tsj.3:exon1:c.523_551CAACAGCAGCAGCA,	-	-	-	-	301	-	NaN	NaN	NaN	NA	0	None
chr12	112036965	112036967	G	SNP	A	nonsynonym	ATXN2	118	exonic	ATXN2:uc001tsj.3:exon1:c.353C>T:p.P118L,	uc001tsj.2	P	L	118	-3	-	uc001tsj.2	P 118 L	benign	11.25	0	0.25400221
chr14	92537353	92537355	C	SNP	G	nonsynonym	ATXN3	201	exonic	ATXN3:uc021rzo.1:exon10:c.916G>C:p.G306R,ATXN3:uc	uc010auh.2	G	R	240	-2	rs12895357(	uc001yac.3	G 306 R	benign	9.58	0	0.6072396
chr14	92537353	92537354	''	INDEL	GCTGCTGCT	nonframeshi	ATXN3	139	exonic	ATXN3:uc021rzo.1:exon10:c.916_917insAGCAGCAGCAG	-	-	-	-	301	-	NaN	NaN	NaN	NA	0	None
chr14	92537354	92537379	CTGCTGCTG	INDEL	CTGCTGCTG	nonframeshi	ATXN3	307	exonic	ATXN3:uc021rzo.1:exon10:c.892_915CAGCAGCAGCAGC	-	-	-	-	301	-	NaN	NaN	NaN	NA	0	None
chr14	92537364	92537387	TGCTGCTGC	INDEL	TGCTGCTGC	nonframeshi	ATXN3	391	exonic	ATXN3:uc021rzo.1:exon10:c.884_905AGCAGCAACAGCA	-	-	-	-	301	-	NaN	NaN	NaN	NA	0	None
chr3	63898319	63898336	GCGGCGGCG	INDEL	GCGGCGGCG	nonframeshi	ATXN7	22	exonic	ATXN7:uc003dlw.4:exon3:c.46_61GCGGCGGCGGCGGCG	-	-	-	-	301	-	NaN	NaN	NaN	NA	0	None
chr3	63898360	63898393	GCAGCAGCA	INDEL	GCAGCAGCA	nonframeshi	ATXN7	750	exonic	ATXN7:uc003dlw.4:exon3:c.87_118GCAGCAGCAGCAGCA	-	-	-	-	301	-	NaN	NaN	NaN	NA	0	None
chr3	63898389	63898391	A	SNP	C	nonsynonym	ATXN7	35	exonic	ATXN7:uc003dlw.4:exon3:c.116A>C:p.Q39P,ATXN7:uc02	uc003dlw.3	Q	P	39	-1	-	uc010hnv.2	Q 39 P	unknown	6.467	0	0.00802094
chr3	63898390	63898393	GC	INDEL	GCAGCAGCC	nonframeshi	ATXN7	13	exonic	ATXN7:uc003dlw.4:exon3:c.117_118GCAGCAGCCGC,ATX	-	-	-	-	301	-	NaN	NaN	NaN	NA	0	None
chr3	63967899	63967901	A	SNP	G	nonsynonym	ATXN7	293	exonic	ATXN7:uc003dlw.4:exon7:c.791A>G:p.K264R,ATXN7:uc0	uc003dlv.2	K	R	264	2	rs1053338(A	uc011bfn.1	K 119 R	probably dan	16.9	0	0.02421619
chr12	58023948	58023950	T	SNP	C	nonsynonym	B4GALNT1	219	exonic	B4GALNT1:uc001spg.2:exon6:c.698A>G:p.N233S,B4GAL	uc010srv.1	N	S	233	1	-	uc001sph.2	N 233 S	possibly dam	11.25	0	0.35517764
chr9	95480096	95480098	C	SNP	T	nonsynonym	BICD2	75	exonic	BICD2:uc004asp.1:exon6:c.2240G>A:p.R747H,BICD2:uc0	uc004aso.1	R	H	747	0	-	uc004aso.1	R 747 H	probably dan	32	0	0.99854004
chr2	127827605	127827607	T	SNP	C	nonsynonym	BIN1	140	exonic	BIN1:uc010yzg.2:exon5:c.376A>G:p.M126V,BIN1:uc002t	uc010yzg.1	M	V	126	-5	-	uc002tob.1	M 126 V	benign	15.23	0	0.39668006
chr10	102747992	102747994	A	SNP	C	nonsynonym	C10ORF2	140	exonic	C10orf2:uc001ksf.2:exon1:c.26A>C:p.Y9S,C10orf2:uc001	uc001ksf.2	Y	S	9	-2	-	uc001ksf.2	Y 9 S	benign	3.456	0	0.1835385
chr10	102748011	102748013	A	SNP	C	nonsynonym	C10ORF2	140	exonic	C10orf2:uc001ksf.2:exon1:c.45A>C:p.L15F,C10orf2:uc001	uc001ksg.2	L	F	15	0	-	uc001ksf.2	L 15 F	probably dan	12.75	0	0.58502692
chr1	201031098	201031100	G	SNP	T	nonsynonym	CACNA1S	140	exonic	CACNA1S:uc001gvv.3:exon24:c.3026C>A:p.T1009K,	uc001gvv.2	T	K	1009	-2	-	uc001gvv.2	T 1009 K	probably dan	33	0	0.99620265
chr1	201035375	201035377	T	SNP	C	nonsynonym	CACNA1S	122	exonic	CACNA1S:uc001gvv.3:exon21:c.2726A>G:p.N909S,	uc001gvv.2	N	S	909	2	-	uc001gvv.2	N 909 S	possibly dam	18.9	0	0.53944581
chr1	201052334	201052336	C	SNP	T	nonsynonym	CACNA1S	136	exonic	CACNA1S:uc001gvv.3:exon10:c.1348G>A:p.A450T,	uc001gvv.2	A	T	450	0	-	uc001gvv.2	A 450 T	probably dan	29.6	1.00E-04	0.57260972
chr15	42697152	42697154	C	SNP	T	splicing	CAPN3	115	splicing	-	-	-	-	-	-30	-	NaN	NaN	NaN	4.328	0	None
chr20	44803491	44803493	A	SNP	C	nonsynonym	CDH22	101	exonic	CDH22:uc010ghk.2:exon12:c.2140T>G:p.S714A,	uc010ghk.1	S	A	714	2	-	uc002xrm.2	S 714 A	benign	5.943	0	0.0178779
chr11	2906196	2906236	CCGCGACCG	INDEL	CCGCGACCG	nonframeshi	CDKN1C	166	exonic	CDKN1C:uc009ydr.3:exon2:c.452_490CGGCTCCGGTCGC	-	-	-	-	301	-	NaN	NaN	NaN	NA	0	None
chr10	50822259	50822261	A	SNP	G	nonsynonym	CHAT	31	exonic	CHAT:uc001jhz.2:exon1:c.25A>G:p.R9G,	uc001jhz.2	R	G	9	-2	-	uc001jhz.2	R 9 G	benign	11.23	0	0.02532788
chr10	50822391	50822393	G	SNP	A	nonsynonym	CHAT	100	exonic	CHAT:uc001jhz.2:exon1:c.157G>A:p.G53R,	uc001jhz.2	G	R	53	-2	-	uc001jhz.2	G 53 R	benign	8.771	0	0.03655029
chr8	61693942	61693952	AAAGCAAAA	INDEL	AAAGCAAAA	nonframeshi	CHD7	188	exonic	CHD7:uc003xue.3:exon3:c.2050_2058AAAGCAAAAGCAA	-	-	-	-	301	-	NaN	NaN	NaN	NA	0	None
chr8	61741305	61741307	C	SNP	T	nonsynonym	CHD7	123	exonic	CHD7:uc003xue.3:exon14:c.3463C>T:p.R1155C,	uc003xue.2	R	C	1155	-7	-	uc003xue.2	R 1155 C	probably dan	28.9	1.00E-04	0.89888185
chr8	61761698	61761700	G	SNP	C	nonsynonym	CHD7	91	exonic	CHD7:uc003xue.3:exon25:c.5390G>C:p.G1797A,	uc003xue.2	G	A	1797	0	-	uc003xue.2	G 1797 A	probably dan	19.04	0	0.96501952
chr2	175676256	175676258	T	SNP	C	nonsynonym	CHN1	77	exonic	CHN1:uc002ujj.3:exon9:c.271A>G:p.K91E,CHN1:uc010ze	uc002ujj.2	K	E	91	-6	-	uc010zeq.1	K 290 E	probably dan	33	0	0.95453489
chr2	233393247	233393249	G	SNP	T	nonsynonym	CHRND	140	exonic	CHRND:uc002vsw.4:exon5:c.391G>T:p.V131L,CHRND:uc	uc010zmg.1	V	L	116	-1	-	uc002vsw.2	V 131 L	possibly dam	19.03	0	0.88519377
chr2	233393681	233393683	G	SNP	A	splicing	CHRND	59	splicing	uc031rru.1:exon6:c.316+1G>A,uc002vsw.4:exon6:c.619+	-	-	-	-	-30	-	NaN	NaN	NaN	14.94	0	None
chr17	4806321	4806323	C	SNP	T	nonsynonym	CHRNE	336	exonic	CHRNE:uc002fzk.1:exon1:c.37G>A:p.G13R,	uc002fzk.1	G	R	13	-2	-	uc002fzk.1	G 13 R	benign	13.28	1.00E-04	0.13646635
chr3	9908961	9908963	G	SNP	A	nonsynonym	CIDEC	98	exonic	CIDEC:uc010hcp.3:exon3:c.226C>T:p.P76S,	uc010hcp.2	P	S	76	-1	-	uc010hcp.2	P 76 S	benign	8.355	0	0.07031593
chr7	143013421	143013423	T	SNP	G	nonsynonym	CLCN1	38	exonic	CLCN1:uc003wcr.1:exon1:c.117T>G:p.N39K,	uc003wcr.1	N	K	39	0	-	uc003wcr.1	N 39 K	benign	12.55	0	0.09913196
chr16	28493828	28493830	T	SNP	C	nonsynonym	CLN3	273	exonic	CLN3:uc002dps.1:exon7:c.494A>G:p.Y165C,CLN3:uc010v	uc002dpw.1	Y	C	139	-11	-	uc002dps.1	Y 238 C	probably dan	17.13	1.00E-04	0.99956197
chr16	28500680	28500682	G	SNP	A	nonsynonym	CLN3	107	exonic	CLN3:uc010bye.1:exon3:c.152C>T:p.S51F,CLN3:uc002dp	uc010bye.1	S	F	51	-3	-	uc002dpq.1	S 51 F	probably dan	24.1	0	0.94149125
chr16	28502843	28502845	C	SNP	T	stopgain_SN	CLN3	140	exonic	CLN3:uc002dps.1:exon2:c.89G>A:p.W30X,	uc002dps.1	W	*	30	-30	-	NaN	NaN	NaN	9.15	0	None
chr11	57426925	57426928	GG	INDEL	G	splicing	CLP1	179	splicing	-	-	-	-	-	-30	-	NaN	NaN	NaN	NA	0	None
chr3	1363439	1363441	G	SNP	T	stopgain_SN	CNTN6	92	exonic	CNTN6:uc003bpa.3:exon8:c.868G>T:p.E290X,CNTN6:uc0	uc003boz.2	E	*	290	-30	-	NaN	NaN	NaN	44	0	None
chr6	75855167	75855169	G	SNP	A	nonsynonym	COL12A1	80	exonic	COL12A1:uc021zbw.1:exon9:c.1072C>T:p.R358C,COL12A	uc003pht.2	R	C	358	-6	-	uc003phs.2	R 1522 C	probably dan	14.49	1.00E-04	0.94988334
chr6	75861863	75861865	C	SNP	G	nonsynonym	COL12A1	144	exonic	COL12A1:uc021zbw.1:exon3:c.326G>C:p.G109A,COL12A	uc003pht.2	G	A	109	-7	-	uc003pht.2	G 109 A	probably dan	22.5	1.00E-04	0.94561964
chr10	71662527	71662529	C	SNP	G	nonsynonym	COL13A1	129	exonic	COL13A1:uc021prz.1:exon14:c.739C>G:p.Q247E,COL13A	uc001jqi.1	Q	E	269	2	-	uc001jpt.1	Q 247 E	probably dan	18.95	0	0.7466169
chr21	47410908	47410910	C	SNP	T	nonsynonym	COL6A1	105	exonic	COL6A1:uc002zhu.1:exon15:c.1073C>T:p.P358L,	uc002zhu.1	P	L	358	-11	-	uc002zhu.1	P 358 L	probably dan	16.66	0	0.98278481
chr21	47412310	47412312	G	SNP	A	nonsynonym	COL6A1	140	exonic;splicir	COL6A1:uc002zhu.1:exon18:c.1271G>A:p.R424Q,	uc002zhu.1	R	Q	424	1	-	uc002zhu.1	R 424 Q	probably dan	17.4	1.00E-04	0.41706696
chr21	47532185	47532187	G	SNP	A	nonsynonym	COL6A2	140	exonic	COL6A2:uc002zhz.1:exon3:c.409G>A:p.A137T,COL6A2:uc	uc002zhz.1	A	T	137	-8	-	uc002zhz.1	A 137 T	probably dan	18.55	0	0.9658531
chr21	47551953	47551955	C	SNP	T	nonsynonym	COL6A2	174	exonic	COL6A2:uc002zia.1:exon28:c.2548C>T:p.H850Y,	uc002zia.1	H	Y	850	-3	-	uc002zia.1	H 850 Y	benign	10.78	1.00E-04	0.14369014
chr2	238244863	238244878	GCAGCAGCA	INDEL	GCAGCAGCA	nonframeshi	COL6A3	345	exonic	COL6A3:uc002vwo.2:exon39:c.8248_8261GCTGCTGCTG	-	-	-	-	301	-	NaN	NaN	NaN	NA	0	None
chr2	238261208	238261210	C	SNP	G	nonsynonym	COL6A3	62	exonic	COL6A3:uc002vwp.1:exon3:c.172G>C:p.G58R,COL6A3:uc	uc002vwp.1	G	R	58	-2	-	uc010znj.1	G 1630 R	probably dan	13.74	0	0.9857502
chr2	238263603	238263605	G	INDEL	''	frameshift_d	COL6A3	0	exonic	COL6A3:uc002vwp.1:exon1:c.28delC:p.Q10fs,	-	-	-	-	-30	-	NaN	NaN	NaN	NA	0	None
chr2	238280405	238280411	CTTCT	INDEL	CT	nonframeshi	COL6A3	71	exonic	COL6A3:uc002vwo.2:exon8:c.3632_3636AG,COL6A3:uc0	-	-	-	-	301	-	NaN	NaN	NaN	NA	0	None
chr20	61455814	61455829	CCAGGTCCC	INDEL	CCAGG	nonframeshi	COL9A3	316	exonic	COL9A3:uc002ydm.3:exon11:c.538_551CCAGG,		-	-	-	301	-	NaN	NaN	NaN	NA	0	None
chr3	15520887	15520889	C	SNP	T	nonsynonym	COLQ	151	exonic;splicir	COLQ:uc003bzx.3:exon4:c.323G>A:p.G108D,COLQ:uc010	uc010heo.2	G	D	74	-1	-	uc003bzz.2	G 108 D	probably dan	10.2	0	0.98729521
chr17	13977740	13977742	A	SNP	G	nonsynonym	COX10	93	exonic	COX10:uc002gof.4:exon2:c.145A>G:p.T49A,	uc002gof.3	T	A	49	0	-	uc002gof.3	T 49 A	possibly dam	17.72	0	0.96179175
chr10	101486814	101486816	G	SNP	C	nonsynonym	COX15	140	exonic	COX15:uc001kqc.4:exon4:c.492C>G:p.I164M,COX15:uc0	uc001kqb.3	I	M	164	-5	-	uc001kqb.3	I 164 M	benign	14.41	0	0.38121095
chr12	120876261	120876263	C	SNP	T	nonsynonym	COX6A1	140	exonic	COX6A1:uc001tyf.2:exon2:c.184C>T:p.H62Y,	uc001tyf.1	H	Y	62	-7	-	uc001tyf.1	H 62 Y	benign	15.36	0	0.19621912
chr1	53678968	53678970	G	SNP	A	nonsynonym	CPT2	82	exonic	CPT2:uc001cvb.4:exon5:c.1679G>A:p.R560Q,	uc001cvb.3	R	Q	560	-5	-	uc001cvb.3	R 560 Q	benign	19.31	1.00E-04	0.120353
chr2	36726453	36726455	G	SNP	A	nonsynonym	CRIM1	89	exonic	CRIM1:uc002rpd.3:exon8:c.1465G>A:p.D489N,	uc002rpd.2	D	N	489	1	-	uc002rpd.2	D 489 N	probably dan	27.9	1.00E-04	0.32586071
chr18	77475187	77475209	GGAGGAGG	INDEL	GGAGGAGG	nonframeshi	CTDP1	211	exonic	CTDP1:uc010drd.2:exon8:c.1728_1748GGAGGAGGAGG	-	-	-	-	301	-	NaN	NaN	NaN	NA	0	None
chr17	3540606	3540611	AGAG	INDEL	AG	splicing	CTNS	44	splicing	-	-	-	-	-	-30	-	NaN	NaN	NaN	NA	0	None
chr17	3560024	3560033	TCTTCTTC	INDEL	TCTTC	nonframeshi	CTNS	88	exonic	CTNS:uc010ckj.3:exon9:c.617_624TCTTC,CTNS:uc002fwa	-	-	-	-	301	-	NaN	NaN	NaN	NA	0	None
chr20	44520237	44520264	CTGCTGCTG	INDEL	CTGCTGCTG	nonframeshi	CTSA	940	exonic	CTSA:uc002xqj.4:exon2:c.31_56CTGCTGCTGCTGCTG	-	-	-	-	301	-	NaN	NaN	NaN	NA	0	None
chr2	219647107	219647109	T	SNP	G	nonsynonym	CYP27A1	223	exonic	CYP27A1:uc002viz.4:exon1:c.203T>G:p.F68C,	uc002viz.3	F	C	68	-2	-	uc002viz.3	F 68 C	probably dan	10.66	0	0.42887446
chr2	219677021	219677023	C	SNP	T	nonsynonym	CYP27A1	104	exonic	CYP27A1:uc002viz.4:exon3:c.524C>T:p.T175M,	uc002viz.3	T	M	175	-3	-	uc002viz.3	T 175 M	probably dan	13.7	0	0.02825929
chr22	42523885	42523887	C	SNP	T	nonsynonym	CYP2D6	140	exonic	CYP2D6:uc010gyu.3:exon4:c.25G>A:p.A9T,CYP2D6:uc003	uc010gyu.2	A	T	9	1	-	uc003bcf.2	A 264 T	benign	8.197	0	None
chr22	42524175	42524181	CTTCT	INDEL	CT	nonframeshi	CYP2D6	105	exonic	CYP2D6:uc003bce.3:exon5:c.839_843AG,CYP2D6:uc003b	-	-	-	-	301	-	NaN	NaN	NaN	NA	0	None
chr22	42524217	42524231	GGGCTGGG	INDEL	GGGCTGGG	frameshift_s	CYP2D6	165	exonic	CYP2D6:uc003bce.3:exon5:c.789_801CCCAGCCCAGCCCA	-	-	-	-	-30	-	NaN	NaN	NaN	NA	0	None
chr22	42524243	42524245	T	INDEL	''	frameshift_d	CYP2D6	0	exonic	CYP2D6:uc003bce.3:exon5:c.775delA:p.R259fs,CYP2D6:u	-	-	-	-	-30	-	NaN	NaN	NaN	NA	0	None
chr22	42524934	42524936	G	SNP	T	nonsynonym	CYP2D6	140	exonic	CYP2D6:uc003bce.3:exon4:c.517C>A:p.R173S,CYP2D6:uc	uc003bcf.2	R	S	122	-3	-	uc003bcf.2	R 122 S	benign	4.614	0	None
chr22	42525038	42525040	G	SNP	T	nonsynonym	CYP2D6	140	exonic	CYP2D6:uc003bce.3:exon3:c.501C>A:p.H167Q,	uc003bce.2	H	Q	167	5	-	uc003bce.2	H 167 Q	benign	0.021	0	None
chr2	74590752	74590754	C	SNP	A	stopgain_SN	DCTN1	333	exonic	DCTN1:uc002skv.3:exon22:c.2797G>T:p.E933X,DCTN1:uc	uc002skw.1	E	*	1043	-30	-	NaN	NaN	NaN	43	0	None
chr14	53619480	53619495	GCCGCCGCC	INDEL	GCCGCCGCC	nonframeshi	DDHD1	650	exonic	DDHD1:uc001xaj.3:exon1:c.323_336GCGGCGGCGGCGG	-	-	-	-	301	-	NaN	NaN	NaN	NA	0	None
chr1	231858295	231858297	T	SNP	C	nonsynonym	DISC1	167	exonic	DISC1:uc001hvc.3:exon4:c.1129T>C:p.C377R,DISC1:uc01	uc001hvc.3	C	R	377	-3	-	uc001hvc.3	C 377 R	probably dan	13.02	0	None
chr1	231990703	231990705	C	SNP	T	nonsynonym	DISC1	32	exonic	DISC1:uc010pww.2:exon10:c.2201C>T:p.P734L,	uc010pww.1	P	L	734	-3	-	uc010pww.1	P 734 L	benign	0.058	0	0.02646512
chr1	231990708	231990710	T	SNP	G	nonsynonym	DISC1	33	exonic	DISC1:uc010pww.2:exon10:c.2206T>G:p.S736A,	uc010pww.1	S	A	736	1	-	uc010pww.1	S 736 A	benign	0.945	0	0.13552765
chrX	32486772	32486774	T	SNP	A	nonsynonym	DMD	347	exonic	DMD:uc004dcy.1:exon23:c.2992A>T:p.T998S,DMD:uc004	uc004dcy.1	T	S	998	1	-	uc004dda.1	T 1002 S	benign	14.23	0	None
chrX	32563374	32563376	G	SNP	A	nonsynonym	DMD	631	exonic	DMD:uc004dcy.1:exon17:c.2057C>T:p.T686I,DMD:uc004	uc004ddf.2	T	I	682	-1	-	uc004dcy.1	T 686 I	probably dan	13.21	0	None
chr7	157159249	157159251	G	SNP	A	nonsynonym	DNAJB6	130	exonic	DNAJB6:uc003wnl.3:exon4:c.191G>A:p.G64E,DNAJB6:uc	uc011kvz.1	G	E	77	-8	-	uc011kvy.1	G 28 E	probably dan	15.81	0	0.92969632
chr7	157160095	157160097	T	SNP	A	nonsynonym	DNAJB6	131	exonic	DNAJB6:uc003wnl.3:exon5:c.226T>A:p.F76I,DNAJB6:uc0	uc010lqt.2	F	I	89	-7	-	uc003wnj.2	F 89 I	benign	16.03	0	0.96246564
chr13	96439340	96439353	AAGAAAAGA	INDEL	AAGAAAA	frameshift_s	DNAJC3	78	exonic	DNAJC3:uc001vmq.3:exon11:c.1289_1300AAGAAAA,	-	-	-	-	-30	-	NaN	NaN	NaN	NA	0	None
chr19	10906833	10906835	A	SNP	G	nonsynonym	DNM2	127	exonic	DNM2:uc002mpu.2:exon10:c.1294A>G:p.I432V,DNM2:u	uc002mpt.1	I	V	432	-3	-	uc002mpt.1	I 432 V	benign	7.204	0	0.53790151
chr20	31395671	31395673	T	SNP	C	nonsynonym	DNMT3B	140	exonic	DNMT3B:uc002wye.3:exon20:c.2276T>C:p.L759P,DNMT	uc002wyd.2	L	P	822	-8	-	uc010ztz.1	L 717 P	probably dan	26.7	0	0.99824476
chr7	154172164	154172166	C	SNP	A	nonsynonym	DPP6	88	exonic	DPP6:uc010lqh.1:exon3:c.314C>A:p.A105D,	uc010lqh.1	A	D	105	-2	-	NaN	NaN	NaN	7.829	0	None
chr7	154672646	154672648	G	SNP	A	nonsynonym	DPP6	232	exonic	DPP6:uc003wlm.3:exon21:c.1942G>A:p.G648R,DPP6:uc	uc003wli.2	G	R	646	-12	-	uc003wli.2	G 646 R	probably dan	17.25	0	0.99907792
chr18	32438352	32438354	G	SNP	A	nonsynonym	DTNA	29	exonic	DTNA:uc002kxz.2:exon14:c.1376G>A:p.R459Q,DTNA:uc	uc002kyb.3	R	Q	516	1	-	uc010xbx.1	R 269 Q	probably dan	32	0	0.98654002
chr2	71801334	71801350	AGGCGGAG	INDEL	AGGCGGAG	nonframeshi	DYSF	233	exonic	DYSF:uc010fee.3:exon30:c.3182_3196AGGCGGAGGCGG	-	-	-	-	301	-	NaN	NaN	NaN	NA	0	None
chr2	71894606	71894608	C	SNP	T	nonsynonym	DYSF	225	exonic	DYSF:uc010fee.3:exon48:c.5365C>T:p.R1789W,DYSF:uc	uc002sie.2	R	W	1768	-3	-	uc010fem.2	R 1790 W	probably dan	21.3	1.00E-04	None
chr2	233348877	233348879	A	SNP	G	nonsynonym	ECEL1	58	exonic	ECEL1:uc010fya.1:exon6:c.1240T>C:p.S414P,ECEL1:uc01	uc010fya.1	S	P	414	-8	-	uc002vsv.2	S 414 P	probably dan	15.04	0	0.79447651
chr17	4858468	4858470	A	SNP	G	nonsynonym	ENO3	120	exonic	ENO3:uc010vsr.2:exon8:c.265A>G:p.M89V,ENO3:uc010	uc010vsr.1	M	V	89	-4	-	uc002gab.3	M 182 V	benign	15.91	0	0.97007811
chr2	222307676	222307678	C	SNP	T	nonsynonym	EPHA4	147	exonic	EPHA4:uc002vmr.2:exon11:c.1946G>A:p.C649Y,EPHA4:u	uc002vmq.2	C	Y	649	-13	-	uc002vmq.2	C 649 Y	benign	1.058	0	0.69357723
chr10	50678316	50678318	C	SNP	T	nonsynonym	ERCC6	550	exonic	ERCC6:uc010qgr.3:exon12:c.1799G>A:p.R600H,ERCC6:uc	uc001jhr.3	R	H	598	0	rs4253211(C,	uc001jhs.3	R 1230 H	benign	13.63	1.00E-04	0.21551895
chr4	159618822	159618824	G	SNP	A	nonsynonym	ETFDH	131	exonic	ETFDH:uc011cjg.2:exon7:c.803G>A:p.G268D,ETFDH:uc0	uc010iqs.2	G	D	237	-3	-	uc011cjg.1	G 268 D	benign	16.98	1.00E-04	0.77118158
chr4	159627959	159627961	C	SNP	G	nonsynonym	ETFDH	81	exonic	ETFDH:uc011cjg.2:exon11:c.1507C>G:p.L503V,ETFDH:uc	uc011cjg.1	L	V	503	-3	-	uc003iqb.2	L 550 V	possibly dam	14.82	0	0.95966232
chr5	147785931	147785933	T	SNP	G	nonsynonym	FBXO38	47	exonic	FBXO38:uc031slt.1:exon6:c.843T>G:p.H281Q,FBXO38:uc	uc003lpg.1	H	Q	281	0	-	uc003lpg.1	H 281 Q	probably dan	19.8	0	0.99619031
chr5	147807361	147807371	TGGGAGTG	INDEL	TGG	nonframeshi	FBXO38	211	exonic	FBXO38:uc031slt.1:exon14:c.2505_2513TGG,	-	-	-	-	301	-	NaN	NaN	NaN	NA	0	None
chr12	32655271	32655273	G	SNP	C	splicing	FGD4	80	splicing	-	-	-	-	-	-30	rs77113168(	NaN	NaN	NaN	13.4	0	None
chr6	110098253	110098255	C	SNP	A	nonsynonym	FIG4	62	exonic	FIG4:uc003ptt.2:exon16:c.1880C>A:p.T627K,FIG4:uc011	uc003ptt.2	T	K	627	-1	-	uc003ptt.2	T 627 K	benign	3.824	1.00E-04	0.07733368
chr7	30058726	30058732	GGGGG	INDEL	GGGGGG	frameshift_s	FKBP14	44	exonic	FKBP14:uc003tal.2:exon3:c.358_362CCCCCC,	-	-	-	-	-30	-	NaN	NaN	NaN	NA	0	None
chr9	108397407	108397409	G	SNP	A	nonsynonym	FKTN	224	exonic	FKTN:uc004bcr.3:exon11:c.1249G>A:p.E417K,FKTN:uc01	uc011lvx.1	E	K	417	1	-	uc011lvx.1	E 417 K	benign	13.96	1.00E-04	0.10206618

Chr	LeftFlank	RightFlank	ref_allele	muttype	var_allele	type	Gene_name	Qual	loc_type	consequence	transcript	ref_aa	var_aa	aa_pos	CDPred_scor	dbID	Polyphen_ts	Polyphen_aa	Polyphen_an	CADD_Score	EXAC_POPM	ClinPred
chr1	154965149	154965153	GTG	INDEL	G	splicing	FLAD1	52	splicing	-	-	-	-	-	-30	-	NaN	NaN	NaN	NA	0	None
chr7	128484191	128484193	G	SNP	A	nonsynonym	FLNC	112	exonic	FLNC:uc003voa.4:exon20:c.3064G>A:p.E1022K,FLNC:uc0	uc003voa.3	E	K	1022	3	-	uc003vnz.3	E 1022 K	benign	16.76	0	0.83973229
chr11	63884588	63884590	C	SNP	T	nonsynonym	FLRT1	140	exonic	FLRT1:uc021qks.1:exon1:c.850C>T:p.H284Y,FLRT1:uc001	uc001nyi.1	H	Y	284	-8	-	uc001nyi.1	H 256 Y	benign	7.686	0	0.71530163
chr11	63884901	63884903	C	SNP	T	nonsynonym	FLRT1	140	exonic	FLRT1:uc021qks.1:exon1:c.1163C>T:p.A388V,FLRT1:uc00	uc001nyi.1	A	V	388	0	-	uc001nyi.1	A 360 V	benign	0.006	0	0.03284728
chrX	147011477	147011479	A	SNP	G	nonsynonym	FMR1	307	exonic	FMR1:uc022cgc.1:exon6:c.431A>G:p.E144G,FMR1:uc00	uc004fcl.3	E	G	5	-2	-	uc011mwz.1	E 144 G	possibly dam	17.38	0	None
chr17	78086700	78086702	G	SNP	A	nonsynonym	GAA	114	exonic	GAA:uc002jxq.3:exon14:c.1915G>A:p.V639M,GAA:uc002	uc002jxp.2	V	M	639	4	-	uc002jxo.2	V 639 M	possibly dam	19.67	0	0.95218599
chr7	30671913	30671915	G	SNP	C	nonsynonym	GARS	99	exonic	GARS:uc003tbm.3:exon16:c.1955G>C:p.G652A,	uc003tbm.2	G	A	652	-7	-	uc003tbm.2	G 652 A	benign	21.3	0	0.96315676
chr9	35740888	35740890	C	SNP	T	nonsynonym	GBA2	140	exonic	GBA2:uc011lpd.2:exon6:c.977G>A:p.R326Q,GBA2:uc003	uc003zxy.1	R	Q	33	1	-	uc003zxw.2	R 320 Q	benign	1.053	1.00E-04	0.0219721
chr3	81699025	81699027	G	SNP	A	nonsynonym	GBE1	117	exonic	GBE1:uc021xax.1:exon4:c.353C>T:p.P118L,GBE1:uc021x	uc003dqg.2	P	L	159	-14	-	uc003dqg.2	P 159 L	probably dam	29.6	0	0.9939785
chr2	69554079	69554081	A	SNP	G	nonsynonym	GFPT1	170	exonic	GFPT1:uc002sfi.2:exon19:c.2021T>C:p.L674P,GFPT1:uc0	uc002sfh.2	L	P	656	-10	-	uc002sfh.2	L 656 P	probably dam	23.5	0	None
chr9	124044745	124044747	C	SNP	T	nonsynonym	GSN	179	exonic	GSN:uc010mvu.2:exon3:c.17C>T:p.T6M,	uc010mvu.2	T	M	6	-1	-	NaN	NaN	NaN	5.155	0	None
chr9	124045670	124045672	G	SNP	A	splicing	GSN	35	splicing	-	-	-	-	-	-30	-	NaN	NaN	NaN	0.206	0	None
chr9	124073096	124073098	G	SNP	T	nonsynonym	GSN	172	exonic	GSN:uc010mvt.1:exon6:c.487G>T:p.D163Y,GSN:uc004bl	uc010mvt.1	D	Y	163	-10	-	uc011lyh.1	D 171 Y	probably dam	22.2	0	0.99975592
chr3	148714546	148714548	G	SNP	C	nonsynonym	GYG1	83	exonic	GYG1:uc003ewn.3:exon4:c.337G>C:p.D113H,GYG1:uc00	uc003ewo.2	D	H	113	-4	-	uc003ewp.2	D 113 H	probably dam	19.01	0	0.94648731
chr2	26477125	26477130	ACTA	INDEL	ACTACTA	nonframeshi	HADHB	690	exonic	HADHB:uc002rgz.3:exon2:c.4_7ACTACTA,HADHB:uc010y	-	-	-	-	301	-	NaN	NaN	NaN	NA	0	None
chr6	26093021	26093023	T	SNP	G	nonsynonym	HFE	153	exonic	HFE:uc003nge.1:exon2:c.186T>G:p.D62E,HFE:uc003ngb.	uc003nfz.1	D	E	154	-5	-	uc003ngc.1	D 150 E	probably dam	12.72	0	0.87574619
chr6	26093408	26093410	A	SNP	T	nonsynonym	HFE	110	exonic	HFE:uc003nge.1:exon3:c.415A>T:p.I139F,HFE:uc003ngb.	uc003nfx.1	I	F	319	0	-	uc003nge.1	I 139 F	possibly dam	16.86	0	0.28593346
chr10	71060523	71060525	G	SNP	A	splicing	HK1	140	splicing	-	-	-	-	-	-30	rs57118523	NaN	NaN	NaN	7.5	0	None
chr2	176981660	176981662	A	SNP	G	nonsynonym	HOXD10	99	exonic	HOXD10:uc002ukj.3:exon1:c.100A>G:p.M34V,	uc002ukj.2	M	V	34	1	-	uc002ukj.2	M 34 V	benign	5.328	0	0.75605494
chr2	176983958	176983985	GGTCTGAGG	INDEL	GGTCTGAGG	frameshift_s	HOXD10	239	exonic	HOXD10:uc002ukj.3:exon2:c.1023_1048GGTCTGAGGCC	-	-	-	-	-30	-	NaN	NaN	NaN	NA	0	None
chr1	22181218	22181220	G	SNP	A	nonsynonym	HSPG2	123	exonic	HSPG2:uc001bfj.3:exon49:c.6173C>T:p.S2058L,HSPG2:u	uc001bfj.2	S	L	2058	-5	-	uc001bfj.2	S 2058 L	probably dam	14.77	1.00E-04	0.68937945
chr1	22206682	22206684	C	SNP	T	nonsynonym	HSPG2	140	exonic	HSPG2:uc001bfj.3:exon17:c.2260G>A:p.G754S,HSPG2:u	uc009vqd.2	G	S	755	0	-	uc001bfj.2	G 754 S	benign	12.44	0	0.10509239
chr7	112102104	112102106	A	SNP	G	nonsynonym	IFRD1	123	exonic	IFRD1:uc003vgh.3:exon8:c.668A>G:p.Y223C,IFRD1:uc003	uc003vgj.2	Y	C	223	-9	-	uc003vgh.2	Y 223 C	benign	13.59	0	0.91550446
chr7	112107982	112107985	GG	INDEL	''	frameshift_d	IFRD1	0	exonic	IFRD1:uc003vgk.3:exon1:c.5_6del:p.2_2del,	-	-	-	-	-30	-	NaN	NaN	NaN	NA	0	None
chr7	112107984	112107986	T	SNP	A	nonsynonym	IFRD1	85	exonic	IFRD1:uc003vgk.3:exon1:c.7T>A:p.S3T,	uc003vgk.2	S	T	3	1	rs116076119	NaN	NaN	NaN	6.361	0	None
chr7	112107985	112107987	C	SNP	A	stopgain_SN	IFRD1	80	exonic	IFRD1:uc003vgk.3:exon1:c.8C>A:p.S3X,	uc003vgk.2	S	*	3	-30	rs114489785	NaN	NaN	NaN	3.513	0	None
chr11	68697795	68697797	A	SNP	G	nonsynonym	IGHMBP2	65	exonic	IGHMBP2:uc001ool.1:exon1:c.1A>G:p.M1V,	uc001ool.1	M	V	1	1	-	NaN	NaN	NaN	1.17	1.00E-04	None
chr14	105156075	105156077	G	SNP	A	splicing	INF2	231	splicing	-	-	-	-	-	-30	rs115602636	NaN	NaN	NaN	13.59	0	None
chr14	105173862	105173890	CCCCACCCCC	INDEL	CCCCACCCCC	nonframeshi	INF2	220	exonic	INF2:uc001ypb.2:exon8:c.1259_1285CCCCACCCCCACCCC,	-	-	-	-	301	-	NaN	NaN	NaN	NA	0	None
chr14	105174284	105174286	A	SNP	C	nonsynonym	INF2	26	exonic	INF2:uc001ypb.2:exon8:c.1681A>C:p.T561P,INF2:uc001y	uc001ypc.2	T	P	561	1	-	uc001ypb.2	T 561 P	possibly dam	12.58	0	0.90142298
chr14	105181652	105181654	A	SNP	G	nonsynonym	INF2	99	exonic	INF2:uc001ypb.2:exon22:c.3727A>G:p.K1243E,	uc001ypb.2	K	E	1243	1	-	uc001ypb.2	K 1243 E	benign	5.183	0	0.03153324
chr12	56089354	56089356	C	SNP	A	nonsynonym	ITGA7	140	exonic	ITGA7:uc009znx.3:exon12:c.1597G>T:p.A533S,ITGA7:uc	uc001shg.2	A	S	652	2	-	uc001shg.2	A 715 S	possibly dam	13.18	0	0.19797568
chr12	26596536	26596538	G	SNP	A	nonsynonym	ITPR2	154	exonic	ITPR2:uc009zjg.1:exon9:c.842C>T:p.S281L,ITPR2:uc001rl	uc009zjg.1	S	L	281	-2	-	uc001rhg.2	S 2130 L	benign	11.39	1.00E-04	0.04705433
chr12	26753040	26753042	T	SNP	C	nonsynonym	ITPR2	65	exonic;splicir	ITPR2:uc001rhg.3:exon29:c.3680A>G:p.N1227S,	uc001rhg.2	N	S	1227	-1	-	uc001rhg.2	N 1227 S	benign	2.582	0	0.01121172
chr12	26834804	26834807	AC	INDEL	ACTCAC	splicing	ITPR2	421	splicing	-	-	-	-	-	-30	-	NaN	NaN	NaN	NA	0	None
chr1	10386241	10386243	G	SNP	A	nonsynonym	KIF1B	173	exonic	KIF1B:uc001arb.3:exon25:c.2707G>A:p.D903N,KIF1B:uc	uc001aqz.2	D	N	917	1	-	uc001aqx.3	D 917 N	probably dam	25.8	1.00E-04	0.37034601
chr17	4923885	4923887	C	SNP	T	nonsynonym	KIF1C	132	exonic	KIF1C:uc002gan.2:exon20:c.1850C>T:p.P617L,	uc002gan.1	P	L	617	-3	-	uc002gan.1	P 617 L	benign	18.81	0	0.44552934
chr12	39716494	39716496	C	SNP	A	nonsynonym	KIF21A	94	exonic	KIF21A:uc001rlw.3:exon13:c.1597G>T:p.G533C,KIF21A:u	uc001rlv.2	G	C	221	-3	-	uc010skl.1	G 1196 C	probably dam	18.45	0	0.93174136
chr12	39724565	39724567	G	SNP	T	nonsynonym	KIF21A	88	exonic	KIF21A:uc001rlw.3:exon9:c.1252C>A:p.L418I,KIF21A:uc0	uc001rlv.2	L	I	113	2	-	uc001rlz.2	L 1065 I	probably dam	12.87	1.00E-04	0.27533039
chr12	39726877	39726879	A	SNP	G	nonsynonym	KIF21A	201	exonic	KIF21A:uc001rlw.3:exon5:c.470T>C:p.M157T,KIF21A:uc0	uc001rly.2	M	T	840	-3	-	uc001rlx.2	M 827 T	benign	11.69	1.00E-04	0.10269153
chr12	57974933	57974935	C	SNP	T	nonsynonym	KIF5A	127	exonic	KIF5A:uc010srr.1:exon21:c.2467C>T:p.R823W,KIF5A:uc0	uc010srr.1	R	W	823	-3	-	uc010srr.1	R 823 W	probably dam	20.2	0	0.97465366
chr1	170010856	170010859	AA	INDEL	A	frameshift_s	KIFAP3	60	exonic	KIFAP3:uc010ply.2:exon4:c.45_46T,	-	-	-	-	-30	-	NaN	NaN	NaN	NA	0	None
chr3	42733380	42733390	GAGGAGGA	INDEL	GAGGAG	nonframeshi	KLHL40	127	exonic	KLHL40:uc003clv.1:exon6:c.1762_1770GAGGAG,	-	-	-	-	301	-	NaN	NaN	NaN	NA	0	None
chr6	129371233	129371235	G	SNP	A	splicing	LAMA2	222	splicing	uc003qbn.3:exon2:c.283+1G>A,uc003qbo.3:exon2:c.283-	-	-	-	-	-30	-	NaN	NaN	NaN	21.5	1.00E-04	None
chr6	129498907	129498909	G	SNP	A	nonsynonym	LAMA2	117	exonic	LAMA2:uc021zfb.1:exon10:c.1364G>A:p.R455Q,LAMA2:	uc003qbn.2	R	Q	455	1	-	uc003qbn.2	R 455 Q	probably dam	24.2	1.00E-04	0.74790865
chr6	129612839	129612841	A	SNP	G	nonsynonym	LAMA2	106	exonic	LAMA2:uc021zfb.1:exon20:c.2831A>G:p.Q944R,LAMA2:	uc003qbn.2	Q	R	944	-3	-	uc003qbo.2	Q 944 R	benign	12.1	1.00E-04	0.19153166
chr6	129636904	129636906	A	SNP	T	splicing	LAMA2	122	splicing	uc003qbn.3:exon26:c.3736-2A>T,uc003qbo.3:exon26:c.3	-	-	-	-	-30	-	NaN	NaN	NaN	22.3	0	None
chr6	129802438	129802440	G	SNP	A	nonsynonym	LAMA2	109	exonic	LAMA2:uc021zfb.1:exon55:c.7604G>A:p.G2535D,LAMA2	uc003qbn.2	G	D	2533	-3	-	uc003qbn.2	G 2535 D	probably dam	27	1.00E-04	0.86261159
chr3	49160986	49160988	T	SNP	C	nonsynonym	LAMB2	140	exonic	LAMB2:uc003cwe.3:exon25:c.3875A>G:p.N1292S,	uc003cwe.2	N	S	1292	1	-	uc003cwe.2	N 1292 S	benign	9.951	0	0.19659663
chr3	49161383	49161385	C	SNP	A	nonsynonym	LAMB2	193	exonic	LAMB2:uc003cwe.3:exon24:c.3574G>T:p.A1192S,	uc003cwe.2	A	S	1192	1	-	uc003cwe.2	A 1192 S	benign	10.45	0	0.04267314
chrX	64749509	64749511	G	SNP	A	nonsynonym	LAS1L	586	exonic	LAS1L:uc004dwd.2:exon4:c.637C>T:p.H213Y,LAS1L:uc00	uc004dwc.1	H	Y	255	2	-	uc004dwa.1	H 255 Y	benign	3.116	0	None
chr11	18418147	18418150	CC	INDEL	C	frameshift_s	LDHA	81	exonic	LDHA:uc010rdd.2:exon1:c.54_55C,	-	-	-	-	-30	-	NaN	NaN	NaN	NA	0	None
chr22	30639773	30639775	A	SNP	G	nonsynonym	LIF	348	exonic	LIF:uc003agz.3:exon3:c.475T>C:p.Y159H,	uc003agz.2	Y	H	159	-6	-	uc003agz.2	Y 159 H	probably dam	21.8	0	None
chr1	156105033	156105035	C	SNP	T	nonsynonym	LMNA	140	exonic	LMNA:uc009wrp.3:exon1:c.50C>T:p.T17M,	uc009wrp.2	T	M	17	-1	-	NaN	NaN	NaN	9.888	1.00E-04	None
chr19	2431797	2431799	C	SNP	G	nonsynonym	LMNB2	112	exonic	LMNB2:uc002lvy.4:exon10:c.1693G>C:p.V565L,	uc002lwa.1	V	L	565	0	-	uc002lvy.2	V 545 L	benign	7.416	1.00E-04	0.58186096
chr3	69171496	69171502	AGAAG	INDEL	AG	nonframeshi	LMOD3	126	exonic	LMOD3:uc003dnt.2:exon2:c.37_41CT,LMOD3:uc003dns.2	-	-	-	-	301	-	NaN	NaN	NaN	NA	0	None
chr11	46896614	46896616	T	SNP	G	nonsynonym	LRP4	82	exonic	LRP4:uc001ndn.4:exon28:c.3965A>C:p.H1322P,	uc001ndn.3	H	P	1322	-2	-	uc001ndn.3	H 1322 P	probably dam	23	1.00E-04	0.90312916
chr1	235918940	235918942	C	SNP	G	nonsynonym	LYST	58	exonic	LYST:uc001hxj.3:exon25:c.7066G>C:p.D2356H,	uc001hxj.2	D	H	2356	-1	-	uc001hxj.2	D 2356 H	benign	11.39	0	0.26023567
chr1	235922494	235922496	C	SNP	T	nonsynonym	LYST	140	exonic	LYST:uc001hxj.3:exon23:c.6658G>A:p.D2220N,	uc001hxj.2	D	N	2220	1	-	uc001hxj.2	D 2220 N	benign	16.6	0	0.86046976
chr1	235972993	235972995	G	SNP	A	nonsynonym	LYST	180	exonic	LYST:uc001hxl.2:exon5:c.1124C>T:p.A375V,LYST:uc001h	uc001hxl.1	A	V	375	0	-	uc001hxl.1	A 375 V	benign	12.04	1.00E-04	0.13634152
chr11	47308015	47308017	A	SNP	G	nonsynonym	MADD	272	exonic	MADD:uc001neq.2:exon15:c.2584A>G:p.S862G,MADD:u	uc001ner.1	S	G	862	0	-	uc009yln.1	S 819 G	probably dam	28.6	0	0.56126522
chr17	44039818	44039820	C	SNP	T	nonsynonym	MAPT	195	exonic	MAPT:uc021tyw.1:exon2:c.116C>T:p.T39M,MAPT:uc002	uc002ijs.3	T	M	39	-1	-	uc002ijx.3	T 39 M	benign	4.542	1.00E-04	0.01297364
chr17	44061122	44061124	C	SNP	T	nonsynonym	MAPT	276	exonic	MAPT:uc021tyx.1:exon1:c.497C>T:p.S166L,MAPT:uc010c	uc010dau.2	S	L	318	-2	rs73314997	uc010dau.2	S 318 L	benign	14.68	0	0.01975875
chr17	44073865	44073867	A	SNP	C	nonsynonym	MAPT	66	exonic	MAPT:uc021tyw.1:exon7:c.571A>C:p.T191P,MAPT:uc002	uc002ijr.3	T	P	537	-1	-	uc002ijx.3	T 191 P	possibly dam	8.256	0	0.58914542
chr5	138643509	138643511	G	SNP	A	nonsynonym	MATR3	203	exonic	MATR3:uc010jfb.3:exon4:c.406G>A:p.E136K,MATR3:uc0	uc003ldx.2	E	K	136	1	-	uc003ldu.2	E 136 K	probably dam	20.4	0	0.90659624
chr1	29522472	29522474	C	SNP	T	stopgain_SN	MECR	181	exonic	MECR:uc001brp.1:exon9:c.716G>A:p.W239X,MECR:uc00	uc001brr.1	W	*	239	-30	-	NaN	NaN	NaN	37	0	None
chr1	29557360	29557362	G	SNP	C	nonsynonym	MECR	19	exonic	MECR:uc010ofz.1:exon1:c.58C>G:p.L20V,MECR:uc001br	uc010ofz.1	L	V	20	1	-	uc001brq.1	L 20 V	benign	15.89	0	0.0942933
chr1	12052716	12052718	G	SNP	T	nonsynonym	MFN2	122	exonic	MFN2:uc009vni.3:exon3:c.281G>T:p.R94L,MFN2:uc001a	uc009vni.2	R	L	94	-2	-	uc001atn.3	R 94 L	probably dam	31	0	0.9891708
chr1	12062084	12062086	C	SNP	G	nonsynonym	MFN2	277	exonic	MFN2:uc009vni.3:exon10:c.1085C>G:p.T362R,MFN2:uc0	uc001atn.3	T	R	362	-1	-	uc001atn.3	T 362 R	probably dam	20.7	0	0.99037028
chr2	190927060	190927062	G	SNP	A	nonsynonym	MSTN	308	exonic	MSTN:uc002urp.3:exon1:c.262C>T:p.R88W,	uc002urp.2	R	W	88	-7	-	uc002urp.2	R 88 W	possibly dam	18.58	0	0.93948436
chr1	155583295	155583297	T	SNP	C	nonsynonym	MSTO1	140	exonic	MSTO1:uc031ppy.1:exon11:c.640T>C:p.C214R,MSTO1:u	uc001fkw.2	C	R	443	-3	rs112258960	uc001fkw.2	C 443 R	probably dam	10.43	0	0.12132796
chr9	21818131	21818133	C	SNP	T	nonsynonym	MTAP	123	exonic	MTAP:uc031tcz.1:exon4:c.278C>T:p.T93I,MTAP:uc011lnl	uc003zpi.1	T	I	93	-6	-	uc011lnk.1	T 110 I	probably dam	25.9	0	0.96039128
chr4	100527931	100527933	C	SNP	A	nonsynonym	MTTP	124	exonic	MTTP:uc003hvc.4:exon12:c.1372C>A:p.L458M,MTTP:uc0	uc003hvc.3	L	M	458	-3	-	uc003hvc.3	L 458 M	probably dam	16.38	0	0.95097309
chr9	113457804	113457806	C	SNP	T	nonsynonym	MUSK	64	exonic	MUSK:uc022blu.1:exon4:c.481C>T:p.L161F,MUSK:uc022l	uc004bex.2	L	F	161	-4	-	uc004bey.2	L 161 F	possibly dam	17.32	0	0.23372352
chr19	50785051	50785053	C	SNP	T	nonsynonym	MYH14	53	exonic	MYH14:uc002prr.1:exon31:c.4369C>T:p.R1457W,MYH14	uc002prq.1	R	W	1465	-3	-	uc002prr.1	R 1457 W	probably dam	17.58	1.00E-04	0.82790779
chr17	10426936	10426938	G	SNP	A	nonsynonym	MYH2	253	exonic	MYH2:uc010coi.3:exon37:c.5348C>T:p.A1783V,MYH2:uc	uc002gmp.3	A	V	1783	-5	-	uc002gmp.3	A 1783 V	possibly dam	28.7	1.00E-04	0.97586399
chr14	23886122	23886124	A	SNP	G	nonsynonym	MYH7	272	exonic	MYH7:uc001wjx.3:exon33:c.4598T>C:p.L1533P,	uc001wjx.2	L	P	1533	-9	-	uc001wjx.2	L 1533 P	probably dam	23.7	0	0.9942199
chr14	23891397	23891399	C	SNP	T	nonsynonym	MYH7	114	exonic	MYH7:uc001wjx.3:exon25:c.3236G>A:p.R1079Q,	uc001wjx.2	R	Q	1079	5	-	uc001wjx.2	R 1079 Q	benign	16.44	0	0.69724393
chr14	23900798	23900800	G	SNP	A	nonsynonym	MYH7	140	exonic	MYH7:uc001wjx.3:exon8:c.727C>T:p.R243C,	uc001wjx.2	R	C	243	-14	-	uc001wjx.2	R 243 C	probably dam	16.5	0	0.99335629
chr22	26164334	26164336	G	SNP	A	nonsynonym	MYO18B	127	exonic	MYO18B:uc003aca.1:exon2:c.95G>A:p.G32D,MYO18B:u	uc010guz.1	G	D	32	-1	-	uc010guy.1	G 151 D	possibly dam	16.28	0	0.9575752
chr22	26165099	26165101	G	SNP	A	nonsynonym	MYO18B	232	exonic	MYO18B:uc003aca.1:exon2:c.860G>A:p.G287D,MYO18B	uc010guz.1	G	D	287	-1	-	uc003abz.1	G 406 D	benign	11.87	0	0.05868305
chr22	26422531	26422533	C	SNP	T	nonsynonym	MYO18B	140	exonic	MYO18B:uc003aca.1:exon41:c.6235C>T:p.R2079C,MYO1	uc010guz.1	R	C	2078	-3	-	uc010guz.1	R 2198 C	benign	10.47	1.00E-04	0.05199946
chr10	69895269	69895271	G	SNP	A	splicing	MYPN	40	splicing	-	-	-	-	-	-30	rs74878633	NaN	NaN	NaN	5.222	0	None
chr10	69918273	69918275	A	SNP	G	nonsynonym	MYPN	77	exonic	MYPN:uc009xpt.3:exon7:c.1349A>G:p.Q450R,MYPN:uc0	uc001jnl.1	Q	R	450	-5	-	uc010qit.1	Q 450 R	probably dam	25.7	0	0.96671283
chr5	69405798	69405801	TT	INDEL	T	frameshift_s	NAIP	293	exonic	NAIP:uc003jyj.1:exon1:c.445_446A,	-	-	-	-	-30	-	NaN	NaN	NaN	NA	0	None
chr5	70406475	70406477	G	SNP	C	nonsynonym	NAIP	33	exonic	NAIP:uc003kat.1:exon1:c.122C>G:p.A41G,	uc003kat.1	A	G	41	0	-	uc003kat.1	A 267 G	benign	0.036	0	None
chr5	70406595	70406597	A	SNP	G	nonsynonym	NAIP	51	exonic	NAIP:uc003kat.1:exon1:c.2T>C:p.M1T,	uc003kat.1	M	T	1	-1	-	uc003kat.1	M 227 T	benign	3.936	0	None
chr13	101712206	101712208	G	SNP	A	nonsynonym	NALCN	158	exonic	NALCN:uc001vox.1:exon42:c.4868C>T:p.T1623M,	uc001vox.1	T	M	1623	-1	-	uc001vox.1	T 1623 M	benign	3.958	1.00E-04	0.03022668
chr13	102030955	102030957	T	INDEL	TT	frameshift_s	NALCN	58	exonic	NALCN:uc001vox.1:exon4:c.340_340delinsAA,NALCN:uc	-	-	-	-	-30	-	NaN	NaN	NaN	NA	0	None
chr8	134251180	134251182	G	SNP	A	nonsynonym	NDRG1	140	exonic	NDRG1:uc003yue.2:exon3:c.113C>T:p.P38L,	uc003yug.2	T	T	375	0	-	NaN	NaN	NaN	10.49	0	None
chr2	152347010	152347012	C	SNP	T	nonsynonym	NEB	101	exonic	NEB:uc002txr.3:exon72:c.8723G>A:p.R2908Q,NEB:uc01	uc002txr.2	R	Q	2865	1	-	uc010zcb.1	R 304 Q	probably dam	20.3	1.00E-04	0.21655652
chr2	152348610	152348612	C	SNP	T	splicing	NEB	141	splicing	uc010zbz.2:exon9:c.796+1G>A,uc002txq.3:exon12:c.112	-	-	-	-	-30	-	NaN	NaN	NaN	17.62	0	None
chr2	152424651	152424653	C	SNP	A	nonsynonym	NEB	140	exonic	NEB:uc002txr.3:exon13:c.2072G>T:p.G691V,NEB:uc002t	uc002txr.2	G	V	648	-3	-	uc010fnx.2	G 4225 V	probably dam	25.6	0	0.99079508
chr2	152477475	152477477	A	SNP	T	nonsynonym	NEB	87	exonic	NEB:uc002txu.3:exon72:c.10517T>A:p.I3506N,NEB:uc02	uc010fnx.2	I	N	3251	-3	-	uc010fnx.2	I 3263 N	probably dam	28.4	0	0.96979576
chr2	152521939	152521941	C	SNP	A	nonsynonym	NEB	167	exonic	NEB:uc002txu.3:exon42:c.5145G>T:p.K1715N,NEB:uc02	uc010fnx.2	K	N	1715	0	-	uc010fnx.2	K 1715 N	benign	17.32	0	0.65901142
chr2	152522687	152522693	GTAGT	INDEL	GT	nonframeshi	NEB	107	exonic	NEB:uc002txu.3:exon41:c.4943_4947AC,NEB:uc021vrb.1	-	-	-	-	301	-	NaN	NaN	NaN	NA	0	None
chr2	152566277	152566279	C	SNP	T	splicing	NEB	98	splicing	uc021vrb.1:exon11:c.928-1G>A,uc031rpp.1:exon13:c.92	-	-	-	-	-30	-	NaN	NaN	NaN	23.6	0	None
chr2	152584315	152584346	TGCTGGCTG	INDEL	TGCTGGCTG	nonframeshi	NEB	167	exonic	NEB:uc002txu.3:exon5:c.154_183CTGGCACAGCCAGCA,N	-	-	-	-	301	-	NaN	NaN	NaN	NA	0	None
chr22	29884974	29884976	A	SNP	G	nonsynonym	NEFH	140	exonic	NEFH:uc003afo.3:exon4:c.1346A>G:p.K449R,	uc003afo.2	K	R	449	2	-	uc003afo.2	K 449 R	possibly dam	16.34	1.00E-04	None
chr22	29885127	29885135	AAACAAA	INDEL	AAACAAAAA	nonframeshi	NEFH	129	exonic	NEFH:uc003afo.3:exon4:c.1499_1505AAACAAAAACAAA,	-	-	-	-	301	-	NaN	NaN	NaN	NA	0	None
chr22	29885567	29885594	AAGTCCCCT	INDEL	AAGTCCCCT	nonframeshi	NEFH	553	exonic	NEFH:uc003afo.3:exon4:c.1939_1964AAGTCCCCTGAGA	-	-	-	-	301	-	NaN	NaN	NaN	NA	0	None
chr22	29885598	29885608	AGGAAGAG	INDEL	AGG	nonframeshi	NEFH	42	exonic	NEFH:uc003afo.3:exon4:c.1970_1978AGG,	-	-	-	-	301	-	NaN	NaN	NaN	NA	0	None
chr22	29885996	29886000	AAG	INDEL	''	nonframeshi	NEFH	0	exonic	NEFH:uc003afo.3:exon4:c.2368_2370del:p.790_790del,	-	-	-	-	301	-	NaN	NaN	NaN	NA	0	None
chr8	24810373	24810383	CTCCTCCTC	INDEL	CTCCTC	nonframeshi	NEFL	237	exonic	NEFL:uc003xee.4:exon5:c.1573_1581GAGGAG,	-	-	-	-	301	-	NaN	NaN	NaN	NA	0	None
chr8	24811096	24811098	G	SNP	A	nonsynonym	NEFL	119	exonic	NEFL:uc003xee.4:exon3:c.1382C>T:p.A461V,	uc003xee.2	A	V	461	0	-	uc003xee.2	A 461 V	benign	7.246	0	None
chr8	24811105	24811107	A	SNP	G	nonsynonym	NEFL	134	exonic	NEFL:uc003xee.4:exon3:c.1373T>C:p.I458T,	uc003xee.2	I	T	458	-1	-	uc003xee.2	I 458 T	benign	14.45	1.00E-04	None
chr15	23086364	23086391	GCCGCCGCC	INDEL	GCCGCCGCC	nonframeshi	NIPA1	201	exonic	NIPA1:uc001yvc.3:exon1:c.22_47GCGGCGGCGGCGGC	-	-	-	-	301	-	NaN	NaN	NaN	NA	0	None
chr3	9792057	9792059	C	SNP	T	nonsynonym	OGG1	146	exonic	OGG1:uc003bso.3:exon1:c.88C>T:p.R30C,OGG1:uc003bs	uc003bsn.2	R	C	30	-5	-	uc003bsj.2	R 30 C	probably dam	16.66	0	0.98134226
chr3	9807702	9807704	C	SNP	T	nonsynonym	OGG1	229	exonic	OGG1:uc003bsm.3:exon7:c.1159C>T:p.H387Y,OGG1:uc0	uc003bsn.2	H	Y	320	2	-	uc003bsm.2	H 387 Y	benign	9.331	0	0.35968819
chr3	193361162	193361164	C	SNP	A	nonsynonym	OPA1	100	exonic;splicir	OPA1:uc003ftj.3:exon13:c.1196C>A:p.T399N,OPA1:uc00	uc003ftm.2	T	N	381	-2	-	uc003ftm.2	T 381 N	probably dam	27.8	0	0.98729903
chr7	92123893	92123895	T	SNP	C	nonsynonym	PEX1	86	exonic	PEX1:uc003uly.3:exon18:c.2833A>G:p.I945V,PEX1:uc010	uc010ley.2	I	V	888	-4	-	uc011khs.1	I 623 V	possibly dam	17.55	0	0.47817916
chr12	48528572	48528574	T	SNP	A	nonsynonym	PFKM	98	exonic	PFKM:uc001rrc.3:exon8:c.708T>A:p.D236E,PFKM:uc001r	uc001rrg.1	D	E	236	-5	-	uc001rrc.2	D 236 E	benign	5.793	0	0.0875216
chrX	71813191	71813205	TTTTTTTTTT	INDEL	TTTTTTTTTT	frameshift_s	PHKA1	231	exonic	PHKA1:uc010nll.3:exon1:c.89_101AAAAAAAAAAAAAA,	-	-	-	-	-30	-	NaN	NaN	NaN	NA	0	None
chr16	47533725	47533727	T	SNP	C	nonsynonym	PHKB	140	exonic	PHKB:uc010vgi.1:exon4:c.205T>C:p.C69R,PHKB:uc002ee	uc002eeu.3	C	R	69	2	-	uc010vgi.1	C 69 R	benign	7.437	1.00E-04	0.17496068
chr16	47703149	47703160	GAAGAAGA	INDEL	GAAGAAG	nonframeshi	PHKB	136	exonic	PHKB:uc002eev.4:exon26:c.2452_2461GAAGAAG,PHKB:	-	-	-	-	301	-	NaN	NaN	NaN	NA	0	None
chr10	13330493	13330495	G	SNP	C	nonsynonym	PHYH	138	exonic	PHYH:uc001ime.3:exon5:c.244C>G:p.P82A,PHYH:uc001i	uc001imf.2	P	A	182	-6	-	uc001imf.2	P 182 A	probably dam	23.7	0	0.99274409
chr8	144991771	144991773	C	SNP	T	nonsynonym	PLEC	178	exonic	PLEC:uc003zab.1:exon32:c.12217G>A:p.D4073N,PLEC:uc	uc003zah.2	D	N	4059	-3	-	uc003zad.2	D 4073 N	probably dam	13.58	0	0.4759912
chr8	144995538	144995540	T	SNP	C	nonsynonym	PLEC	318	exonic	PLEC:uc003zab.1:exon32:c.8450A>G:p.D2817G,PLEC:uc0	uc003zaf.1	D	G	2954	-10	-	uc003zah.2	D 2803 G	probably dam	11.71	0	0.99431026
chr8	144996246	144996248	C	SNP	T	nonsynonym	PLEC	95	exonic	PLEC:uc003zab.1:exon32:c.7742G>A:p.R2581Q,PLEC:uc0	uc003zag.1	R	Q	2559	1	-	uc003zag.1	R 2559 Q	benign	11.03	1.00E-04	0.0702637
chr8	144997438	144997440	C	SNP	T	nonsynonym	PLEC	331	exonic	PLEC:uc003zab.1:exon31:c.6658G>A:p.A2220T,PLEC:uc0	uc003zae.1	A	T	2188	0	-	uc003zac.1	A 2224 T	benign	1.79	0	0.03732491
chr8	144997566	144997568	G	SNP	A	nonsynonym	PLEC	140	exonic	PLEC:uc003zab.1:exon31:c.6530C>T:p.T2177M,PLEC:uc0	uc003zad.2	T	M	2177	-1	-	uc003zac.1	T 2181 M	possibly dam	7.676	0	0.53380064
chr8	145009084	145009086	G	SNP	A	nonsynonym	PLEC	76	exonic	PLEC:uc003zab.1:exon9:c.838C>T:p.R280C,PLEC:uc003za	uc003zae.1	R	C	248	-3	-	uc003zab.1	R 280 C	probably dam	15.43	1.00E-04	0.95389068
chr8	145024632	145024634	T	SNP	G	nonsynonym	PLEC	36	exonic	PLEC:uc003zaf.1:exon1:c.242A>C:p.H81P,	uc003zaf.1	H	P	81	-8	-	uc003zaf.1	H 81 P	possibly dam	15.74	0	0.5586796
chr1	6529182	6529209	TCCTCCTCCT	INDEL	TCCTCTTCCT	nonframeshi	PLEKHG5	241	exonic	PLEKHG5:uc001ann.1:exon19:c.2254_2279GAGGAGGAG	-	-	-	-	301	-	NaN	NaN	NaN	NA	0	None
chr1	6550504	6550506	C	SNP	T	splicing	PLEKHG5	441	splicing	-	-	-	-	-	-30	rs11806429	NaN	NaN	NaN	12.84	0	None
chr1	12023643	12023645	G	SNP	A	nonsynonym	PLOD1	140	exonic	PLOD1:uc010obb.2:exon12:c.1294G>A:p.V432M,PLOD1:u	uc001atm.2	V	M	385	1	-	uc010obb.1	V 432 M	probably dam	28.8	1.00E-04	0.7838593
chr8	82357148	82357150	A	SNP	G	nonsynonym	PMP2	130	exonic	PMP2:uc003ycb.1:exon2:c.149T>C:p.I50T,	uc003ycb.1	I	T	50	-4	-	uc003ycb.1	I 50 T	possibly dam	26.4	0	0.04006172
chr19	50364572	50364574	C	SNP	T	nonsynonym	PNKP	133	exonic	PNKP:uc002pqi.3:exon16:c.1381G>A:p.E461K,PNKP:uc00	uc002pqj.2	E	K	500	1	-	uc002pqi.2	E 461 K	benign	5.731	0	0.19073734
chr11	824987	824996	TGCATGCA	INDEL	TGCA	frameshift_s	PNPLA2	985	exonic	PNPLA2:uc009ycl.3:exon4:c.1075_1082TGCA,	-	-	-	-	-30	-	NaN	NaN	NaN	NA	0	None
chr19	7615891	7615893	A	SNP	C	nonsynonym	PNPLA6	38	exonic	PNPLA6:uc010xjq.2:exon19:c.2110A>C:p.T704P,PNPLA6:	uc010xjp.1	T	P	630	-6	-	uc002mgq.1	T 656 P	benign	20.5	0	0.99567956
chr19	7615956	7615958	G	SNP	T	nonsynonym	PNPLA6	75	exonic	PNPLA6:uc010xjq.2:exon19:c.2175G>T:p.E725D,PNPLA6:	uc002mgs.2	E	D	716	-3	-	uc010xjq.1	E 716 D	benign	16.47	0	0.18054384
chr19	7619462	7619464	G	SNP	C	nonsynonym	PNPLA6	90	exonic	PNPLA6:uc010xjq.2:exon23:c.2518G>C:p.G840R,PNPLA6	uc010xjp.1	G	R	765	-2	-	uc002mgs.2	G 830 R	possibly dam	11.89	0	0.92001635
chr3	119187883	119187885	A	SNP	T	nonsynonym	POGLUT1	104	exonic	POGLUT1:uc003ecm.3:exon1:c.16A>T:p.S6C,	uc003ecm.2	S	C	6	-1	-	uc003ecm.2	S 6 C	benign	10.54	0	0.08177597
chr19	50887648	50887650	T	SNP	G	splicing	POLD1	21	splicing	-	-	-	-	-	-30	-	NaN	NaN	NaN	14.85	0	None
chr19	50919937	50919939	C	SNP	T	nonsynonym	POLD1	346	exonic	POLD1:uc010eny.4:exon23:c.3103C>T:p.R1035C,POLD1:u	uc002psb.3	R	C	1009	-3	-	uc010eny.2	R 1035 C	possibly dam	20.1	0	0.97151595
chr15	89876827	89876861	TGCTGCTGC	INDEL	TGCTGCTGC	nonframeshi	POLG	186	exonic	POLG:uc002bns.4:exon2:c.126_158GCAGCAGCAGCAGCA	-	-	-	-	301	-	NaN	NaN	NaN	NA	0	None
chr17	62486946	62486951	AAAA	INDEL	AA	frameshift_s	POLG2	10	exonic	POLG2:uc010deg.2:exon4:c.932_935TT,POLG2:uc002jei.	-	-	-	-	-30	-	NaN	NaN	NaN	NA	0	None
chr1	46658068	46658070	G	SNP	A	nonsynonym	POMGNT1	140	exonic	POMGNT1:uc001cph.1:exon1:c.58C>T:p.R20C,POMGNT1	uc010olz.1	R	C	299	-11	-	uc001cpe.2	R 442 C	probably dam	23.1	0	0.9985795
chr9	134390889	134390891	C	SNP	T	nonsynonym	POMT1	230	exonic	POMT1:uc004cav.3:exon13:c.1319C>T:p.A440V,POMT1:u	uc011mcl.1	A	V	266	-3	-	uc011mcn.1	A 143 V	possibly dam	14.44	1.00E-04	0.3472046
chr7	94947634	94947636	C	SNP	T	nonsynonym	PON1	57	exonic;splicir	PON1:uc003uns.3:exon2:c.145G>A:p.E49K,	uc003uns.2	E	K	49	1	-	uc003uns.2	E 49 K	possibly dam	18.55	1.00E-04	0.88526201
chr5	146258290	146258323	GCTGCTGCT	INDEL	GCTGCTGCT	nonframeshi	PPP2R2B	106	exonic	PPP2R2B:uc003log.5:exon1:c.26_57GCAGCAGCAGCAG	-	-	-	-	301	-	NaN	NaN	NaN	NA	0	None
chr2	44587178	44587180	T	SNP	A	splicing	PREPL	116	splicing	-	-	-	-	-	-30	-	NaN	NaN	NaN	3.588	0	None
chr20	4680093	4680095	C	SNP	T	nonsynonym	PRNP	134	exonic	PRNP:uc002wkw.4:exon2:c.139C>T:p.P47S,	uc002wku.2	P	P	76	0	rs112637437	NaN	NaN	NaN	1.23	0	None
chr19	40900179	40900203	TCCTCCTCCT	INDEL	TCCTCCTCCT	nonframeshi	PRX	39	exonic	PRX:uc002onr.3:exon7:c.4057_4079GAGGAGGAGGAGG	-	-	-	-	301	-	NaN	NaN	NaN	NA	0	None
chr19	40900522	40900524	T	SNP	G	nonsynonym	PRX	140	exonic	PRX:uc002onr.3:exon7:c.3736A>C:p.K1246Q,PRX:uc002o	uc002onr.2	K	Q	1246	1	-	uc002onr.2	K 1246 Q	probably dam	16.32	0	0.75170445

Chr	LeftFlank	RightFlank	ref_allele	muttype	var_allele	type	Gene_name	Qual	loc_type	consequence	transcript	ref_aa	var_aa	aa_pos	CDPred_scor	dbID	Polyphen_ts_	Polyphen_aa	Polyphen_an	CADD_Score	EXAC_POPM	ClinPred
chr19	40900536	40900538	C	SNP	T	nonsynonym	PRX	140	exonic	PRX:uc002onr.3:exon7:c.3722G>A:p.G1241D,PRX:uc002c	uc002onq.2	G	D	1102	-1	-	uc002onr.2	G 1241 D	probably dam	13.96	1.00E-04	0.55556786
chr19	40901322	40901324	C	SNP	T	nonsynonym	PRX	118	exonic	PRX:uc002onr.3:exon7:c.2936G>A:p.G979E,PRX:uc002or	uc002onr.2	G	E	979	-2	-	uc002onr.2	G 979 E	possibly dam	8.682	0	0.91279781
chr19	40901679	40901681	T	SNP	G	nonsynonym	PRX	62	exonic	PRX:uc002onr.3:exon7:c.2579A>C:p.D860A,PRX:uc002on	uc002onq.2	D	A	721	-2	-	uc002onr.2	D 860 A	probably dam	9.075	0	0.48989537
chr6	32810784	32810786	C	SNP	T	nonsynonym	PSMB8	140	exonic	PSMB8:uc003oce.3:exon2:c.229G>A:p.A77T,PSMB8:uc0(	uc003ocf.2	A	T	73	-7	-	uc003oce.2	A 77 T	probably dam	36	0	0.97664273
chr11	64514803	64514805	C	SNP	T	nonsynonym	PYGM	71	exonic	PYGM:uc001oax.4:exon18:c.2204G>A:p.R735H,PYGM:uc	uc001oax.3	R	H	735	2	-	uc001oax.3	R 735 H	benign	15.42	1.00E-04	0.65858871
chr7	127976096	127976098	C	SNP	T	splicing	RBM28	193	splicing	uc003vmp.2:exon8:c.614-1G>A,uc011koj.1:exon4:c.191-	-	-	-	-	-30	-	NaN	NaN	NaN	12.77	0	None
chr2	161223876	161223878	A	SNP	T	nonsynonym	RBMS1	86	exonic	RBMS1:uc002ubm.3:exon3:c.2T>A:p.M1K,RBMS1:uc002	uc002ubl.2	M	K	32	-1	-	uc002ubn.2	M 34 K	benign	22.4	0	0.94663972
chr8	101271358	101271360	C	SNP	T	nonsynonym	RNF19A	112	exonic	RNF19A:uc003yjk.2:exon10:c.1942G>A:p.G648S,RNF19A	uc003yjk.1	G	S	648	0	-	uc003yjj.1	G 648 S	benign	15.05	1.00E-04	0.06997599
chr11	124750447	124750461	CGGAGTCGG	INDEL	CGGAGTC	nonframeshi	ROBO3	915	exonic	ROBO3:uc001qbd.2:exon11:c.868_880CGGAGTC,ROBO3	-	-	-	-	301	-	NaN	NaN	NaN	NA	0	None
chr8	103250839	103250841	G	INDEL	GG	frameshift_s	RRM2B	117	exonic	RRM2B:uc022azl.1:exon1:c.211_211delinsCC,	-	-	-	-	-30	-	NaN	NaN	NaN	NA	0	None
chr19	38945967	38945969	G	SNP	A	nonsynonym	RYR1	257	exonic	RYR1:uc002oiu.3:exon14:c.1534G>A:p.E512K,RYR1:uc00	uc002oiu.2	E	K	512	5	-	uc002oiu.2	E 512 K	probably dam	15.95	0	0.96318388
chr19	38948770	38948772	A	SNP	G	nonsynonym	RYR1	140	exonic	RYR1:uc002oiu.3:exon18:c.2006A>G:p.E669G,RYR1:uc0(	uc002oit.2	E	G	669	1	-	uc002oit.2	E 669 G	possibly dam	14.07	0	0.88879639
chr19	38960073	38960075	T	SNP	C	nonsynonym	RYR1	223	exonic	RYR1:uc002oiu.3:exon27:c.3686T>C:p.M1229T,RYR1:uc(	uc002oit.2	M	T	1229	-1	-	uc002oiu.2	M 1229 T	possibly dam	10.31	0	0.88757968
chr19	38969090	38969092	T	SNP	C	nonsynonym	RYR1	109	exonic	RYR1:uc002oiu.3:exon31:c.4471T>C:p.C1491R,RYR1:uc0	uc002oiu.2	C	R	1491	-10	-	uc002oit.2	C 1491 R	probably dam	14.83	0	0.99895275
chr13	23911312	23911314	A	SNP	T	nonsynonym	SACS	103	exonic	SACS:uc001uon.3:exon10:c.6702T>A:p.F2234L,SACS:uc0	uc001uoo.2	F	L	2087	0	-	uc001uon.2	F 2234 L	benign	14.95	0	0.30140662
chr13	23928729	23928731	C	SNP	T	nonsynonym	SACS	107	exonic	SACS:uc001uop.1:exon1:c.1382G>A:p.G461D,SACS:uc00	uc001uoo.2	G	D	527	-1	-	uc001uop.1	G 461 D	probably dam	14.94	0	0.84409225
chr22	50900428	50900430	G	SNP	A	nonsynonym	SBF1	140	exonic	SBF1:uc003bli.2:exon20:c.2519C>T:p.T840M,SBF1:uc00	uc003blh.2	T	M	839	-1	-	uc003blh.2	T 839 M	probably dam	14.89	1.00E-04	None
chr22	50903538	50903540	C	SNP	T	nonsynonym	SBF1	140	exonic	SBF1:uc003bli.2:exon12:c.1226G>A:p.R409H,SBF1:uc00	uc003bli.2	R	H	409	-8	-	uc003blh.2	R 408 H	probably dam	27.8	0	None
chr22	50913214	50913216	C	SNP	G	nonsynonym	SBF1	72	exonic;splicir	SBF1:uc003bli.2:exon1:c.55G>C:p.G19R,SBF1:uc003blh.	uc003bli.2	G	R	19	0	-	uc003blh.2	G 19 R	possibly dam	12.68	0	None
chr11	9861054	9861056	G	SNP	C	nonsynonym	SBF2	86	exonic	SBF2:uc001mif.3:exon25:c.2713C>G:p.L905V,SBF2:uc00	uc001mib.2	L	V	1149	-1	-	uc001mib.2	L 1149 V	benign	18.04	0	0.29172274
chr2	167262288	167262290	G	SNP	T	nonsynonym	SCN7A	191	exonic	SCN7A:uc002udu.2:exon25:c.4850C>A:p.T1617K,	uc002udu.1	T	K	1617	-1	-	uc002udu.1	T 1617 K	probably dam	18.07	0	0.98859006
chr2	167266239	167266241	C	SNP	T	nonsynonym	SCN7A	105	exonic	SCN7A:uc002udu.2:exon24:c.3917G>A:p.R1306H,	uc002udu.1	R	H	1306	-3	-	uc002udu.1	R 1306 H	benign	10.68	0	0.09369857
chr17	10584501	10584503	G	SNP	C	nonsynonym	SCO1	114	exonic	SCO1:uc002gmr.4:exon6:c.840C>G:p.N280K,	uc002gms.3	N	K	249	-3	-	uc002gms.3	N 249 K	possibly dam	27.9	0	0.98114145
chr17	75484869	75484871	A	SNP	G	nonsynonym	SEPT9	83	exonic	SEPT9:uc002jty.4:exon5:c.433A>G:p.N145D,SEPT9:uc01(	uc002jtv.2	N	D	389	-5	-	uc010wtl.1	N 172 D	possibly dam	17.87	0	0.81069171
chr9	135203528	135203530	A	SNP	C	nonsynonym	SETX	230	exonic	SETX:uc010mzt.3:exon1:c.2313T>G:p.F771L,SETX:uc004	uc004cbk.2	F	L	1152	0	-	uc010mzt.2	F 1152 L	benign	9.514	0	0.07283013
chr9	135203910	135203929	TCATCATCAT	INDEL	TCATCATCAT	nonframeshi	SETX	114	exonic	SETX:uc010mzt.3:exon1:c.1914_1931TGATGATGATGAT	-	-	-	-	301	-	NaN	NaN	NaN	NA	0	None
chr9	135204769	135204771	C	SNP	T	nonsynonym	SETX	138	exonic	SETX:uc010mzt.3:exon1:c.1072G>A:p.G358R,SETX:uc004	uc004cbk.2	G	R	739	-2	-	uc010mzt.2	G 739 R	possibly dam	18.58	0	0.87898773
chr5	156184725	156184731	AGAAG	INDEL	AG	nonframeshi	SGCD	241	exonic	SGCD:uc003lwb.3:exon8:c.710_714AG,	-	-	-	-	301	-	NaN	NaN	NaN	NA	0	None
chr13	23771285	23771287	G	SNP	A	splicing	SGCG	91	splicing	-	-	-	-	-	-30	rs115711701	NaN	NaN	NaN	3.111	0	None
chr5	148392231	148392233	G	SNP	C	nonsynonym	SH3TC2	179	exonic	SH3TC2:uc010jgw.3:exon4:c.2051C>G:p.T684R,SH3TC2:u	uc010jgx.2	T	R	1033	-1	-	uc010jgx.2	T 1033 R	benign	2.364	1.00E-04	0.01975677
chr5	148406434	148406436	G	SNP	A	stopgain_SN	SH3TC2	140	exonic	SH3TC2:uc003lpv.1:exon12:c.1501C>T:p.R501X,SH3TC2:u	uc011dbz.1	R	*	839	-30	-	NaN	NaN	NaN	37	0	None
chr5	148407255	148407257	T	SNP	C	nonsynonym	SH3TC2	140	exonic	SH3TC2:uc003lpv.1:exon12:c.680A>G:p.Y227C,SH3TC2:u	uc010jgw.2	Y	C	324	-2	-	uc010jgw.2	Y 680 C	probably dam	17.3	1.00E-04	0.9963457
chr5	148418028	148418030	G	SNP	A	nonsynonym	SH3TC2	112	exonic	SH3TC2:uc003lpu.3:exon8:c.830C>T:p.T277M,SH3TC2:uc	uc011dbz.1	T	M	162	-1	-	uc011dbz.1	T 162 M	benign	12.01	1.00E-04	0.04260866
chr5	148422280	148422282	A	SNP	G	nonsynonym	SH3TC2	92	exonic	SH3TC2:uc003lpu.3:exon5:c.505T>C:p.Y169H,SH3TC2:uc(	uc003lpu.2	Y	H	169	2	-	uc003lpu.2	Y 169 H	possibly dam	13.76	0	0.04089982
chr17	73269538	73269540	T	SNP	C	nonsynonym	SLC25A19	140	exonic	SLC25A19:uc002jnv.4:exon8:c.956A>G:p.Q319R,SLC25A1	uc002jnt.3	Q	R	319	1	-	uc010dge.2	Q 262 R	benign	4.827	0	0.02624725
chr20	746113	746115	C	SNP	T	nonsynonym	SLC52A3	153	exonic	SLC52A3:uc002wed.4:exon2:c.305G>A:p.G102D,SLC52A:	uc002wee.2	G	D	102	-1	-	uc002wed.3	G 102 D	benign	0.088	0	0.10315216
chr2	108626991	108626993	A	SNP	G	nonsynonym	SLC5A7	240	exonic	SLC5A7:uc010fjj.3:exon9:c.1418A>G:p.Y473C,SLC5A7:uc(	uc010ywn.1	Y	C	360	-2	-	uc002tdv.2	Y 473 C	possibly dam	9.122	0	0.55472201
chr2	220347867	220347869	G	SNP	A	nonsynonym	SPEG	93	exonic	SPEG:uc010fwg.3:exon30:c.5683G>A:p.E1895K,	uc010fwg.2	E	K	1895	1	-	uc010fwg.2	E 1895 K	probably dam	16.09	0	0.9213565
chr15	44888343	44888345	C	SNP	G	nonsynonym	SPG11	231	exonic	SPG11:uc010ueh.2:exon25:c.4371G>C:p.W1457C,SPG11	uc001ztx.2	W	C	1457	-2	-	uc010uei.1	W 1457 C	possibly dam	16.31	0	0.67337423
chr15	44907653	44907655	G	SNP	A	nonsynonym	SPG11	141	exonic	SPG11:uc010ueh.2:exon16:c.2945C>T:p.T982I,SPG11:uc(	uc001ztx.2	T	I	982	-1	-	uc001ztx.2	T 982 I	benign	14.26	0	0.18115255
chr15	44912608	44912617	AAAAAAAA	INDEL	AAAAAAAAA	frameshift_s	SPG11	66	exonic	SPG11:uc001ztz.1:exon1:c.83_90TTTTTTTT,	-	-	-	-	-30	-	NaN	NaN	NaN	NA	0	None
chr13	36886362	36886364	G	SNP	A	nonsynonym	SPG20	97	exonic	SPG20:uc001uvn.3:exon9:c.1652C>T:p.T551I,SPG20:uc0(	uc001uvo.2	T	I	551	-2	-	uc010ten.1	T 551 I	probably dam	23.3	0	0.92013627
chr16	89576939	89576941	A	SNP	G	nonsynonym	SPG7	88	exonic	SPG7:uc002fni.3:exon2:c.226A>G:p.N76D,SPG7:uc002fn	uc002fni.2	N	D	76	1	-	uc002fnj.2	N 76 D	benign	5.754	0	0.02401603
chr16	89597198	89597200	T	SNP	A	nonsynonym	SPG7	112	exonic	SPG7:uc002fni.3:exon7:c.970T>A:p.F324I,SPG7:uc002fnj	uc002fni.2	F	I	324	5	-	uc002fnj.2	F 324 I	probably dam	24.4	0	0.97971218
chr16	89615453	89615455	G	SNP	A	nonsynonym	SPG7	80	exonic	SPG7:uc002fnk.2:exon7:c.154G>A:p.D52N,	uc002fnk.2	D	N	52	1	-	NaN	NaN	NaN	3.501	0	0.06612203
chr19	41062929	41062931	T	SNP	G	nonsynonym	SPTBN4	25	exonic;splicir	SPTBN4:uc002ony.3:exon26:c.5291T>G:p.V1764G,SPTBN	uc002ony.2	V	G	1764	-1	-	uc010egx.2	V 507 G	probably dam	19.34	0	0.99655801
chr19	41062973	41062975	G	SNP	A	nonsynonym	SPTBN4	175	exonic	SPTBN4:uc002ony.3:exon26:c.5335G>A:p.A1779T,SPTBN	uc002ony.2	A	T	1779	-4	-	uc002ony.2	A 1779 T	possibly dam	10.44	0	0.70023036
chr9	94809515	94809517	G	SNP	A	nonsynonym	SPTLC1	132	exonic	SPTLC1:uc004arl.1:exon11:c.1019C>T:p.S340L,SPTLC1:uc	uc011ltv.1	S	L	340	-8	-	uc004arl.1	S 340 L	probably dam	33	1.00E-04	0.99031758
chr5	179250914	179250916	A	SNP	T	nonsynonym	SQSTM1	238	exonic	SQSTM1:uc003mkx.3:exon2:c.107A>T:p.N36I,SQSTM1:u	uc003mkx.2	N	I	36	-3	-	uc003mkw.3	N 120 I	benign	16.25	0	0.69377649
chr9	136219586	136219588	T	SNP	C	nonsynonym	SURF1	273	exonic	SURF1:uc004cdh.1:exon6:c.550A>G:p.R184G,	uc004cdg.1	R	G	75	-8	-	uc004cdh.1	R 184 G	possibly dam	16.71	0	0.98911661
chr3	12228960	12228984	TCTTCCTCCT(	INDEL	TCTTCCTCCT(	nonframeshi	SYN2	410	exonic	SYN2:uc003bwn.3:exon9:c.484_506TCTTCCTCCTC,SYN2:	-	-	-	-	301	-	NaN	NaN	NaN	NA	0	None
chr6	152451861	152451863	G	SNP	A	nonsynonym	SYNE1	140	exonic	SYNE1:uc003qou.4:exon145:c.26146C>T:p.H8716Y,SYNE	uc003qos.3	H	Y	3240	2	-	uc010kiw.2	H 8716 Y	benign	16.4	0	0.9538601
chr6	152470754	152470756	T	SNP	C	nonsynonym	SYNE1	116	exonic	SYNE1:uc003qou.4:exon136:c.24499A>G:p.I8167V,SYNE	uc003qop.3	I	V	329	-2	-	uc003qoq.3	I 369 V	benign	15.12	1.00E-04	0.26476732
chr6	152644649	152644651	T	SNP	G	nonsynonym	SYNE1	40	exonic	SYNE1:uc003qou.4:exon82:c.15880A>C:p.M5294L,SYNE1	uc003qou.3	M	L	5294	2	-	uc003qot.3	M 5223 L	benign	5.211	1.00E-04	0.01382485
chr6	152644762	152644764	G	SNP	T	nonsynonym	SYNE1	60	exonic	SYNE1:uc003qou.4:exon82:c.15767C>A:p.T5256K,SYNE1	uc003qot.3	T	K	5185	-1	-	uc003qot.3	T 5185 K	benign	2.586	0	0.21383236
chr6	152646393	152646395	G	SNP	A	nonsynonym	SYNE1	90	exonic	SYNE1:uc003qou.4:exon81:c.15482C>T:p.A5161V,SYNE1	uc010kiw.2	A	V	5161	-4	-	uc010kiz.2	A 5161 V	benign	12.27	0	0.42708623
chr6	152647180	152647182	A	SNP	G	nonsynonym	SYNE1	90	exonic	SYNE1:uc003qou.4:exon80:c.15350T>C:p.M5117T,SYNE1	uc003qot.3	M	T	5046	-5	-	uc010kiz.2	M 5117 T	probably dam	16.61	0	0.46143356
chr6	152652720	152652722	T	SNP	C	nonsynonym	SYNE1	140	exonic	SYNE1:uc003qou.4:exon78:c.13099A>G:p.I4367V,SYNE1	uc010kiz.2	I	V	122	3	-	uc003qou.3	I 4367 V	benign	0.847	0	0.10961259
chr6	152722349	152722351	T	SNP	C	nonsynonym	SYNE1	114	exonic	SYNE1:uc003qou.4:exon47:c.6952A>G:p.T2318A,SYNE1:	uc003qot.3	T	A	2325	0	-	uc003qot.3	T 2325 A	benign	10.1	0	0.08855259
chr6	152823765	152823767	A	SNP	T	splicing	SYNE1	65	splicing	uc003qot.4:exon11:c.909+2T>A,uc003qou.4:exon11:c.88	-	-	-	-	-30	-	NaN	NaN	NaN	17.19	0	None
chr14	64453330	64453332	T	SNP	C	nonsynonym	SYNE2	115	exonic	SYNE2:uc001xgl.3:exon19:c.2309T>C:p.L770S,SYNE2:uc(	uc001xgl.2	L	S	770	-2	-	uc001xgl.2	L 770 S	probably dam	12.91	0	0.95218813
chr14	64457711	64457713	C	SNP	G	nonsynonym	SYNE2	83	exonic	SYNE2:uc001xgl.3:exon21:c.2525C>G:p.P842R,SYNE2:uc	uc001xgl.2	P	R	842	-2	-	uc001xgm.2	P 842 R	benign	10.86	0	0.18484244
chr14	64486749	64486751	C	SNP	T	nonsynonym	SYNE2	55	exonic	SYNE2:uc001xgl.3:exon35:c.5090C>T:p.S1697F,SYNE2:u	uc001xgm.2	S	F	1697	-2	-	uc001xgl.2	S 1697 F	benign	12.75	0	0.41384098
chr14	64514816	64514818	A	SNP	G	nonsynonym	SYNE2	79	exonic	SYNE2:uc001xgl.3:exon46:c.7321A>G:p.N2441D,SYNE2:u	uc001xgl.2	N	D	2441	1	-	uc001xgl.2	N 2441 D	benign	11.37	0	0.32703424
chr14	64608747	64608749	A	SNP	G	nonsynonym	SYNE2	140	exonic	SYNE2:uc001xgn.3:exon3:c.134A>G:p.D45G,SYNE2:uc00	uc001xgm.2	D	G	5083	-6	-	uc001xgm.2	D 5083 G	benign	6.095	0	0.02043282
chr11	7324356	7324358	G	SNP	T	nonsynonym	SYT9	105	exonic	SYT9:uc001mfe.3:exon2:c.233G>T:p.C78F,	uc001mfe.2	C	F	78	-2	-	uc001mfe.2	C 78 F	benign	16.19	0	0.39121538
chr17	34171806	34171848	GGAGGAGA	INDEL	GGAGGAGA	nonframeshi	TAF15	501	exonic	TAF15:uc002hkd.4:exon15:c.1504_1544GGAGGAGATCG	-	-	-	-	301	-	NaN	NaN	NaN	NA	0	None
chr17	34171894	34171896	A	SNP	G	nonsynonym	TAF15	343	exonic	TAF15:uc002hkd.4:exon15:c.1592A>G:p.Y531C,TAF15:uc	uc002hkc.2	Y	C	528	-2	-	uc002hkd.2	Y 531 C	benign	12.46	1.00E-04	0.06040257
chr22	20052090	20052092	C	SNP	T	nonsynonym	TANGO2	90	exonic	TANGO2:uc010grw.2:exon10:c.737C>T:p.A246V,TANGO	uc002zrf.2	A	V	184	-4	-	uc002zrc.1	A 246 V	benign	6.811	0	0.06433486
chr6	134210727	134210729	A	SNP	G	nonsynonym	TCF21	101	exonic	TCF21:uc003qei.4:exon1:c.193A>G:p.K65E,TCF21:uc003c	uc003qei.3	K	E	65	1	-	uc003qei.3	K 65 E	benign	16.29	0	0.06721938
chr14	90489419	90489424	TGGT	INDEL	T	frameshift_s	TDP1	110	exonic	TDP1:uc010atn.3:exon16:c.1689_1690T,	-	-	-	-	-30	-	NaN	NaN	NaN	NA	0	None
chr11	85342836	85342838	A	SNP	G	nonsynonym	TMEM126B	50	exonic	TMEM126B:uc001pao.4:exon3:c.98A>G:p.Y33C,TMEM1	uc001pap.2	Y	C	33	-7	-	uc001pan.1	Y 33 C	benign	2.395	0	0.0222801
chr12	64202605	64202607	G	SNP	A	nonsynonym	TMEM5	92	exonic	TMEM5:uc001srq.2:exon6:c.1066G>A:p.V356M,TMEM5:	uc001srq.1	V	M	356	1	-	uc001srq.1	V 356 M	possibly dam	15.83	1.00E-04	0.59951574
chr6	31979405	31979407	G	SNP	A	nonsynonym	TNXB	69	exonic	TNXB:uc021yvf.2:exon2:c.448C>T:p.R150W,	uc011dpc.1	R	W	150	-3	-	uc011dpc.1	R 3766 W	probably dam	4.061	0	None
chr6	31979461	31979463	C	SNP	T	nonsynonym	TNXB	36	exonic	TNXB:uc021yvf.2:exon2:c.392G>A:p.R131H,	uc011dpc.1	R	H	131	0	-	uc011dpc.1	R 3747 H	probably dam	1.873	0	None
chr6	32010501	32010503	G	SNP	A	nonsynonym	TNXB	69	exonic	TNXB:uc003nzl.2:exon39:c.12026C>T:p.P4009L,TNXB:uc(	uc003nzh.1	P	L	478	-3	-	uc003nzl.2	P 4009 L	probably dam	11.81	0	0.86017227
chr6	32029205	32029207	C	SNP	T	nonsynonym	TNXB	82	exonic	TNXB:uc003nzl.2:exon21:c.7460G>A:p.R2487H,	uc003nzl.2	R	H	2487	0	-	uc003nzl.2	R 2487 H	probably dam	14.07	0	0.97874177
chr6	32037476	32037478	C	SNP	T	nonsynonym	TNXB	97	exonic	TNXB:uc003nzl.2:exon15:c.5440G>A:p.D1814N,	uc003nzl.2	D	N	1814	1	-	uc003nzl.2	D 1814 N	possibly dam	17.32	0	0.73719317
chr6	32063680	32063682	C	SNP	T	nonsynonym	TNXB	393	exonic	TNXB:uc003nzl.2:exon3:c.1949G>A:p.R650H,	uc003nzl.2	R	H	650	0	rs17201602(	uc003nzl.2	R 650 H	probably dam	11	0	0.02223468
chr4	184606528	184606530	A	SNP	G	nonsynonym	TRAPPC11	125	exonic	TRAPPC11:uc003ivw.3:exon17:c.1735A>G:p.I579V,TRAPI	uc003ivy.2	I	V	185	3	-	uc003ivw.2	I 579 V	benign	13.78	0	0.44373915
chr6	123786032	123786043	AAAAAAAAA	INDEL	AAAAAAAAA	frameshift_s	TRDN	156	exonic	TRDN:uc010ken.3:exon9:c.880_889TTTTTTTTT,	-	-	-	-	-30	-	NaN	NaN	NaN	NA	0	None
chr6	123837298	123837312	TTTTTCTTTT	INDEL	TTTTTCT	nonframeshi	TRDN	48	exonic	TRDN:uc010ken.3:exon6:c.525_537AGAAAAA,TRDN:uc0	-	-	-	-	301	-	NaN	NaN	NaN	NA	0	None
chr4	154191576	154191578	C	SNP	T	nonsynonym	TRIM2	279	exonic	TRIM2:uc003ini.1:exon2:c.94C>T:p.R32C,TRIM2:uc003ini	uc003inh.2	R	C	41	-4	-	uc003ing.2	R 14 C	probably dam	31	1.00E-04	0.76335657
chr15	64701882	64701884	A	SNP	G	nonsynonym	TRIP4	138	exonic	TRIP4:uc002anm.3:exon7:c.899A>G:p.K300R,	uc002anm.2	K	R	300	2	-	uc002anm.2	K 300 R	benign	22.4	1.00E-04	0.152957
chr12	110224530	110224532	G	SNP	A	nonsynonym	TRPV4	141	exonic	TRPV4:uc001tpk.2:exon14:c.2320C>T:p.R774C,TRPV4:uc	uc001tpk.1	R	C	774	-3	-	uc001tpj.1	R 774 C	possibly dam	17.49	1.00E-04	0.78040302
chr12	110252564	110252566	C	SNP	T	nonsynonym	TRPV4	31	exonic	TRPV4:uc001tpk.2:exon2:c.37G>A:p.G13R,TRPV4:uc021	uc001tpk.1	G	R	13	-2	-	uc001tpg.1	G 13 R	probably dam	11.55	1.00E-04	0.091179
chr2	179404205	179404207	C	SNP	A	nonsynonym	TTN	194	exonic	TTN:uc021vta.2:exon181:c.71766G>T:p.E23922D,TTN:uc	uc010zfi.1	E	D	23918	-5	-	uc010zfh.1	E 23989 D	probably dam	13.07	0	0.3826479
chr2	179404267	179404269	C	SNP	A	nonsynonym	TTN	356	exonic	TTN:uc021vta.2:exon181:c.71704G>T:p.A23902S,TTN:uc	uc010zfj.1	A	S	23773	1	-	uc010zfi.1	A 23902 S	probably dam	22.6	0	0.41646316
chr2	179404836	179404838	G	SNP	T	nonsynonym	TTN	226	exonic	TTN:uc021vta.2:exon180:c.71236C>A:p.P23746T,TTN:uc(	uc010zfi.1	P	T	23742	-1	-	uc010zfg.1	P 31045 T	probably dam	15.5	0	0.45078269
chr2	179413173	179413175	C	SNP	T	nonsynonym	TTN	334	exonic	TTN:uc021vta.2:exon168:c.66359G>A:p.R22120H,TTN:u	uc010zfj.1	R	H	21991	3	-	uc010zfj.1	R 21995 H	probably dam	21	1.00E-04	0.61228377
chr2	179416433	179416435	C	SNP	T	nonsynonym	TTN	246	exonic	TTN:uc021vta.2:exon164:c.64373G>A:p.R21458H,TTN:u	uc010zfi.1	R	H	21454	-6	-	uc010zfh.1	R 21525 H	probably dam	17.61	1.00E-04	0.89690137
chr2	179425012	179425014	G	SNP	A	nonsynonym	TTN	245	exonic	TTN:uc021vta.2:exon155:c.59026C>T:p.R19676W,TTN:u	uc010zfg.1	R	W	26040	-7	-	uc010zfi.1	R 19676 W	probably dam	11.2	0	0.64891505
chr2	179426556	179426558	G	SNP	A	nonsynonym	TTN	184	exonic	TTN:uc021vta.2:exon155:c.57482C>T:p.T19161I,TTN:uc0	uc010zfi.1	T	I	19157	-4	-	uc010zfg.1	T 26460 I	benign	11.25	0	0.37542414
chr2	179429187	179429189	T	SNP	C	nonsynonym	TTN	201	exonic	TTN:uc021vta.2:exon155:c.54851A>G:p.N18284S,TTN:u	uc010zfi.1	N	S	18280	3	-	uc010zfj.1	N 18159 S	probably dam	13.38	0	0.12695584
chr2	179437756	179437758	C	SNP	A	nonsynonym	TTN	144	exonic	TTN:uc021vta.2:exon155:c.46282G>T:p.D15428Y,TTN:u	uc010zfj.1	D	Y	15299	-6	-	uc010zfi.1	D 15428 Y	probably dam	13.5	0	0.64706439
chr2	179437785	179437787	T	SNP	C	nonsynonym	TTN	140	exonic	TTN:uc021vta.2:exon155:c.46253A>G:p.Y15418C,TTN:uc	uc010zfi.1	Y	C	15414	-14	-	uc010zfh.1	Y 15485 C	probably dam	12.03	1.00E-04	0.39448243
chr2	179438142	179438144	A	SNP	T	nonsynonym	TTN	195	exonic	TTN:uc021vta.2:exon155:c.45896T>A:p.M15299K,TTN:uc	uc010zfi.1	M	K	15295	-9	-	uc010zfj.1	M 15174 K	benign	11.59	0	0.32839394
chr2	179439083	179439085	A	SNP	C	nonsynonym	TTN	348	exonic	TTN:uc021vta.2:exon155:c.44955T>G:p.I14985M,TTN:u	uc010zfh.1	I	M	15044	1	-	uc010zfi.1	I 14985 M	possibly dam	2.187	0	0.1282983
chr2	179446426	179446428	C	SNP	G	nonsynonym	TTN	330	exonic	TTN:uc021vta.2:exon145:c.39748G>C:p.G13250R,TTN:u	uc010zfi.1	G	R	13246	-4	-	uc010zfj.1	G 13125 R	probably dam	17.26	0	0.77339423
chr2	179447684	179447686	T	SNP	C	nonsynonym	TTN	129	exonic	TTN:uc021vta.2:exon142:c.39025A>G:p.I13009V,TTN:uc	uc010zfj.1	I	V	12880	1	-	uc010zfh.1	I 13076 V	benign	14.64	0	0.24567722
chr2	179455397	179455399	C	SNP	T	nonsynonym	TTN	144	exonic	TTN:uc021vta.2:exon133:c.34234G>A:p.G11412R,TTN:u	uc010zfi.1	G	R	11408	-7	-	uc010zfg.1	G 18711 R	probably dam	13.57	0	0.56014591
chr2	179460427	179460429	T	SNP	A	nonsynonym	TTN	161	exonic	TTN:uc021vta.2:exon124:c.30833A>T:p.N10278I,TTN:uc	uc010zfg.1	N	I	16642	-5	-	uc010zfj.1	N 10153 I	probably dam	17.33	0	0.89431769
chr2	179479224	179479226	C	SNP	T	nonsynonym	TTN	252	exonic	TTN:uc021vta.2:exon90:c.22196G>A:p.R7399Q,TTN:uc0	uc010zfi.1	R	Q	7399	4	-	uc010zfg.1	R 14698 Q	probably dam	17.39	0	0.51268456
chr2	179511767	179511790	TATATATATA	INDEL	TATATATATA	splicing	TTN	171	splicing	-	-	-	-	-	-30	-	NaN	NaN	NaN	NA	0	None
chr2	179514542	179514544	C	SNP	A	splicing	TTN	116	splicing	uc031rqc.1:exon212:c.39895+1G>T,uc031rqd.1:exon167	-	-	-	-	-30	-	NaN	NaN	NaN	19.24	0	None
chr2	179514941	179514985	TTTCCTCTTC	INDEL	TTTCCTCTTC	nonframeshi	TTN	93	exonic	TTN:uc031rqd.1:exon165:c.35203_35245GAGGAAATTG(	-	-	-	-	301	-	NaN	NaN	NaN	NA	0	None
chr2	179530115	179530117	G	SNP	A	nonsynonym	TTN	92	exonic	TTN:uc010zfk.2:exon10:c.469C>T:p.P157S,TTN:uc031rqc	uc010zfk.1	P	S	157	-1	-	uc010zfk.1	P 285 S	possibly dam	12.9	1.00E-04	0.08519718
chr2	179544685	179544701	TCTTCTTCTT(	INDEL	TCTTCTTCTT(	nonframeshi	TTN	109	exonic	TTN:uc031rqd.1:exon138:c.32550_32564AGAAGAAGAA	-	-	-	-	301	-	NaN	NaN	NaN	NA	0	None
chr2	179556742	179556744	C	SNP	T	nonsynonym	TTN	81	exonic;splicir	TTN:uc031rqd.1:exon119:c.30811G>A:p.V10271I,TTN:uc	uc010fre.1	V	I	455	3	-	uc010fre.1	V 10271 I	possibly dam	16.58	0	0.53833038
chr2	179577850	179577852	C	SNP	A	nonsynonym	TTN	159	exonic	TTN:uc031rqd.1:exon91:c.26059G>T:p.A8687S,TTN:uc0(	uc002umz.1	A	S	4421	-2	-	uc010zfg.1	A 8687 S	possibly dam	12.17	0	0.39625288
chr2	179582314	179582316	T	SNP	C	nonsynonym	TTN	134	exonic	TTN:uc031rqd.1:exon85:c.24335A>G:p.Q8112R,TTN:uc0	uc010zfg.1	Q	R	7185	-1	-	uc010zfg.1	Q 8112 R	benign	10.03	1.00E-04	0.11284524
chr2	179585856	179585858	C	SNP	G	nonsynonym	TTN	267	exonic	TTN:uc031rqd.1:exon77:c.21938G>C:p.C7313S,TTN:uc00	uc002umz.1	C	S	3047	-16	-	uc010zfg.1	C 7313 S	probably dam	14.44	1.00E-04	0.18639604
chr2	179595891	179595893	C	SNP	T	nonsynonym	TTN	140	exonic	TTN:uc031rqd.1:exon58:c.16549G>A:p.V5517I,TTN:uc00	uc010zfg.1	V	I	4590	-5	-	uc010zfg.1	V 5517 I	probably dam	14.04	1.00E-04	0.15379066
chr2	179611099	179611101	T	SNP	C	nonsynonym	TTN	271	exonic	TTN:uc002unb.3:exon46:c.16027A>G:p.M5343V,	uc002unb.2	M	V	5343	1	-	uc002unb.2	M 5343 V	probably dam	15.49	0	0.18353079
chr2	179612062	179612064	C	SNP	T	nonsynonym	TTN	287	exonic	TTN:uc002unb.3:exon46:c.15064G>A:p.G5022S,	uc002unb.2	G	S	5022	0	-	uc002unb.2	G 5022 S	benign	5.525	0	0.06182663
chr2	179613846	179613848	A	SNP	T	nonsynonym	TTN	111	exonic	TTN:uc002unb.3:exon46:c.13280T>A:p.I4427N,	uc002unb.2	I	N	4427	-3	-	uc002unb.2	I 4427 N	benign	13.93	0	0.04615452
chr2	179614282	179614284	C	SNP	T	nonsynonym	TTN	102	exonic	TTN:uc002unb.3:exon46:c.12844G>A:p.V4282I,	uc002unb.2	V	I	4282	3	-	uc002unb.2	V 4282 I	benign	11.59	0	0.08330393
chr2	179614677	179614679	A	SNP	C	nonsynonym	TTN	140	exonic	TTN:uc002unb.3:exon46:c.12449T>G:p.L4150W,	uc002unb.2	L	W	4150	-2	-	uc002unb.2	L 4150 W	possibly dam	17.7	0	0.32780614
chr2	179614760	179614762	T	SNP	G	nonsynonym	TTN	140	exonic	TTN:uc002unb.3:exon46:c.12366A>C:p.E4122D,	uc002unb.2	E	D	4122	2	-	uc002unb.2	E 4122 D	benign	3.505	0	0.06540411
chr2	179644847	179644849	C	SNP	G	nonsynonym	TTN	89	exonic	TTN:uc021vta.2:exon21:c.3470G>C:p.G1157A,TTN:uc031	uc010zfh.1	G	A	1157	0	-	uc002unb.2	G 1203 A	benign	12.43	1.00E-04	0.05503256
chr2	179664399	179664401	C	SNP	G	nonsynonym	TTN	140	exonic	TTN:uc021vta.2:exon6:c.728G>C:p.G243A,TTN:uc031rqd	uc010zfg.1	G	A	243	0	-	uc002unb.2	G 243 A	possibly dam	18.31	0	0.41243887
chr2	220115570	220115572	C	SNP	T	nonsynonym	TUBA4A	270	exonic	TUBA4A:uc002vkt.1:exon4:c.850G>A:p.E284K,TUBA4A:u	uc010zkz.1	E	K	269	-5	-	uc002vkt.1	E 284 K	probably dam	19.53	0	0.99838519
chr19	17717158	17717160	G	SNP	C	nonsynonym	UNC13A	282	exonic	UNC13A:uc031rjv.1:exon42:c.4804C>G:p.L1602V,UNC13	uc002nhd.2	L	V	1693	2	-	uc002nhd.2	L 1606 V	benign	15.04	0	0.30324882
chr19	17769063	17769065	T	SNP	C	nonsynonym	UNC13A	140	exonic	UNC13A:uc031rjv.1:exon9:c.574A>G:p.S192G,UNC13A:u	uc002nhd.2	S	G	280	0	-	uc002nhd.2	S 192 G	probably dam	22.1	1.00E-04	0.81077522
chr20	56993373	56993375	C	SNP	T	nonsynonym	VAPB	150	exonic	VAPB:uc002xzd.2:exon2:c.166C>T:p.P56S,VAPB:uc002x:	uc002xza.2	P	S	56	-10	-	uc002xzc.2	P 56 S	probably dam	35	0	0.99954116
chr20	57016039	57016048	TTCTTCTT	INDEL	TTCTT	nonframeshi	VAPB	121	exonic	VAPB:uc010zzo.2:exon3:c.105_112TTCTT,VAPB:uc002x:	-	-	-	-	301	-	NaN	NaN	NaN	NA	0	None
chr9	35057162	35057164	G	SNP	A	nonsynonym	VCP	140	exonic	VCP:uc003zvy.2:exon17:c.2372C>T:p.T791I,VCP:uc010mk	uc003zvy.2	T	I	791	-1	-	uc003zvy.2	T 791 I	benign	13.53	0	0.64923465
chr6	43738779	43738781	G	SNP	C	nonsynonym	VEGFA	56	exonic	VEGFA:uc003owi.3:exon1:c.337G>C:p.A113P,VEGFA:uc(	uc003owg.2	A	P	113	-1	-	uc003owe.2	A 113 P	possibly dam	13.78	0	0.62689674
chr6	43748488	43748490	G	SNP	A	nonsynonym	VEGFA	94	exonic	VEGFA:uc021yzu.1:exon6:c.980G>A:p.G327E,VEGFA:uc(	uc003owf.2	G	E	328	-2	-	uc003owg.2	G 148 E	probably dam	25	0	0.92908055
chr14	97313608	97313610	G	SNP	A	nonsynonym	VRK1	68	exonic	VRK1:uc001yft.3:exon5:c.302G>A:p.R101H,	uc001yft.2	R	H	101	-4	-	uc001yft.2	R 101 H	benign	11.95	0	0.44332302
chr14	105268242	105268244	A	SNP	G	nonsynonym	ZBTB42	118	exonic	ZBTB42:uc001ypp.3:exon2:c.709A>G:p.S237G,ZBTB42:u	uc001ypp.2	S	G	237	0	-	uc001ypp.2	S 237 G	benign	1.066	0	0.05083537
chr14	68268803	68268813	GAGAGAGA(	INDEL	GAGAGAGA(	frameshift_s	ZFYVE26	14	exonic	ZFYVE26:uc010tta.2:exon10:c.1623_1631CTCTCTCTCTC,	-	-	-	-	-30	-	NaN	NaN	NaN	NA	0	None
