# Supplementary material for: Improving the efficacy of exome sequencing at a quaternary care referral centre: novel mutations, clinical presentations and diagnostic challenges in rare neurogenetic diseases
Source: J Neurol Neurosurg Psychiatry. 2021 Jun 8;92(11):1186–96. doi: 10.1136/jnnp-2020-325437 (PMC8522445; doi:10.1136/jnnp-2020-325437)
Supplement: Supplementary data [file jnnp-2020-325437supp003.pdf]

Supplemental data 2: Clinical summaries of patients diagnosed through exome sequencing.

An adult female (S.9) presented with a congenital myopathy involving delayed motor milestones, early onset progressive generalized weakness prominently affecting axial muscles, and loss of independent ambulation by the age of 8 years. She needed assistance for the activities of daily living. She reported multiple surgeries involving posterior spinal fusion and tendon transfers at the hip and the ankles. There was a history of multiple hospital admissions for pneumonia and bronchitis. She did not require ventilatory support or enteral feeding. The EMG revealed irritable myopathy. A vastus lateralis muscle biopsy during infancy was reported as non-specific myopathy. The cardiac echo and ECG were normal. She had been on prednisone for several years without clear beneficial effects. She was also diagnosed with hypohidrotic ectodermal dysplasia of undetermined etiology from the age of 2 years.

S.18 An adult male (S.18) presented with a two year history of bilateral distal upper extremity weakness, which resulted in difficulty playing musical instruments and frequently dropping of items from his hands. Atrophy was appreciated in the bilateral interossei muscles. Sensory testing in the distal extremities was normal. Muscle biopsy of the deltoid muscle showed angulated, atrophic muscle fibers. Testing of the unaffected parents determined that the mutation was *de novo* and not found in an unaffected sibling.

A late-aged female (S.4), and adult sisters (F3.1, F3.2) presented with slowly progressive childhood-onset LGMD. Parents were not blood relatives. They had retained independent ambulation and did not require ventilatory support or enteral feeding. There was no history of seizures or cognitive impairment. The cardiac echo and ECG were normal. These patients had a vastus lateralis muscle biopsy during their teenage years which was reported as chronic myopathy, but the muscle tissue was not available for further evaluation. The T1w skeletal muscle MRI of the F3.1 patient showed striated appearance with increased outer rim of signal in the vastus lateralis, soleus and gastrocnemius muscles, and increased signal around central facia and outer rim of the rectus femoris muscle (figure 3).

An adult female (S.13) presented with muscle weakness and atrophy in her hands and legs. Weakness was first noted in her toes at age 2, and she began using ankle foot orthoses at age 4. She was never been able to run, although she was able to walk as a child. Hand weakness was first noticed at age 6. Her disease slowly progressed and by age 30 she was unable to walk and began using a motorized wheelchair for mobility. On examination she had no voluntary movements of the toes and ankles with just trace movements in knee flexion and extension after the elimination of gravity. In contrast an adult male (F.5.1) and his maternal aunt (F.5.2) presented with milder and later onset CMT2A. Patient F.5.1's symptoms started in his mid 20s with weakness and discomfort in the toes bilaterally. On examination he was found to have pes cavus, hammertoes, distal atrophy, and weakness with toe flexion bilaterally. Sensation to vibration and pinprick was reduced in the distal lower extremities. Nerve conduction studies showed

reduced common peroneal motor amplitudes. Patient F.5.2 presented with bilateral lower extremity weakness that began in her mid 50s and was found to have pes cavus, atrophy of the calf and ankle muscles, and weakness in the toes and ankles. She had a normal sensory examination.

A late-aged male (S.7) presented with childhood onset Laing distal myopathy. His exam showed distally predominant weakness with severe involvement of toe, finger and wrist extension, and ankle dorsiflexion. The T1w skeletal muscle MRI showed tibialis anterior and extensor digitorum longus as the most affected muscles, but rectus femoris was spared (figure 3). Deltoid muscle biopsy showed chronic myopathy. The cardiac echo, ECG, and supine to sitting pulmonary function tests were normal.

A late-aged female (S.10) presented with gait imbalance and frequent sudden falls in her late 40s often resulting in injuries. Distal muscle weakness was first noted in her 50s and progressed to loss of independent ambulation by the late 60s. Her exam was notable for bifacial weakness, generalized muscle atrophy, hand tremor, severe generalized weakness and absent tendon reflexes. A needle EMG showed myopathic changes and nerve conduction studies showed absent sensory nerve action potentials and reduced amplitudes of compound motor action potentials in the upper and lower limbs. The vastus muscle pathology was notable for marked increase in internal nuclei and scattered pyknotic clumps without evidence of degeneration or regeneration. Fiber type proportion was normal. Audiologic studies showed sensorineural hearing loss.

An adult female (S.15) presented with juvenile onset distal weakness that initially started in her distal upper extremities. She underwent IVIG treatment which did not result in any improvement. On examination she was found to have atrophy of the first dorsal interosseous muscle and valgus deformity of the 5th upper extremity digits bilaterally. Weakness was appreciated with finger flexion and abduction, ankle dorsiflexion, and toe extension. Sensory examination was normal. Nerve conduction studies showed reduction of the compound muscle action potential amplitudes with fibrillation potentials and reduced motor unit recruitment appreciated in the distal upper extremities on electromyography.

A late-aged male (S.14) presented with distal weakness and significant loss of vibratory sensation in the lower extremities. He had symptoms of fatigue, muscle stiffness, and paresthesia. EMG showed denervation. The CAG repeat testing for spinal and bulbar muscular atrophy was negative. An adult female (S.15) presented with a juvenile onset MND. On examination, she had MRC grade weakness of 3 on finger flexion and grade 4 weakness with ankle dorsiflexion and toe extension.

An adult male (S.2) presented with a late-onset distal myopathy. He reported a trouble walking on cobblestone roads and on up hills in his 50s. He noted difficulty in walking on the toes, inability to run, and calf atrophy. The exam showed mild weakness of the ankle dorsiflexion and knee flexion (the MRC scale 4+/5). He was able to rise from a chair and climb steps without support. The T1w skeletal muscle MRI showed increased signal prominently in the adductor magnus, biceps femoris, semimembranosus, soleus, and

gastrocnemius muscles, followed by the semitendinosus, vastus, and tibialis anterior muscles (figure 3). The vastus lateralis muscle biopsy showed mild chronic myopathy with rimmed vacuoles in a few muscle fibers.

An adult female (S.3) born to consanguineous parents (second cousins, once removed) presented with childhood onset distal myopathy. She reported foot drop resulting in falls beginning at 5 years of age. Muscle weakness progressed to involve the upper limbs with distal predominance. In her 40s, she began using a motorized chair, and nightly BiPAP for respiratory weakness. The cardiac echo and ECG were normal. The vastus lateralis muscle biopsy showed chronic myopathy.

An adult female (S.16) born to consanguineous parents (first cousins, once removed) presented with abnormal asymmetric facial features, cognitive dysfunction, chronic diarrhea during childhood, and juvenile-onset cataracts. She had trouble keeping up with peers scholastically. Her exam showed asymmetric hypoplasia of facial structures, high arch palate, short neck, hammer toes, high plantar arches, kinetic postural hand tremor, atrophy of small muscles in the hands and feet, mild distal muscle weakness in the upper and lower limbs (MRC grade 4), glove and stocking hypesthesia, pathologically brisk knee jerk response (3+), and absent ankle jerk response bilaterally. She had trouble walking on the toes and heels and tandem. Cerebellar signs, spasticity, and subcutaneous xanthomas were absent. Neuropsychological testing indicated low average to borderline ability in several domains including verbal comprehension, working memory, perceptual reasoning, and processing speed (supplementary figure 3).

Nerve conduction studies showed a sensorimotor demyelinating neuropathy, predominantly in the lower limbs. The brain MRI showed T2-FLAIR hyperintensities in the dentate nuclei and adjacent cerebellar white matter bilaterally (figure 3). The skeletal survey using conventional x-ray showed 11 thoracic vertebrae with ribs (supplementary figure 3).

Two adult brothers (F2.1 and F2.2) presented with juvenile onset weakness. Parents were second cousins. Initial presentation was weakness in the biceps muscle during the teenage years, which progressed to involve the limb girdle, finger extensors, and neck flexors in their 30s. They reported fatigue and muscle cramps in the limbs that worsened on activity, a waddling gait, and shortness of breath. Prednisone treatment resulted in improvement and the steroid dose was adjusted according to symptom severity. Continuous follow up for 40 years was arranged with the same neurologist, and steroid taper was not tolerated. Examination was notable for weakness and atrophy in the biceps and mild weakness of finger extension and hip flexion (the MRC grade 4). EMG showed myopathic potentials and the 2-5 Hz repetitive nerve stimulation test showed 9-17% decrement in the orbicularis oculi muscle.

An adult male (F1.1) presented with progressive deterioration in contrast, color perception, and blurring of vision in both eyes for 3 years. He also reported burning pain and impaired touch sensation in his feet and lower legs, muscle cramps, hammer toes, hearing loss, fatigue, urinary urgency, erectile dysfunction, and impaired attention. He was initially diagnosed elsewhere with multiple sclerosis and treated with interferon

beta-1a for two years but continued to have worsening. His exam showed reduced visual acuity, lack of color vision, and optic disc pallor suggestive of bilateral optic atrophy and a left superior homonymous visual field defect suggestive of a post chiasmal lesion. He had postural kinetic hand tremor, spasticity in the lower limbs, mild weakness in toe extension (MRC grade 4), decreased perception of temperature, pinprick, proprioception, and vibration, diminished reflexes in the upper limbs (1+), pathologically brisk knee jerk response (3+), and ankle clonus. Optical coherence tomography showed abnormal thinning of retinal nerve fiber layer bilaterally, indicating optic nerve axon loss (figure 3). Brain MRI showed multiple foci of increased signal in the cerebral white matter and the brainstem on T2-FLAIR sequences without contrast enhancement (figure 3), which remained stable over 4 years of follow up. Nerve conduction studies indicated sensory greater than motor axonal neuropathy, predominantly in the lower limbs. The sweat responses measured using the Q-Sweat suggested preserved sympathetic post ganglionic cholinergic function. The auditory brainstem response study was abnormal, characterized by prolonged absolute and interpeak latencies (III, V; abnormal interaural wave V), indicating dysfunction of the auditory nerve and auditory brain stem tract. His younger sister (F1.2) had a similar clinical presentation.

Two family members (F.4.1; F.4.2) presented with bulbar weakness, dysarthria, and facial diplegia. The proband had onset of weakness in her late fifties with bilateral cataracts, dry skin and eyes, and peripheral sensory neuropathy. Her son had a similar presentation of facial weakness with an earlier onset in his mid-forties. On examination,

there were fasciculations in the muscles of the face, ptosis, and reduced vibratory sensation. The atypical duration of the disease progression, sensory involvement, and absence of upper motor neuron involvement made the diagnosis of MND less likely.
